# A unique approach to monitor stress in coral exposed to emerging pollutants

**DOI:** 10.1038/s41598-020-66117-3

**Published:** 2020-06-15

**Authors:** Didier Stien, Marcelino Suzuki, Alice M. S. Rodrigues, Marion Yvin, Fanny Clergeaud, Evane Thorel, Philippe Lebaron

**Affiliations:** Sorbonne Université, CNRS, Laboratoire de Biodiversité et Biotechnologies Microbiennes, USR3579, Observatoire Océanologique, 66650 Banyuls-sur-mer, France

**Keywords:** Environmental impact, Analytical chemistry, Bioanalytical chemistry

## Abstract

Metabolomic profiling of the hexacoral *Pocillopora damicornis* exposed to solar filters revealed a metabolomic signature of stress in this coral. It was demonstrated that the concentration of the known steroid (3β, 5α, 8α) -5, 8-epidioxy- ergosta- 6, 24(28) - dien- 3- ol (**14**) increased in response to octocrylene (OC) and ethylhexyl salicylate (ES) at 50 µg/L. Based on the overall coral response, we hypothesize that steroid **14** mediates coral response to stress. OC also specifically altered mitochondrial function at this concentration and above, while ES triggered a stress/inflammatory response at 300 µg/L and above as witnessed by the significant increases in the concentrations of polyunsaturated fatty acids, lysophosphatidylcholines and lysophosphatidylethanolamines. Benzophenone-3 increased the concentration of compound **14** at 2 mg/L, while the concentration of stress marker remained unchanged upon exposition to the other solar filters tested. Also, our results seemed to refute earlier suggestions that platelet-activating factor is involved in the coral inflammatory response.

## Introduction

Coral reefs are experiencing an unprecedented planet-wide decline^1^. This decline has been attributed to several anthropogenic factors, including global warming, overfishing, and pollution. Widely used for skin protection against cancer, solar filters are regularly released in the sea from populated coastal zones or in sites dedicated to touristic activities including a bathing zone. Nonetheless, the impacts of solar filters on corals have been relatively understudied to date. An early article from Danovaro and coworkers^2^ demonstrated that solar filters can induce coral bleaching by promoting viral infections. More recently, it was shown that several UV filters in the benzophenone class, along with octocrylene (OC), exert direct detrimental effects on corals^3–7^. Additionally, it has been shown that other ingredients in sunscreens and cosmetics exacerbate the toxicity of the UV filters OC and octinoxate^8^, while Fel *et al*. reported that many UV filters, including OC have little or no effect on corals^9^.

According to Downs *et al*., benzophenone-3 (BP3) induced concentration-dependent planulae coral bleaching, DNA-AP lesions, ossification of the planula, and planulae mortality, and these adverse effects were exacerbated by light^3^. In our previous work, we compared the metabolomic profiles of exposed and unexposed corals and demonstrated that OC triggers mitochondrial dysfunction that results in the accumulation of acylcarnitines^7^. Also of great concern were both the accumulation of OC derivatives in coral tissues and the concentration at which the toxicity was detected. In a 1-week exposure experiment, 19 OC derivatives were found in the coral tissue, and the response-inducing concentration was 50 µg/L, a concentration only 5 to 10 times higher than the highest environmental concentrations reported in the literature^10,11^. Since wild corals are exposed to solar filters for longer periods of time, it has been hypothesized that OC does impact corals in areas where it is continuously released. Other groups have also described the accumulation of UV filters in coral tissues^8,12,13^. These data further increase the interest of studying coral response to pollutants.

On July 2, 2018, and beginning January 1, 2021, Hawaii banned the sale or distribution of sunscreens containing oxybenzone or octinoxate in its territory^14^. Lately, Palau restricted the sale and use of reef-toxic sunscreens^15^. In that ruling, reef-toxic sunscreens were BP3, octinoxate and OC, the manufacturing, importation or sale of which will be prohibited in the Republic of Palau after January 1, 2020. In the current context, where national legislations are evolving to promote more sustainable tourism while little is known on the impact of UV filters on coral, it was key to evaluate more solar filters and to introduce a practical reliable tool to quantify coral responses to pollutants, while considering the public health importance of sunscreens.

In the current work, we studied the impact of 10 UV-filters on coral *Pocillopora damicornis* using untargeted metabolomic analysis in order to contribute to a better understanding of coral stress response to emerging pollutants.

## Results and discussion

### UV filters

The UV filters used in this study are listed in Table 1. All are approved in the European Union as cosmetic ingredients. Five of them are not approved by the FDA for human use but are often approved in other countries around the world, including those with significant coral reef areas such as France (4^th^ most areas) and Australia (2^nd^ most areas)^16^. Overall, the compound classes are somewhat diverse, with 5 classes for 10 solar filters.

**Table 1:** UV filters tested.

Abbr.	Name	Alternative names	Cmpd. class	CAS #	Formula	Maximum concentration in final product^a^		
						USA	EU	Aus.
OC	Octocrylene		Acrylate	6197-30-4	C_24_H_27_NO_2_	10%	10%	10%
MBBT	Methylene bis-benzotriazolyl tetramethylbutylphenol	Bisoctrizole, Tinosorb M, Milestab 360	Benzotriazole	103597-45-1	C_41_H_50_N_6_O_2_	n.a.	10%	10%
BP3	Benzophenone-3	Oxybenzone	Phenone	131-57-7	C_14_H_12_O_3_	6%	6%	10%
BM	Butyl methoxydibenzoylmethane	Avobenzone	Phenone	70356-09-1	C_20_H_22_O_3_	3%	5%	5%
DHHB	Diethylamino hydroxybenzoyl hexyl benzoate	Uvinul A Plus	Phenone	302776-68-7	C_24_H_31_NO_4_	n.a.	10%	10%
ES	2-Ethylhexyl salicylate	Octyl salicylate, Octisalate	Salicylate	118-60-5	C_15_H_22_O_3_	5%	5%	5%
HS	Homosalate		Salicylate	118-56-9	C_16_H_22_O_3_	15%	10%	15%
BEMT	bis-Ethylhexyloxyphenol methoxyphenyl triazine	Bemotrizinol, Tinosorb S, Escalol S	*s*-Triazine	187393-00-6	C_38_H_49_N_3_O_5_	n.a.	10%	10%
DBT	Diethylhexyl butamido triazone	Iscotrizinol, Uvasorb HEB	*s*-Triazine	154702-15-5	C_44_H_59_N_7_O_5_	n.a.	10%	n.a.
ET	Ethylhexyl triazone	Uvinul T150, Octyl triazone	*s*-Triazine	88122-99-0	C_48_H_66_N_6_O_6_	n.a.	5%	5%

^a^ USA: United States of America, EU: European Union, Aus.: Australia. n.a.: not approved.

### Compared effect of OC and ES on coral metabolomes

In the current study, *P. damicornis* nubbins were exposed to ES for 7 days at concentrations of 5, 50, 300 and 1000 µg/L. As with OC previously, the extracts prepared from the coral nubbins exposed to ES were analyzed by UHPLC-ESI^+^-HRMS^2^ and compared with control experiment^7^.

First, unlike what happened with OC, ES or ES-analogs do not seem to accumulate in the coral, although both compounds possess a 2-ethylhexyl side chain. Our hypothesis is that the ester group in OC is more stable than in ES. ES would then be degraded by carboxylesterase-mediated ester hydrolysis^17^ or via the bacterial *ortho* degradation pathway^18,19^ while OC is degraded by hydroxylation of the 2-ethylhexyl chain and subsequent grafting of fatty acids. As a result, lipophilic OC analogs accumulate in coral tissues, which might lead to further accumulation by the trophic chain.

**Figure 1:**
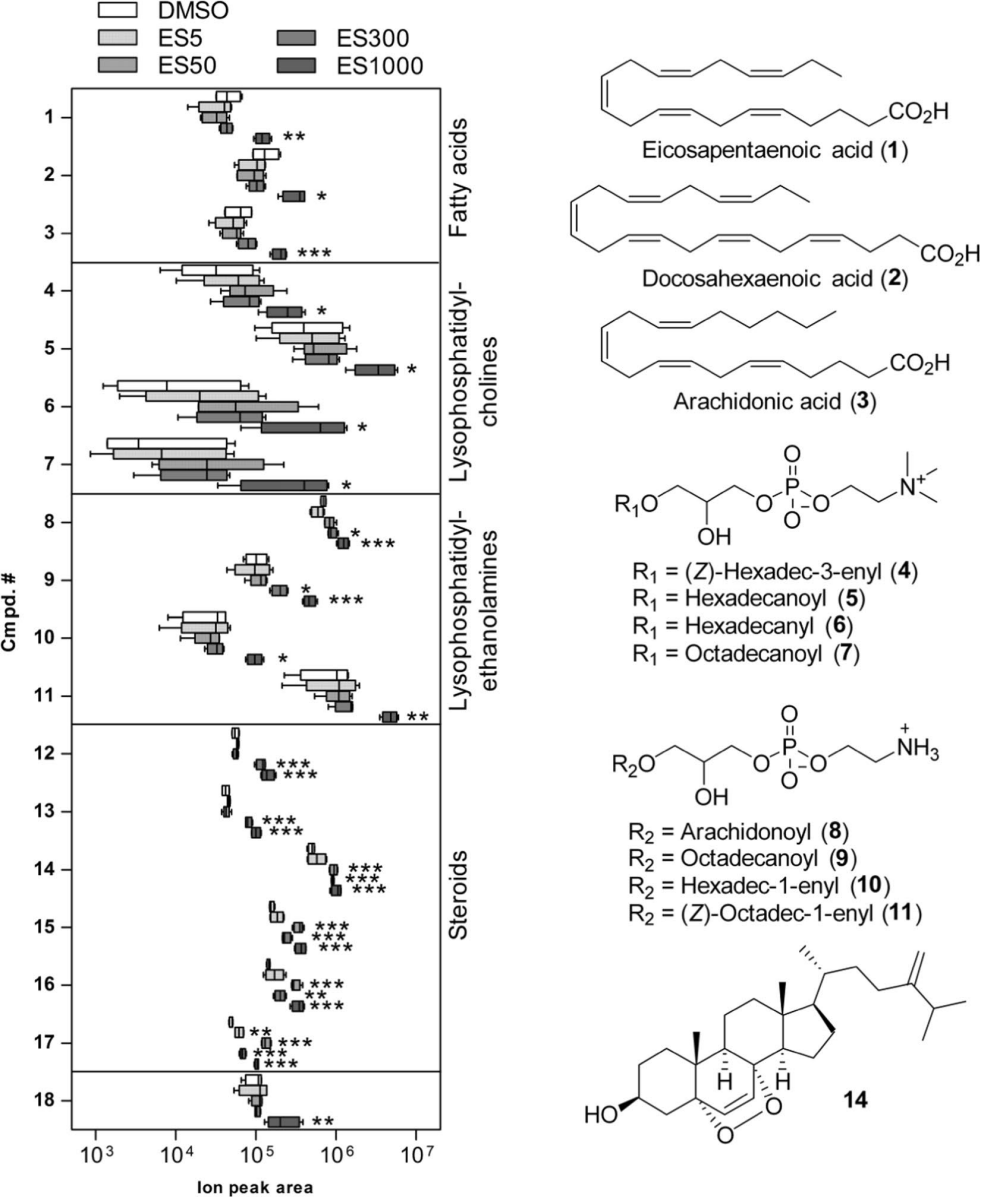
Identified upregulated metabolites and absolute integration values for biomarkers **1**–**18** ion peaks. The X-axis is in Log scale. For each ion, the leftmost point represents the minimal value, and the rightmost point the maximal one. The rectangular box represents the 25% quantile to 75% quantile ranges. The dark line shows the average of the distribution. Significance levels relative to DMSO were determined by an ANOVA followed by a Tukey HSD test. The differences were not significant unless otherwise stated. ***p < 0.001, **p < 0.01, *p < 0.05.

The metabolomic profiles were compared with those of control corals treated with DMSO only (0.25% v/v). Eighteen compounds are significantly upregulated at 1000 µg/L ES, and in some cases are also upregulated at lower ES concentrations (Table 2, Fig. 1).

**Table 2:** List of upregulated metabolites upon exposition to ES.

Cmpd. No.^a^	t_R_ (min)	*m/z*	Adduct	Molecular formula	Cmpd. name	CAS #	Cmpd. class
1 ☑	8.77	303.2319	[M + H]^+^	C_20_H_30_O_2_	Eicosapentaenoic acid	10417-94-4	Fatty acid
2 ☑	9.19	329.2475	[M + H]^+^	C_22_H_33_O_2_	Docosahexaenoic acid	6217-54-5	Fatty acid
3 ☑	9.33	305.2475	[M + H]^+^	C_20_H_32_O_2_	Arachidonic acid	506-32-1	Fatty acid
4	7.07	480.3449	[M + H]^+^	C_24_H_50_NO_6_P	1-*O*-(3*Z*-hexadecenyl)-*sn*-glycero-3-phosphocholine	339984-37-1	Lysophosphatidyl choline
5 ☑	7.25	496.3400	[M + H]^+^	C_24_H_50_NO_7_P	1-*O*-Hexadecanoyl-*sn*-glycero-3-phosphocholine	17364-16-8	Lysophosphatidyl choline
6 ☑	7.54	482.3605	[M + H]^+^	C_24_H_52_NO_6_P	1-*O*-Hexadecyl-*sn*-glycero-3-phosphocholine	52691-62-0	Lysophosphatidyl choline
7 ☑	8.17	524.3710	[M + H]^+^	C_26_H_54_NO_7_P	1-*O*-Octadecanoyl-*sn*-glycero-3-phosphocholine	19420-57-6	Lysophosphatidyl choline
8	6.84	502.2929	[M + H]^+^	C_25_H_44_NO_7_P	1-*O*-Arachidonoyl-*sn*-glycero-3-phosphoethanolamine	652149-09-2	Lysophosphatidyl ethanolamine
9 ☑	7.89	482.3242	[M + H]^+^	C_23_H_48_NO_7_P	1-*O*-Octadecanoyl-*sn*-glycero-3-phosphoethanolamine	69747-55-3	Lysophosphatidyl ethanolamine
10	7.36	438.2979	[M + H]^+^	C_21_H_44_NO_6_P	1-*O*-Hexadec-1′-enyl-*sn*-glycero-3-phosphoethanolamine	174062-72-7	Lysophosphatidyl ethanolamine
11 ☑	8.20	466.3292	[M + H]^+^	C_23_H_48_NO_6_P	1-*O*-(*Z*)-Octadec-1′-enyl-*sn*-glycero-3-phosphoethanolamine	174062-73-8	Lysophosphatidyl ethanolamine
12	9.01	481.2925	[M + Na]^+^	C_28_H_42_O_5_	Unidentified		Steroid
13	9.07	481.2931	[M + Na]^+^	C_28_H_42_O_5_	Unidentified		Steroid
14 ☑^b^	11.02	451.3181	[M + Na]^+^	C_28_H_44_O_3_	5α,8α-Epidioxyergosta-6,24(28)-dien-3β-ol	55688-50-1	Steroid
15	11.63	451.3181	[M + Na]^+^	C_28_H_44_O_3_	Unidentified		Steroid
16	12.30	465.3338	[M + Na]^+^	C_29_H_46_O_3_	Unidentified		Steroid
17	13.71	377.3200	Frag?^c^	Unknown	Unidentified		Steroid
18	6.70	562.3739	[M + H]^+^	C_32_H_51_NO_7_	Unidentified		unknown

^a^Check marks indicate that the structure was confirmed by comparison with a commercial standard. For **14**, see b.

^b^Structure confirmed by NMR.

^c^The molecular ion was not found, reported experimental *m*/*z* might correspond to a fragment.

Compounds **1**–**3** are the polyunsaturated fatty acids eicosapentaenoic, docosahexaenoic and arachidonic acid (AA, Fig. 1). The structures were determined based on the molecular formulas and examination of fragmentation spectra with MS-Finder. Identification was confirmed by comparison of retention times and MS/MS spectra with those of commercially available standards. These compounds were significantly upregulated at 1 mg/L ES and were not affected at lower concentrations (Fig. 1). Until recently, ω3 long-chain polyunsaturated fatty acids (PUFAs) would have been considered as Symbiodiniaceae^20^ metabolites. Vertebrates lack the ωx desaturases necessary for PUFA synthesis and it was largely accepted that all animals should lack it too. However, it is now well established that many marine invertebrates, including corals (*P. damicornis* among them), have acquired ωx desaturases by horizontal gene transfer^21–23^.

In mammals, ω3 PUFAs, and in particular AA, that come from the diet are stored and are involved in the inflammatory cascade after cleavage from phosphatidylcholine by phospholipase A2 (PLA2) and transformation into several derived signaling molecules such as leukotrienes and prostaglandins. In corals, increased production of eicosanoids, linked to an increase in the expression of an allene oxide synthase-lipoxygenase (AOS-LOXa) was shown for the soft (octo)coral *Capnella imbricata* in response to mechanical injury and thermal stress^22,24^. In stony (hexa)corals, eicosanoids such as 8-hydroxyeicosatetraenoic acid (8-HETE), (*S*,5*Z*,11*Z*,14*Z*)-8-hydroxy-9-oxoicosa-5,11,14-trienoic acid and (5*Z*,12a,14*Z*)-9-oxo-prosta-5,10,14-trien-1-oicacid were also observed, both in extracts of whole coral tissue, and after incubation of ^14^C-labeled AA with tissue homogenates of *Acropora millepora*, *A. cervicornis* and *Galaxea fascicularis*^23^. These results, combined with previous transcriptomic analysis showing upregulation of enzymes linked to eicosanoid production in corals undergoing black disease or thermal stress^25,26^, led to the hypothesis that the production of eicosanoids could also be associated with stress in stony corals^23^. Finally, experiments with host switching using the sea anemone *Exaiptasia pallida* also show evidences towards increases of eicoisanoids after colonization with a heterologous Symbiodiniaceae^27^.

Our *blastp* analysis of the *P. damicornis* genome identified several enzymes linked to the production of eicosanoids, including cytosolic and secreted PLA2, AOS-LOXs, 5-lypooxygenases and a leukotriene A4 hydrolase, confirming previous results in the literature (Supplementary Table S1)^23,26^. However, other than PUFAs, we could not detect any of the eicosanoids, leukotrienes or prostaglandins in *P. damicornis* extracts, nor could we identify a leukotriene receptor based on *blastp* searches using the mouse LTB4R and CYSLTR1 as queries. The closest seven-transmembrane G-protein coupled receptors in *P. damicornis* were related to receptors in the alpha and beta groups of rhodopsin family (defined by Fredriksson *et al*.)^28^ and not the gamma and delta groups as other known leukotriene receptors.

Altogether, these results suggest that ω3 PUFAs concentration increased likely in the context of a stress process triggered by ES. This effect was only noticeable at the highest exposition concentration, but the downstream signaling molecules or putative receptors were absent or not measurable in our study.

Compounds **4**–**7** have very similar MS/MS fragmentation patterns with common peaks at *m*/*z* 60.0808 ([Me_3_NH]^+^), 86.0964 ([Me_3_N-CH = CH_2_]^+^), 104.1070 ([Me_3_NCH_2_CH_2_OH]^+^), 124.9998 ([H_2_O_3_PO-CH = CH_2_]^+^), and 184.0733 ([Me_3_NCH_2_CH_2_OPO_3_H_2_]^+^), showing evidence of the presence of a phosphocholine subunit (Supplementary Scheme S1). The molecular formula completed the structures as those of lysophosphatidylcholines (LPCs). In the collision-induced dissociation of sodiated **5** (Supplementary Fig. S14), the 5:1 peak intensity ratio of product ions at *m*/*z* 104 and 147 indicated that **5** was an *sn*-1-LPC regioisomer^29^. The identification of compounds **5**–**7** was finally unambiguously established by comparing the retention times and MS/MS spectra with those of commercially available standards.

As for ω3 PUFAs, these compounds were significantly upregulated only at 1 mg/L ES (Fig. 1). LPCs are widespread in the animal kingdom^30^. In mammals, LPCs are proinflammatory lipids upregulated in an event of inflammation, inducing pro-inflammatory cytokine secretion and activating B lymphocytes^31–33^. LPCs stimulate time- and concentration-dependent release of arachidonic acid (compound **3**) in human coronary artery smooth muscle cells^33^. Interestingly, it has been proposed that the coral immune system is similar to that of higher organisms, including mammals^34–37^. In *Porites* sp., platelet-activating factor (PAF) concentration has been reported to increase during interactions with *Acropora cervicornis*^30^ as has the expression of a gene coding for a putative LPC acetyltransferase, the protein responsible for converting LPC to PAF, in response to stress and inflammation in mammals^38^. PAF was not detected in our study, even though the genome of *P. damicornis* codes for an acyl transferase with high homology to the mammalian LPC acyltransferase, containing the motif HXXXXD responsible for acyltransferase activity, and a putative acyl-acceptor binding pocket domain (Supplementary Table S1). Remarkably, compound **7** is an isomer of PAF and could easily be mistaken for PAF. In our study, its structure was confirmed by comparison with standards of **7** and PAF (Supplementary Figs. S2 and S3). The fragmentation product at *m/z* 104 from [**7** + H]^+^ further confirms the identification of **7** as the 1-*O*-octadecanoyl-*sn*-glycero-3-phosphocholine^39,40^. In addition, unlike what was reported for *Acropora digitifera*, we did not identify a gene coding PAF receptor based on *blastp* searches using the human PTAFR receptor as a query (Supplementary Table S1). The closest seven-transmembrane G-protein-coupled receptors in *P. damicornis* were related to cholecystokinin receptors in beta groups of the rhodopsin family (defined by Fredriksson *et al*.)^28^ and not in the delta group as the PTAFR receptor. Finally, our results show that the expression of LPCs fluctuates widely between replicates (Fig. 1) and, therefore, cannot be considered as good stress indicators. The signal was weakly significant (*p* < 0.05 for all 4 LPCs) at 1000 µg/L ES and was not significant at lower concentrations.

**Figure 2:**
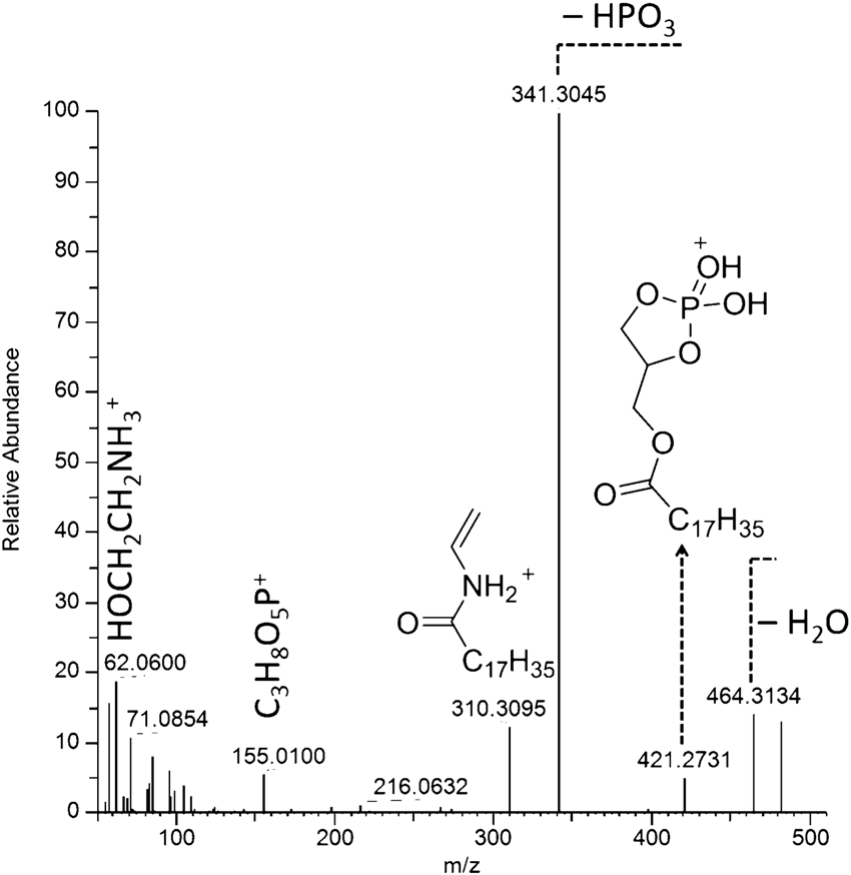
Collision-induced MS/MS spectrum of the compound **9** pseudomolecular ion and identification of the key fragment ions.

**Figure 3:**
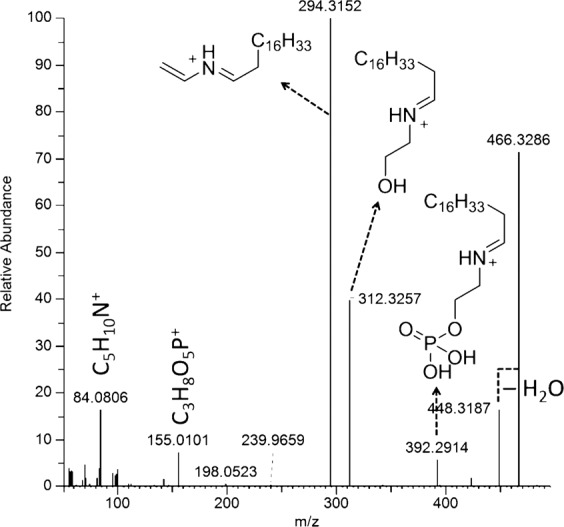
Collision-induced MS/MS spectrum of the compound **11** pseudomolecular ion and identification of the key fragment ions.

Compounds **8**–**11** were identified as lysophosphatidylethanolamines (LPEs) based on molecular formulas, MS/MS fragmentation patterns, and structures proposed by CD for compounds **8**–**10**. Compounds **8** and **9** had very similar fragmentation patterns. For example, **8** is characterized by a series of products resulting from the successive loss of water (*m/z* 464.3134), ethanolamine (*m/z* 421.2731), and HPO_3_ (*m/z* 341.3045, 100%) (Fig. 2, Supplementary Scheme S2). The ion at *m/z* 310.3095 (330.2775 for **9**) corresponds to a product formed by transposition of the acyl unit to the amino group followed by β-elimination of the phosphate ester. This transposition only occurs for the *sn*-1-LPE regioisomers^41^. The structure of compound **9** was unambiguously confirmed by comparison with a commercial standard. The concentration of compounds **8** and **9** increased significantly at 300 µg/L ES and above (Fig. 1). Compounds **10** and **11** are close analogs as demonstrated by their very similar MS^2^ fragmentation patterns, in which the migration of the enol ether chain on the amino group leads to the major fragmentation products (Fig. 3, Supplementary Scheme S3). The identification of compound **10** was confirmed by comparison with a commercially available standard. Compounds **10** and **11** were significantly upregulated at 1000 µg/L ES.

It has been well established that LPEs and LPCs are produced by hydrolysis of phosphatidylethanolamines and phosphatidylcholines, releasing arachidonic acid or other fatty acids linked to the *sn*-2 position. This reaction is usually mediated by a phospholipase A2. An increased phospholipase A2 activity in coral exposed to ES would account for the observed concomitant increase of ω3 PUFAs (**1**–**3**), LPCs (**4**–**7**) and LPEs (**8**–**11**). As discussed above *P. damicornis* genome mining revealed that the genome of this species codes for a number of secretory (sPLA2) and cytosolic PLA2 (cPLA2) presumably involved in this mechanism. Similar to in mammals^42,43^, sPLA2 upregulation in coral is visibly associated with the activity of the innate immune system and/or a stress response^26,38^. Increases in PLA2 expression and arachidonic acid was also shown after host switching with a heterologous Symbiodiniaceae with sea anemone *Exaiptasia pallida* even though that study did not see differences in LPEs and LPCs^27^. Overall, the metabolomic response of coral after exposition to ES resembles the signature of a stress/inflammatory process triggered by ES.

To the exception of compound **14**, compounds **12**–**17** (Fig. 1) were detected with somewhat small response peaks, and it was not always possible to obtain MS/MS spectra. Compounds **12**–**17** seemed related based on their molecular formulas and on their ionization and fragmentation patterns. Interestingly, the differential analysis showed that all these compounds were significantly upregulated when the coral was exposed to ES at concentrations ranging from 50 to 1000 µg/L. It turned out that the concentration of **14** also increased upon exposition to OC^7^, and at this point, it was clearly necessary to determine unambiguously the structure of compound **14**. Extensive fractionation allowed for the isolation of **14** in its pure form, which was ultimately identified by NMR as the known steroid (3β,5α,8α)-5, 8-epidioxy-ergosta-6,24(28)-dien-3-ol. Compound **14** along with many epidioxy sterol analogs have been described essentially in marine invertebrates, including several cnidarians^44–47^. The role and fate of this compound in invertebrates remains an unresolved question. However, steroids are present in the whole animal kingdom. They regulate life cycles, mating, and development. In Cnidarians, bioregulatory pathways and hormonal-like signaling remain largely uncharacterized^48–50^. Nonetheless, a family of nuclear receptors has been found to bind the ancient hormone paraestrol A^50,51^. According to Khalturin *et al*., Cnidarian steroids can be transported in the digestive tract and through the mesoglea, and may be involved in intercellular communication. Here, compound **14** concentration increased in coral exposed to pollutants, and these pollutants eventually triggered a stress response witnessed by the increased production of specific lipids. We hypothesize that the increased concentration of this class of steroids – and in particular the major compound **14** – is a signal triggering a coral inflammatory-like response. Owing to the very small standard deviation within replicates (Fig. 2), **14** could be considered as a good marker of stressed corals. In our experiments, the concentration of **14** significantly increased with both ES and OC at 50 µg/L, indicating that both solar filters had a negative impact on coral. However, ES triggered a stress/inflammatory response while OC also specifically altered mitochondrial function^7^.

Last, it should be mentioned that the relative concentration of five metabolites decreased when the coral was exposed to ES at 1 mg/L (Supplementary Figs. S1, S51–S61). These metabolites have been identified as four monogalactosyl diacylglycerols (MGDGs **19**–**22**) and one cerebroside (**23**). In MS^2^, MGDG fragmentation pattern shows the length and number of unsaturation of each acyl chain. The relative position of the acyl chains can be assigned based on the relative peak intensities of the sodium adduct fragmentation products. The peak intensity of the product ion resulting from the loss of the *sn*-1 fatty acid is always higher than the one resulting from the loss of the *sn*-2 fatty acid^52^. Although cerebrosides are widely distributed in the eukaryotes, galactolipids including MGDGs are the main components of plant chloroplast membrane lipids^53,54^. Decreased MGDGs concentration might point towards an effect of the UV filter on the coral symbiont. It is possible that the coral had begun to bleach although bleaching was not visible to the naked eye. However, specific toxicity mechanism unbalancing Symbiodiniaceae metabolism or any process disrupting algal symbiont metabolite translocation cannot be ruled out.

### Effect of BEMT, BM, BP3, DBT, DHHB, ET, HS, and MBBT

**Figure 4:**
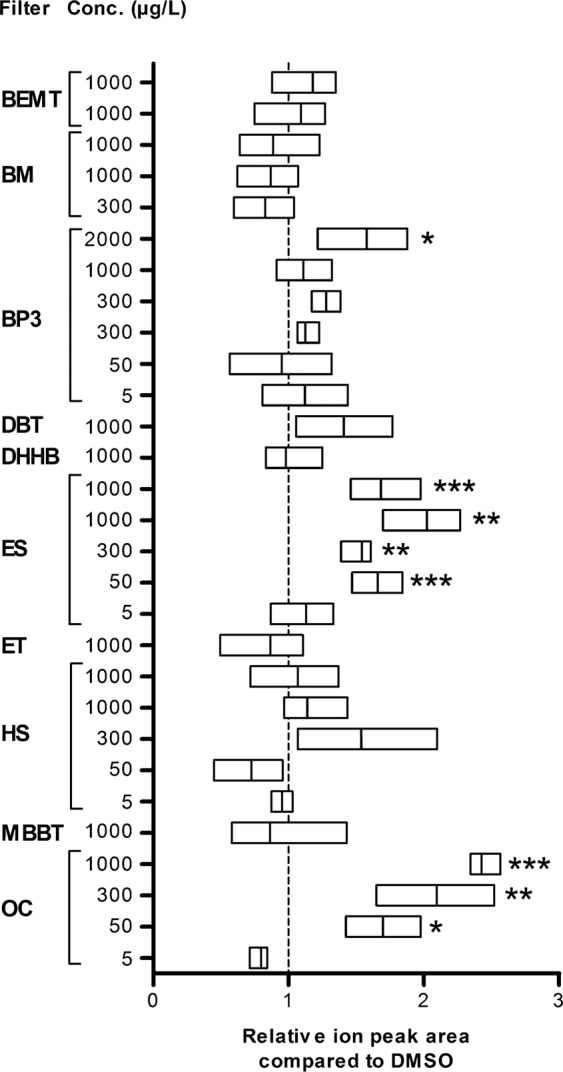
Relative integration values for compound **14** ion peak in exposed coral compared to control animals. The vertical dashed line illustrates the 1:1 ratio. The rectangular box represents the min to max ranges. The dark line shows the average of the distribution. Significance levels relative to DMSO were determined by an ANOVA followed by a Tukey HSD test. The differences were not significant unless otherwise stated. ***p < 0.001, **p < 0.01, *p < 0.05. The multiple data points for same concentrations are for repeat experiments.

The effect of 8 other solar filters was examined based on the comparison of global metabolomic profiles between exposed and unexposed coral, but it was also examined in light of the putative increased concentration of compound **14** in exposed corals. In general, the first step consisted of exposing coral at 1000 µg/L of each solar filter and testing lower concentrations when an effect was detected. BP3 was tested at 2000 µg/L as well because very high environmental concentrations of this compound have been occasionally detected^3^. The relative variations in compound **14** concentration are reported in Fig. 4.

We observed that BEMT, DBT, DHHB, ET and MBBT do not seem to affect the overall coral metabolome at the highest concentration tested. Specific measurement of the concentration of compound **14** in treated versus untreated coral confirmed the apparent innocuousness of these five molecules, as the concentration of **14** remained stable. It was found that the concentration of **14** increased at 2 mg/L BP3, indicating that BP3 most certainly affects wild coral at the highest published environmental concentration^3^. Coral exposure to BM does not induce an increase in the concentration of compound **14**. Nevertheless, the presence of undetermined ions in the global coral metabolome will require further investigation. Last, HS does not alter either the concentration of compound **14** or the overall metabolome of *P. damicornis*. However, polyps of coral exposed to HS at 1 mg/L were closed at the end of the assay, while those of control corals were not, as if the coral reacted although its metabolome was not significantly altered (Supplementary Fig. S61).

## Conclusion

This work establishes a metabolomic signature of stressed *P. damicornis*. In this coral, the known steroid (3β, 5α,8α)-5,8-epidioxy-ergosta-6,24(28)-dien-3-ol (**14**) is viewed as a steroid hormone that could trigger coral response to pollutants. OC was the most toxic of the tested UV filters. It induced coral stress response, while triggering mitochondrial dysfunction at 50 µg/L. Of concern was also the previously reported accumulation potential of possibly toxic coral-modified OC derivatives. ES comes second in terms of toxicity. ES triggered coral stress response at 50 µg/L, inducing a significant increase in the concentration of compound **14**. At 300 µg/L ES and above, the relative concentration of several PUFAs, LPCs and LPAs also increased. ES may also have induced partial coral bleaching at 1 mg/L although this remains to be firmly established. BP3 was also toxic at 2 mg/L in our assay. BEMT, BM, DBT, DHHB, ET, HS and MBBT did not affect coral metabolism at any of the concentrations tested, although in the case of HS and BM, further investigations are needed to evaluate their potential effects. More investigations are also needed to clarify the role of compound **14** in coral hormonal response to pollutants.

## Methods

### General experimental procedures

Nuclear Magnetic Resonance (NMR) spectra were recorded on a JEOL ECZ500R spectrometer equipped with a 5-mm inverse detection FG/RO Digital Autotune Probe. Chemical shifts (δ) are reported as ppm downfield from tetramethylsilane, and the coupling constants (*J*) are reported in Hertz. High-resolution MS/MS analyses were conducted with a Thermo UHPLC-HRMS system^7^. Analyses were performed in the electrospray positive ionization mode in the range of 133.4–2000 Da in the centroid mode. The mass detector was an Orbitrap MS/MS FT Q-Exactive focus mass spectrometer. The analyses were conducted in FullMS-data dependent MS^2^ mode. In FullMS, the resolution was set to 70,000, and the AGC target was 3.10^6^. In MS^2^, the resolution was 17,500, AGC target 10^5^, isolation window 0.4 Da, and stepped normalized collision energy 15/30/45 was used, with 15 s dynamic exclusion. The lock mass option was set for an ion at m/z 144.98215, corresponding to Cu(CH_3_CN)_2_^+^. For coral profiling and comparison of coral profiles with standards, the UHPLC column was a Phenomenex Luna Omega polar C-18 150 × 2.1 mm, 1.6 µm. The column temperature was set to 42 °C, and the flow rate was 0.5 mL.min^−1^. The solvent system was a mixture of water (solution A) with increasing proportions of acetonitrile (solution B), and both solvents were modified with 0.1% formic acid. The gradient was as follows: 2% B 3 min before injection; then from 1 to 13 min, there was a shark fin gradient increase of B up to 100% (curve 2), followed by 100% B for 5 min. The flow was diverted (not injected into the mass spectrometer) before injection, up to 1 min after injection. For fast analysis of the fractions of coral extract, the UHPLC column was a Thermo Scientific Accucore Vanquish C18 + 50 × 2.1 mm, 1.5 µm. The column temperature was set to 42 °C, and the flow rate was 0.5 mL.min^−1^. The gradient was as follows: 2% B 1 min before injection; then from 0 to 4 min, there was a shark fin gradient increase of B up to 100% (curve 2), followed by 100% B for 1 min. The flash chromatography was performed with a Teledyne ISCO CombiFlash Companion equipped with a Büchi FlashPure Ecoflex C18 50 µm 40 g column. The column was equilibrated with water:CH_3_CN 4:6. The flow rate was 40 mL/min. The injection was performed in the solid phase, with fraction F3 impregnated on C18 silica (1 mL). The gradient was 60% acetonitrile until 1 min after injection; then, from 1 to 20 min, there was a linear gradient increase of acetonitrile up to 100%, followed by 100% acetonitrile for 10 min. Effluents were collected in 30 s fractions. The preparative HPLC was conducted with a hybrid system mounted with a Reodyne manual injector, 2 Varian PrepStar 218 HPLC pumps, a Dionex Ultimate 3000 variable wavelength detector, and a Dionex Ultimate 3000 fraction collector. The column was a Phenomenex Luna C18 250 × 21.20 mm, 5 µm. The gradient was as follows: 90% acetonitrile from 0 to 8 min, 91% from 8 to 16 min, 92% from 16 to 32 min, and 100% for 13 min. The flow rate was 20 mL/min, and fractions were collected every 30 s. The second preparative HPLC was performed on small scale with a Dionex ultimate 3000 system equipped with a deaerator, an HPG-3200SD pumping device, an autosampler, a column oven, a diode array detector and a fraction collector. The column was a Phenomenex Luna C18 150 × 4.60 mm, 5 µm. The solvent was an isocratic mixture of water:CH_3_CN 15:85 modified with 0.1% formic acid. The flow rate was 1 mL/min, and fractions were collected every 30 s.

### Chemicals

The solar filters used in this study are listed in Table 1. BEMT, BM, BP3, and MBBT were purchased from Sigma-Aldrich, Saint-Quentin Fallavier, France. DBT, DHHB, ES, ET, HS, and OC were provided by Pierre Fabre Laboratories. Standards of platelet-activating factor (PAF) and compounds **1**–**3**, **5**–**7**, **9**, and **11** were purchased from AnalyticLab, Montpellier, France.

### Pocillopora damicornis

Fragments of the coral *P. damicornis* were collected in Oman in 2014 (CITES permit 37/2014). This procedure had no impact on the wild population as 1–5% of few coral colonies were collected. The coral was acclimated in tanks at the Banyuls Oceanological Observatory. New colonies were obtained in the laboratory from the fragments and used for our experiments. The corals were maintained in artificial sea water (ASW) prepared with reverse osmosis purified water and Reef Salt SeaChem salts. Salinity was adjusted to 36 g/L, pH = 8, and the temperature was set at 24 °C. All experiments were conducted with the same ASW.

### Exposition of coral to solar filters, extraction and metabolomic analyses

The coral exposition protocol, the extraction, the metabolomic profiling and the statistical analyses were conducted as described before for OC^7^. They are also reported in Supplementary Information. Five replicates were used for each condition, and in some cases, the conditions were repeated. The standard compounds were diluted in MeOH (≈ 10 µg/mL each, 1 µL injected) and were analyzed in the same conditions as the coral extracts for comparison of retention times, MS and MS/MS spectra. All standards were identical to the coral metabolites in retention times and MS/MS spectra (see supplementary information). Interpretations of the metabolomic data and MS/MS spectra were conducted with the help of Compound Discoverer 2.1 and FreeStyle 1.3 (Thermo Fisher Scientific, Villebon, France), and MS-Finder 3.04^55,56^. The same procedure as in Stien *et al*. (2019) was used for Compound Discover. Extracted ion chromatograms for compounds **1**–**18** along with ESI^+^-HRMS spectra, collision-induced dissociation spectra and comparison with commercial standards are provided in Supporting Information (Supplementary Figs. S4–S50).

### Isolation and characterization of compound 14

Approximately 200 2–5 cm-long coral nubbins were cut from the branch tips of mother colonies. The nubbins were placed in a large Erlenmeyer flask and covered with MeOH:CH_3_CN 1:1. After 24 h at room temperature, the flask was sonicated for 20 min, the coral pieces were removed by filtration, and the solvent was evaporated to give the crude coral extract (2.5 g). The crude extract was dissolved in the smallest amount DMSO possible and was purified by SPE with a phenomenex Strata C-18 150 mL column. The column was equilibrated successively with CH_3_CN and water. Elution was performed with water (300 mL, F1), with water:CH_3_CN 1:1 (300 mL, F2) and finally with MeOH:CH_2_Cl_2_ 8:2 (300 mL, F3). Fractions were evaporated and diluted in MeOH at 1 mg/mL for LC/MS analyses (1 µL injected). The target compound at t_R_ ≈ 11.02 min and *m*/*z* 395.3308 was detected in fraction F3 (0.29 g). Fraction F3 was purified by flash chromatography, providing 80 fractions. The analysis of the fractions demonstrated that the target compound was detected in fractions 45 to 63. These fractions were gathered and evaporated. For injection, fraction F3.45–63 (49.95 mg) was diluted in water:CH_3_CN 1:9 (2 mL). Preparative HPLC was performed twice with 1 mL injected. The targeted compound was concentrated in fractions 40 to 45, which were gathered and evaporated. The resulting fraction F3.45-63.40-45 (5.91 mg) was diluted in water:CH_3_CN 15:85 (1.5 mL) and was purified by small-scale preparative HPLC by portions of 250 µL (6 injections). The desired compound **14** was isolated in pure form from fractions 27–29 (0.9 mg).

### Analytical data for compound 14

5α,8α-Epidioxyergosta-6,24(28)-dien-3β-ol (**14**). ^1^H NMR (500 MHz, CDCl_3_): δ 0.81 (s, 3 H, H-18), 0.88 (s, 3 H, H-19), 0.94 (d, 3 H, *J* = 6.5 Hz, H-21), 1.02 (d, 3 H, *J* = 6.8 Hz, H-26), 1.03 (d, 3 H, *J* = 6.8 Hz, H-27), 1.21 (m, 1 H, H-17), 1.16 (m, 1 H, H-22a), 1.22 (m, 1 H, H-11a), 1.23 (m, 1 H, H-12a), 1.39 (m, 1 H, H-16a), 1.408 (m, 1 H, H-15a), 1.411 (m, 1 H, H-20), 1.22 (m, 1 H, H-11a), 1.496 (m, 1 H, H-9), 1.497 (m, 1 H, H-11b), 1.53 (m, 1 H, H-2a), 1.54 (m, 1 H, H-22b), 1.55 (m, 1 H, H-14), 1.63 (m, 1 H, H-15b), 1.69 (dt, 1 H, *J* = 13.5, 3.4 Hz, H-1a), 1.85 (m, 1 H, H-2b), 1.89 (m, 1 H, H-23a), 1.94 (m, 1 H, H-16b), 1.95 (m, 1 H, H-1b), 1.98 (m, 1 H, H-12b), 1.91 (dd, 1 H, *J* = 13.9, 11.7 Hz, H-4a), 2.09 (brdd, 1 H, *J* = 10.9, 4.7 Hz, H-23b), 2.11 (ddd, 1 H, *J* = 13.9, 4.9, 1.9 Hz, H-4b), 2.22 (heptd, 1 H, *J* = 6.8, 1.0 Hz, H-25), 3.97 (m, 1 H, H-3), 4.65 (brq, 1 H, *J* = 1.4 Hz, H-28a), 4.72 (brs, 1 H, H-28b), 6.24 (d, 1 H, *J* = 8.5 Hz, H-6), 6.51 (d, 1 H, *J* = 8.5 Hz, H-7); ^13^C NMR (125 MHz, CDCl_3_): δ 12.6 (C-18), 18.2 (C-19), 18.6 (C-21), 20.6 (C-15), 21.8 (C-27), 22.0 (C-26), 23.4 (C-11), 28.2 (C-16), 30.1 (C-2), 30.9 (C-23), 33.8 (C-25), 34.4 (C-22), 34.7 (C-1), 35.2 (C-20), 36.9 (C10), 37.0 (C-4), 39.4 (C-12), 44.7 (C-13), 51.1 (C-6), 51.6 (C-14), 56.3 (C-17), 66.5 (C-3), 106.1 (C-28), 130.7 (C-7), 135.4 (C-6), 156.6 (C-24); HR-ESI^+^-MS *m*/*z* found 429.3362 [M + H]^+^; calcd. for [C_28_H_45_O_3_]^+^: 429.3363; HR-ESI^+^-MS *m*/*z* 395.3307 (100, [M + H-H_2_O_2_]^+^), 429.3361 (41, [M + H]^+^), 377.3202 (41, [M + H-H_2_O_2_-H_2_0]^+^), 411.3255 (24, [M + H-H_2_0]^+^), 451.3180 (8, [M + Na]^+^); HR-ESI^+^-MS^2^ for [M + H]^+^ ion *m*/*z* 429.3357 (100), 81.0698 (45), 411.3251 (43), 95.0854 (33), 107.0853 (22), 69.0698 (21), 109.1011 (20).

### *P. damicornis* genome mining

To interpret the possible roles of the identified stress biomarkers, we queried a number of possible putative enzymes and receptors leading to the synthesis, modification and binding of different phospholipids, polyunsaturated fatty acids and oxylipins against proteins coded by the *P. damicornis* genome [NCBI genome 22550, ASM380409v1 reference annotation release 100/annotated proteins^36^] using the web-based *blastp* program of the NCBI and default parameters. Reciprocal *blastp* searches were also conducted using the web-based tool against the *UniProt/Swiss-Prot*, and *model organism (landmark)* databases, and in some specific cases, the conserved domains (CDD) search. Query sequences for putative enzymes were retrieved by text searches in the MetaCyc, Brenda and KEGG databases, and whenever possible, the closest (based on phylogenetic proximity) experimentally validated sequences were used. Receptor sequences for queries were identified by text searches in the GLASS-GPCR (https://zhanglab.ccmb.med.umich.edu/GLASS/index.html) database using the same selection criterion as for the enzymes.

## Supplementary information

Supplementary information.
