# Supplementary material for: A unique approach to monitor stress in coral exposed to emerging pollutants
Source: Sci Rep. 2020 Jun 15;10:9601. doi: 10.1038/s41598-020-66117-3 (PMC7295770; doi:10.1038/s41598-020-66117-3)
Supplement: Supplementary file 1 — Supplementary information. [file 41598_2020_66117_MOESM1_ESM.pdf]

Supporting information for

A unique approach to monitor stress in coral exposed to emerging pollutants

Didier Stien*, Marcelino Suzuki, Alice Rodrigues, Marion Yvin, Fanny Clergeaud, Evane Thorel, Philippe Lebaron

Sorbonne Université, CNRS, Laboratoire de Biodiversité et Biotechnologies Microbiennes, USR3579, Observatoire Océanologique, 66650 Banyuls-sur-mer, France

Table of Content

Experimental Section 5

Figure S1. Annotated volcano plot comparing the exposition of coral to ES at 1 mg/L to the control DMSO-exposed corals. Note that several ions (dots) can correspond to the same molecule. The red box frames the ions the concentration of which significantly increased upon exposition to ES. The horizontal limit of the box is for a p value of 0.05: all ions above this line are significantly up-regulated. The vertical limit of the box is for a Log2 fold changes of +1. The green box frames ions corresponding to down-regulated metabolites. Ions dot numbering refers to Table 1. n.i. is for 'not identified' (very small peaks or analytical noise). 7

Additional experimental section: the search for PAF in coral profiles 7

Figure S2. Compared extracted ion chromatograms at m/z 524.3711 for (top) the metabolomic profile of coral exposed to ES at 1000 µg/L, and (bottom) the PAF standard diluted at 2 µg/mL in MeOH.. 8

Figure S3. Compared MS^2 spectra of **7** and PAF pseudomolecular ions at Normalized Collision Energies of 30 and 45 %. The choline ion at theoretical *m/z* 104.1070 is absent or very small in $[PAF+H]^+$ MS^2 spectra. 8

Scheme S1. Proposed fragmentation scheme for compounds **4**-**7** pseudomolecular ions 9

Scheme S2. Proposed fragmentation scheme for compound **9** pseudomolecular ion........... 10

Scheme S3. Proposed fragmentation scheme for compound **11** pseudomolecular ion......... 11

Figure S4. Extracted ion chromatograms for eicosapentaenoic acid (**1**) pseudomolecular ion at *m/z* 303.2319 in standard (top) and coral extract (bottom) 11

Figure S5. Compared collision-induced dissociation of eicosapentaenoic acid (**1**) pseudomolecular ion at *m/z* 303.2319 in standard (top) and coral extract (bottom) 12

Figure S6. Extracted ion chromatograms for docosahexaenoic acid (**2**) pseudomolecular ion at *m/z* 329.2475 in standard (top) and coral extract (bottom) 12

Figure S7. Compared collision-induced dissociation of docosahexaenoic acid (**2**) pseudomolecular ion at *m/z* 329.2475 in standard (top) and coral extract (bottom) 13

Figure S8. Extracted ion chromatograms for arachidonic acid (**3**) pseudomolecular ion at *m/z* 305.2475 in standard (top) and coral extract (bottom).. 13

Figure S9. Compared collision-induced dissociation of arachidonic acid (**3**) pseudomolecular ion at *m/z* 305.2475 in standard (top) and coral extract (bottom) 14

Figure S10. ESI$^+$-HRMS spectrum of 1-*O*-(3*Z*-hexadecenyl)-*sn*-glycero-3-phosphocholine (**4**) in coral extract... 14

Figure S11. Collision-induced dissociation of 1-*O*-(3*Z*-hexadecenyl)-*sn*-glycero-3-phosphocholine (**4**) pseudomolecular ion at *m/z* 480.3446 in coral extract (bottom) 15

Figure S12. Extracted ion chromatograms for 1-*O*-hexadecanoyl-*sn*-glycero-3-phosphocholine (**5**) pseudomolecular ion at *m/z* 496.3400 in standard (top) and coral extract (bottom) 15

Figure S13. Compared collision-induced dissociation of 1-*O*-hexadecanoyl-*sn*-glycero-3-phosphocholine (**5**) pseudomolecular ion at *m/z* 496.3400 in standard (top) and coral extract (bottom) 16

Figure S14. Collision-induced dissociation of sodiated **5**.. 16

Figure S15. Extracted ion chromatograms for 1-*O*-hexadecyl-*sn*-glycero-3-phosphocholine (**6**) pseudomolecular ion at *m/z* 482.3605 in standard (top) and coral extract (bottom) 17

Figure S16. Compared collision-induced dissociation of 1-*O*-hexadecyl-*sn*-glycero-3-phosphocholine (**6**) pseudomolecular ion at *m/z* 482.3605 in standard (top) and coral extract (bottom) 17

Figure S17. Extracted ion chromatograms for 1-*O*-octadecanoyl-*sn*-glycero-3-phosphocholine (**7**) pseudomolecular ion at *m/z* 524.3710 in standard (top) and coral extract (bottom)....... 18

Figure S18. Compared collision-induced dissociation of 1-*O*-octadecanoyl-*sn*-glycero-3-phosphocholine (**7**) pseudomolecular ion at *m/z* 524.3710 in standard (top) and coral extract (bottom) 18

Figure S19. ESI$^+$-HRMS spectrum of 1-*O*-arachidonoyl-*sn*-glycero-3-phosphoethanolamine (**8**) in coral extract... 19

Figure S20. Collision-induced dissociation of 1-*O*-arachidonoyl-*sn*-glycero-3-phosphoethanolamine (**8**) pseudomolecular ion at *m/z* 502.2929 in coral extract................ 19

Figure S21. Extracted ion chromatograms for 1-*O*-octadecanoyl-*sn*-glycero-3-phosphoethanolamine (**9**) pseudomolecular ion at *m/z* 482.3242 in standard (top) and coral extract (bottom)... 20

Figure S22. Compared collision-induced dissociation of 1-*O*-octadecanoyl-*sn*-glycero-3-phosphoethanolamine (**9**) pseudomolecular ion at *m/z* 482.3242 in standard (top) and coral extract (bottom)... 20

Figure S23. ESI$^+$-HRMS spectrum of 1-*O*-hexadec-1′-enyl-*sn*-glycero-3-phosphoethanolamine (**10**) in coral extract 21

Figure S24. Collision-induced dissociation of 1-*O*-hexadec-1′-enyl-*sn*-glycero-3-phosphoethanolamine (**10**) pseudomolecular ion at *m/z* 438.2979 in coral extract.............. 21

Figure S25. Extracted ion chromatograms for 1-*O*-(*Z*)-octadec-1′-enyl-*sn*-glycero-3-phosphoethanolamine (**11**) pseudomolecular ion at *m/z* 466.3292 in standard (top) and coral extract (bottom)... 22

Figure S26. Compared collision-induced dissociation of 1-*O*-(*Z*)-octadec-1′-enyl-*sn*-glycero-3-phosphoethanolamine (**11**) pseudomolecular ion at *m/z* 466.3292 in standard (top) and coral extract (bottom) 22

Figure S27. MS spectrum of compound **12** 23

Figure S28. Expanded MS spectrum of compound **12** 23

Figure S29. Collision-induced dissociation of parent ion at *m/z* 481.2925 [(**12**+Na)$^+$] 23

Figure S30. MS spectrum of compound **13** 23

Figure S31. MS spectrum of compound **14** 24

Figure S32. Expanded MS spectrum of compound **14** 24

Figure S33. Collision-induced dissociation of compound **14** pseudomolecular ion at *m/z* 429.3360 24

Figure S34. Collision-induced dissociation of compound **14** fragment ion at *m/z* 395.3306 ([M-H_2O_2+H]$^+$) 25

Figure S35. ^{1}H-NMR spectrum of **14** in $CDCl_3$ (500 MHz) 25

Figure S36. ^{13}C-NMR spectrum of **14** in $CDCl_3$ (125 MHz) 26

Figure S37. COSY spectrum of **14** in $CDCl_3$ 26

Figure S38. HSQC spectrum of **14** in $CDCl_3$ 27

Figure S39. HMBC spectrum of **14** in $CDCl_3$ 27

Figure S40. MS spectrum of compound **15** 28

Figure S41. Expanded MS spectrum of compound **15** 28

Figure S42. Collision-induced dissociation of compound **15** pseudomolecular ion at *m/z* 429.3360 28

Figure S43. Collision-induced dissociation of compound **15** fragment ion at *m/z* 395.3307 ([M-H_2O_2+H]$^+$) 29

Figure S44. MS spectrum of compound **16** 29

Figure S45. Expanded MS spectrum of compound **16** 29

Figure S46. Collision-induced dissociation of compound **16** fragment ion at *m/z* 409.3464 ([M-H_2O_2+H]$^+$) 30

Figure S47. MS spectrum of compound **17** 30

Figure S48. Collision-induced dissociation of compound **17** fragment ion at *m/z* 377.3200 (undefined fragment) 30

Figure S49. MS spectrum of compound **18** 31

Figure S50. Collision-induced dissociation of compound **18** fragment ion at *m/z* 562.3738 ([M+H]$^+$) 31

Figure S51. MS spectrum of compound **19** (t_R 13.69 min, exp. *m/z* 723.5011 corresponds to the molecular formula $C_{39}H_{72}O_{10}Na^+$, th. *m/z* 723.5018, 4 unsaturations). On the right, structure of compound **19** 32

Figure S52. Collision-induced dissociation of compound **19** sodiated molecular ion at *m/z* 723.5011 ([M+Na]$^+$). On the right, key fragmentation products allowing identification of compound **19** 32

Figure S53. MS spectrum of compound **20** (t_R 13.64 min, exp. *m/z* 773.5173 corresponds to the molecular formula $C_{43}H_{74}O_{10}Na^+$, th. *m/z* 773.5174, 7 unsaturations). On the right, structure of compound **20** 33

Figure S54. Collision-induced dissociation of compound **20** sodiated molecular ion at *m/z* 773.5173 ([M+Na]$^+$). On the right, key fragmentation products allowing identification of compound **20** 33

Figure S55. MS spectrum of compound **21** (t_R 13.42 min, exp. *m/z* 773.5171 corresponds to the molecular formula $C_{43}H_{74}O_{10}Na^+$, th. *m/z* 773.5174, 7 unsaturations). On the right, structure of compound **21** 34

Figure S56. Collision-induced dissociation of compound **21** sodiated molecular ion at *m/z* 773.5173 ([M+Na]$^+$). On the right, key fragmentation products allowing identification of compound **21** 34

Figure S57. MS spectrum of compound **22** (t_R 13.16 min, exp. *m/z* 847.5324 corresponds to the molecular formula $C_{49}H_{76}O_{10}Na^+$, th. *m/z* 773.5331, 12 unsaturations). On the right, structure of compound **22** 35

Figure S58. Collision-induced dissociation of compound **22** sodiated molecular ion at *m/z* 847.5325 ([M+Na]$^+$). On the right, key fragmentation products allowing identification of compound **22** 35

Figure S59. MS spectrum of compound **23** (t_R 12.69 min, exp. *m/z* 774.5491 corresponds to the molecular formula $C_{43}H_{77}NO_9Na^+$, th. *m/z* 774.5490, 6 unsaturations). On the right, structure of compound **23** 36

Figure S60. Collision-induced dissociation of compound **23** sodiated molecular ion at *m/z* 774.5490 ([M+Na]$^+$). Below, key fragmentation products allowing identification of compound **23** 36

Figure S61. Picture of coral nubbins exposed at 1 mg/L homosalate (right) compared to control (left) 37

Table S1. Results of reciprocal *blastp* searches were also conducted using the web-based tool against the uniprot/Swiss-Prot, and model organism (landmark) databases, and is some specific cases, the conserved domains (CDD) search. In blue are proteins coded by the *P. damicornis* genome. In grey are results highlighted in the main body of the manuscript 38

Experimental Section

***Pocillopora damicornis*.** Fragments of the coral *P. damicornis* were collected in Oman in 2014 (CITES permit 37/2014). This procedure had no impact on the wild population. The coral was acclimated in tanks at the Banyuls Oceanological Observatory. New colonies were obtained in the laboratory from the fragments and used for our experiments. The corals were maintained in artificial sea water (ASW) prepared with reverse osmosis purified water and Reef Salt SeaChem salts. Salinity was adjusted to 36 g/L, pH = 8, and the temperature was set at 24 °C. All experiments were conducted with the same ASW.

Preparation of coral for exposition to UV filters. Coral pieces (1-1.5 cm) were cut from branch tips from the same mother colony. Each piece was attached to a fishing line, and the coral pieces were then suspended on an ~ 5-6 cm laminated iron wire, making sure the lines had the appropriate length so that the coral pieces did not touch the beaker edge or bottom during the assay. Each suspension of 5 coral pieces (5 replicates) was clamped to a line above the aquarium of origin, thereby immerging the pieces. The suspended pieces were acclimatized for approximately 1 month before the assay to allow for full healing.

Exposure setup. Beakers (250 mL) were filled with ASW (200 mL). UV filters were dissolved in DMSO at concentrations of 400, 120, 20 and 2 µg/mL. Each of these solutions (500 µl) was added into separate beakers, resulting in final concentrations of 1000, 300, 50, and 5 µg/L. DMSO (500 µL) was used as a negative control, and the DMSO concentration was 0.25 % v/v in all beakers. The suspensions of 5 coral pieces were dipped into the beakers, with the wire positioning the pieces halfway into the water. Gentle bubbling in the beakers was maintained using aquarium air pumps equipped with a Teflon tubing. Homogeneous lighting was set to a photosynthetic photon flux density of 250 µmol/m^2/s, with 10 h of day and 14 h of night per day. The corals were transferred every 24 h to a new beaker prepared in the same way. The total exposure lasted for 7 days. Pictures were taken every 24 h to monitor coral polyps. Coral exposure at 1000 µg/L ES, HS, BEMT, and BM was repeated in separate experiments to confirm the results.

Extraction. After 7 days, the coral pieces were collected, and the fishing lines were removed with a scalpel. Each coral piece was placed into a 20-mL test tube, covered with MeOH/acetonitrile 1/1, and sonicated for 20 min. Once the polyps were dissolved, the solvent was collected by filtration, evaporated and kept at – 80 °C until analysis. All solvents used for extraction and profiling were LC-MS gradient grade (Biosolve, Dieuze, France).

UHPLC-HRMS profiling. High-resolution MS/MS analyses were conducted with a Thermo UHPLC-HRMS system. Analyses were performed in the electrospray positive ionization mode in the range of 133.4–2000 Da in centroid mode. The mass detector was an Orbitrap MS/MS FT Q-Exactive focus mass spectrometer. The analyses were conducted in FullMS data dependent MS^2 mode. In FullMS, the resolution was set to 70,000, and the AGC target was 3.10^6. In MS^2, the resolution was 17,500, AGC target 10^5, isolation window 0.4 Da, and stepped normalized collision energy 15/30/45 was used, with 15 s dynamic exclusion. The lock mass option was set for ion at m/z 144.98215, corresponding to $Cu(CH_3CN)_2^+$. The UHPLC column was a Phenomenex Luna Omega polar C-18 150 × 2.1 mm, 1.6 µm. The column temperature was set to 42 °C, and the flow rate was 0.5 mL.min^{-1}. The solvent

system was a mixture of water (solution A) with increasing proportions of acetonitrile (solution B), both solvents modified with 0.1 % formic acid. The gradient was as follows: 2 % B 3 min before injection, then from 1 to 13 min, a shark fin gradient increase of B up to 100 % (curve 2), followed by 100 % B for 5 min. The flow was diverted (not injected into the mass spectrometer) before injection, up to 1 min after injection. The coral extracts were dissolved in MeOH (1.5 mg/mL) by sonication, and 1 µL was injected onto the column.

Metabolomic analyses. Profiles were analyzed with Compound Discoverer® (CD) 2.1 (ThermoFisher, Villebon, France). Coral exposed to DMSO was used as the reference metabolome, and blank injections were used to remove irrelevant ion peaks (an ion peak is considered only if the signal is at least 5 times more intense than in the blank). An untargeted metabolomic workflow was used, with modifications. This workflow finds and identifies the differences between samples, performing retention time alignment, unknown compound detection, and compound grouping across all samples. The workflow predicts elemental compositions for all compounds, fills gaps across all samples, hides chemical background (using Blank samples); identifies compounds using mzCloud (ddMS2) and ChemSpider (formula or exact mass); performs similarity searches for all compounds with ddMS2 data using mzCloud; maps compounds to biological pathways using KEGG database; and calculates a differential analysis (*t*-test or ANOVA) and determines *p*-values, adjusted *p*-values, ratios, fold change.

The retention time window was set to 2–17 min. The maximum shift for alignment was 0.1 min, the maximum mass tolerance was 3 ppm, and the minimum peak intensity was 2.10^6. Volcano plot (Figure S1) was drawn with CD using centered and scaled data. The integration data from CD were used to construct figures 1 and 4 with GraphPad Prism 5.

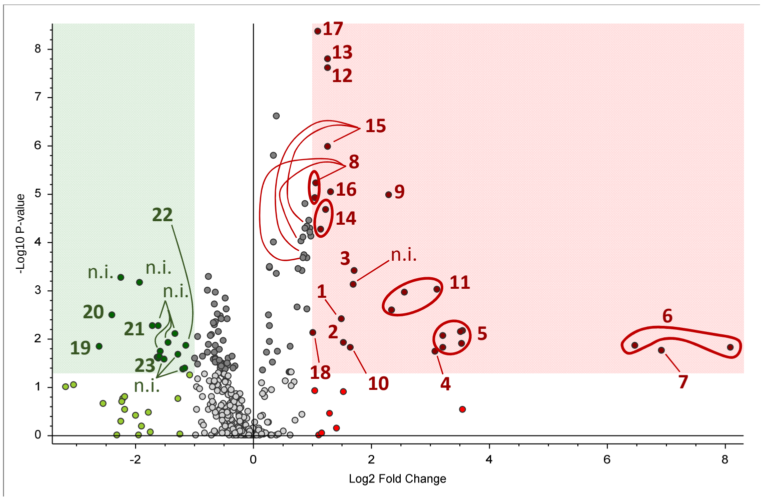

Figure S1. Annotated volcano plot comparing the exposition of coral to ES at 1 mg/L to the control DMSO-exposed corals. Note that several ions (dots) can correspond to the same molecule. The red box frames the ions the concentration of which significantly increased upon exposition to ES. The horizontal limit of the box is for a p value of 0.05: all ions above this line are significantly up-regulated. The vertical limit of the box is for a Log2 fold changes of +1. The green box frames ions corresponding to down-regulated metabolites. Ions dot numbering refers to Table 1. n.i. is for 'not identified' (very small peaks or analytical noise).

Additional experimental section: the search for PAF in coral profiles

The retention times of PAF and its isomer (compound **7**) were rather close using the solvent gradient described in the article and the discovery mode dd2 does not guarantee that a part of the peak could not be PAF instead of **7**. Therefore, additional analyses were conducted in FullMS + PRM mode using the same column, column temperature and flow rate. In FullMS, the settings were the same as previously. In MS^2 (PRM mode), the resolution was 17,500, AGC target 10^5, and isolation window 0.4 Da. The ion at *m/z* 524.3711 was fragmented at two normalized collision energy of 30 and 45 %, from 1 to 19 min. The solvent system was a mixture of water (solution A) with increasing proportions of acetonitrile (solution B), both solvents modified with 0.1 % formic acid. The gradient was as follows: 50 % B 3 min before injection, then from 1 to 14 min, a linear gradient increase of B up to 65 %, from 14 to 15 min, a linear gradient increase of B up to 100 %, followed by 100 % B for 5 min. This experiment demonstrated unambiguously that PAF was not detectable in the coral profiles (Figure S2, Figure S3).

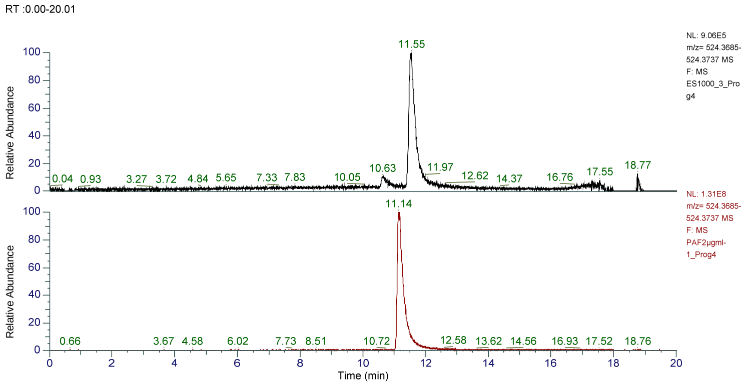

Figure S2. Compared extracted ion chromatograms at m/z 524.3711 for (top) the metabolomic profile of coral exposed to ES at 1000 µg/L, and (bottom) the PAF standard diluted at 2 µg/mL in MeOH.

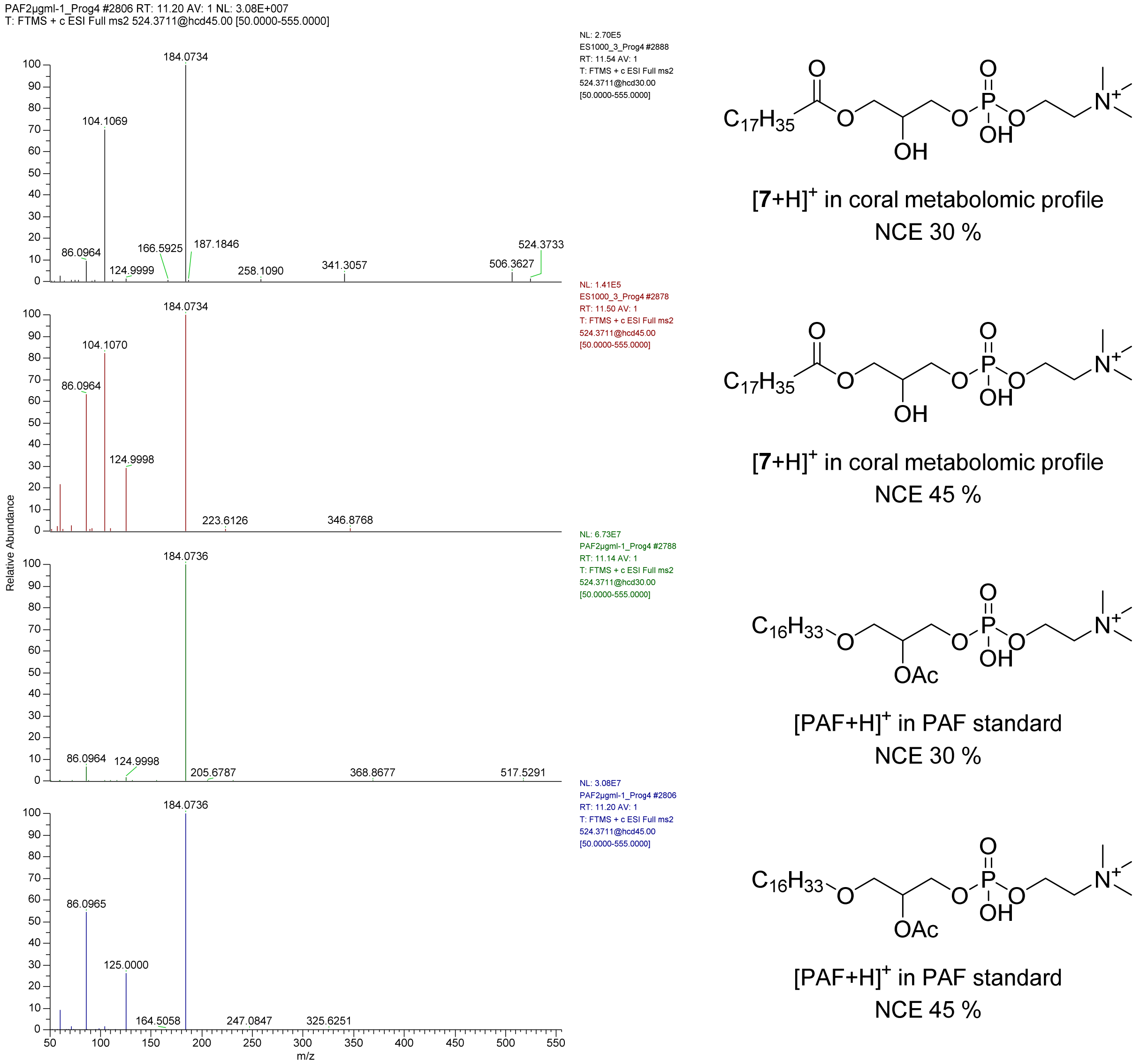

Figure S3. Compared MS^2 spectra of **7** and PAF pseudomolecular ions at Normalized Collision Energies of 30 and 45 %. The choline ion at theoretical *m/z* 104.1070 is absent or very small in $[PAF+H]^+$ MS^2 spectra.

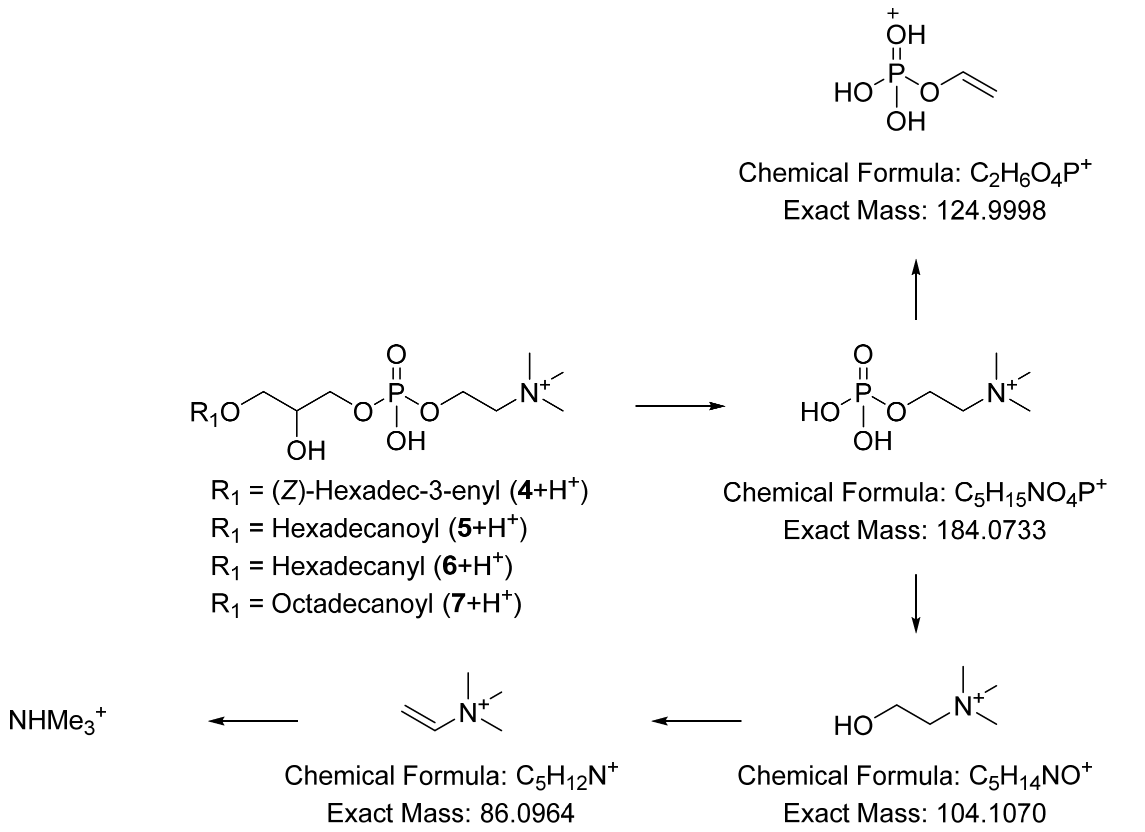

Scheme S1. Proposed fragmentation scheme for compounds **4**-**7** pseudomolecular ions

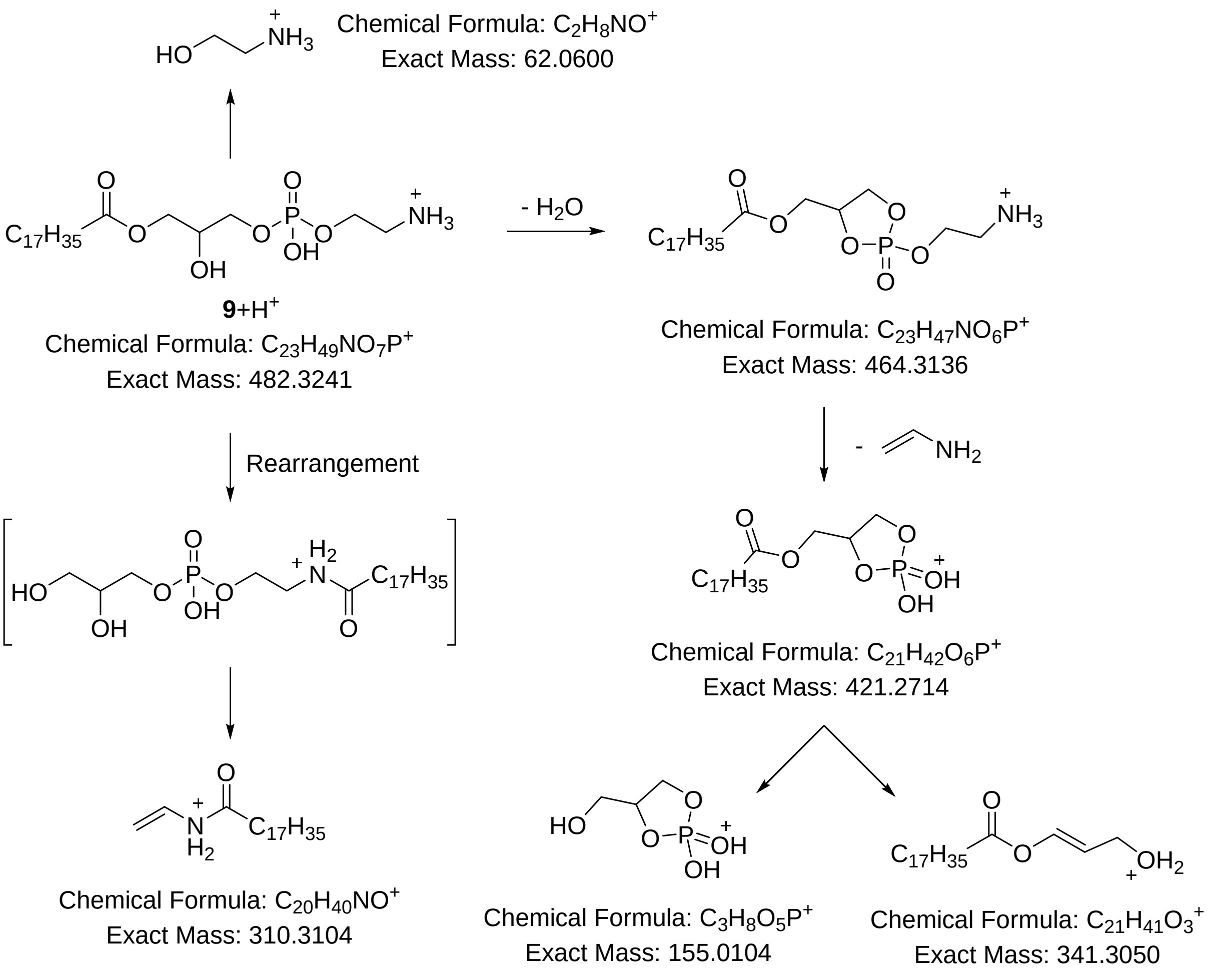

Scheme S2. Proposed fragmentation scheme for compound **9** pseudomolecular ion

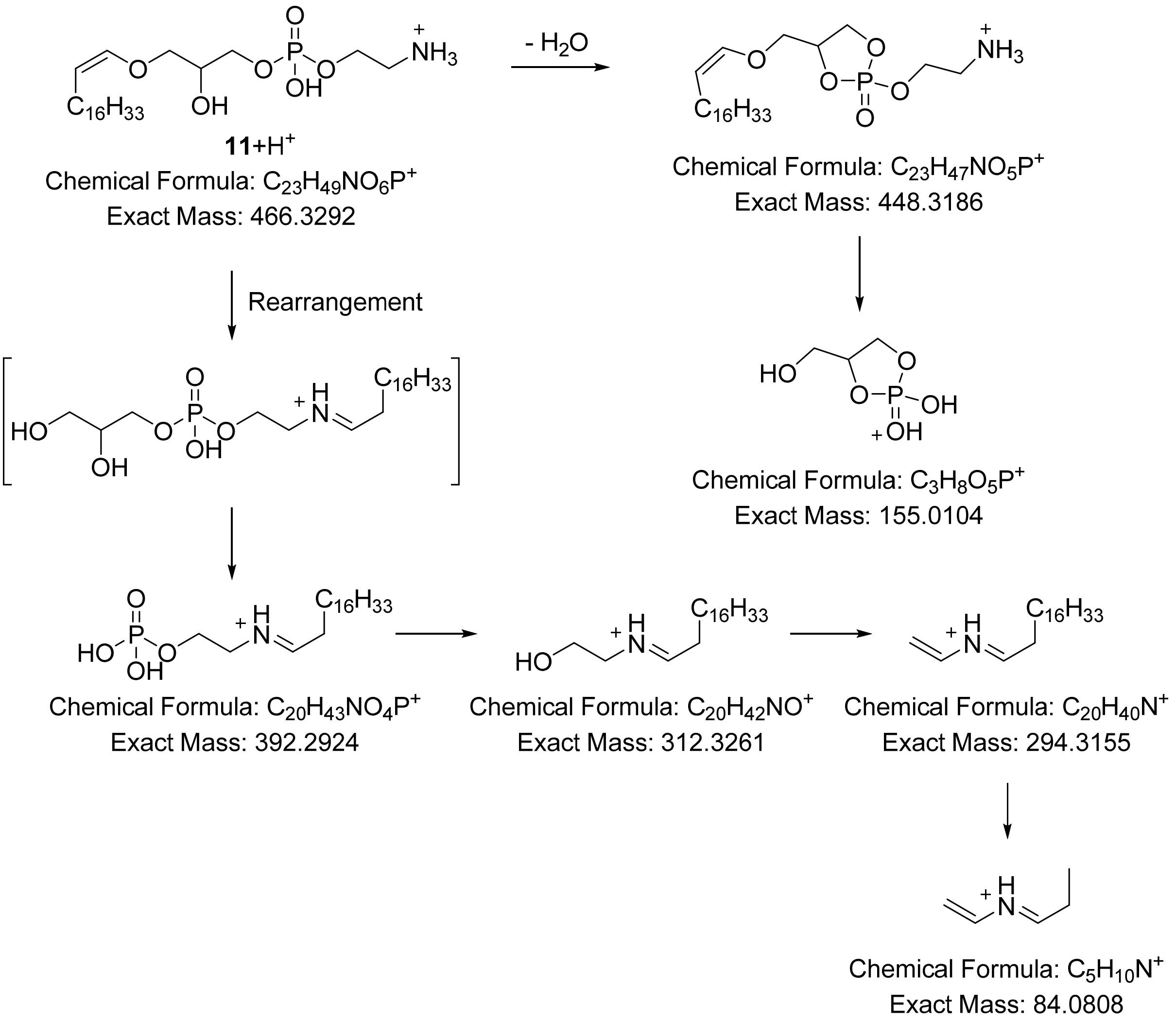

Scheme S3. Proposed fragmentation scheme for compound **11** pseudomolecular ion

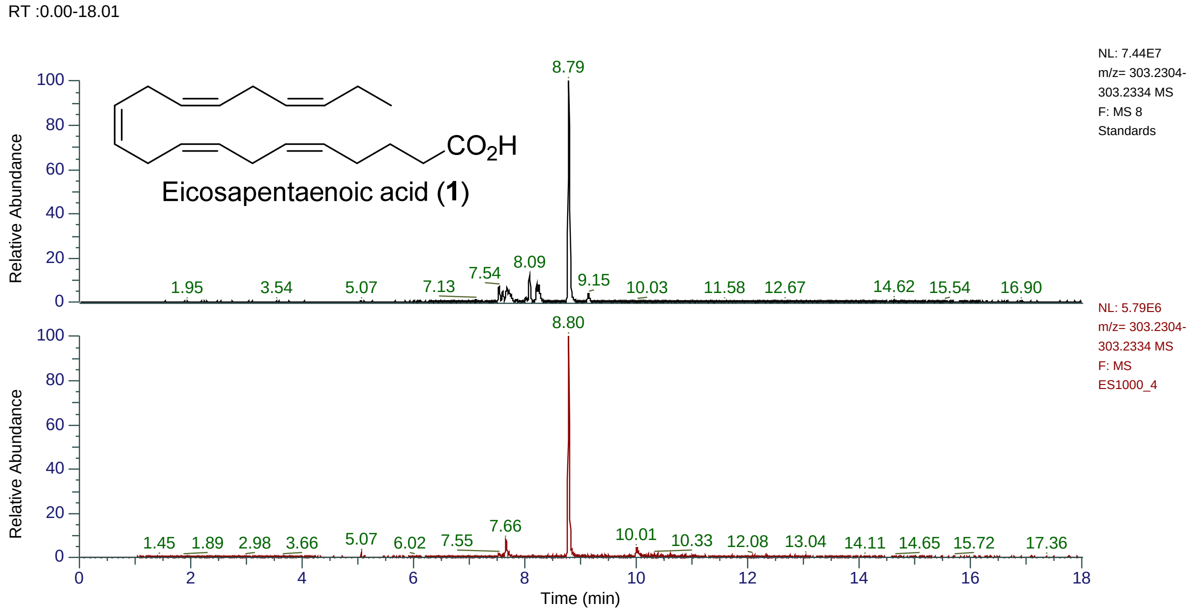

Figure S4. Extracted ion chromatograms for eicosapentaenoic acid (**1**) pseudomolecular ion at *m/z* 303.2319 in standard (top) and coral extract (bottom)

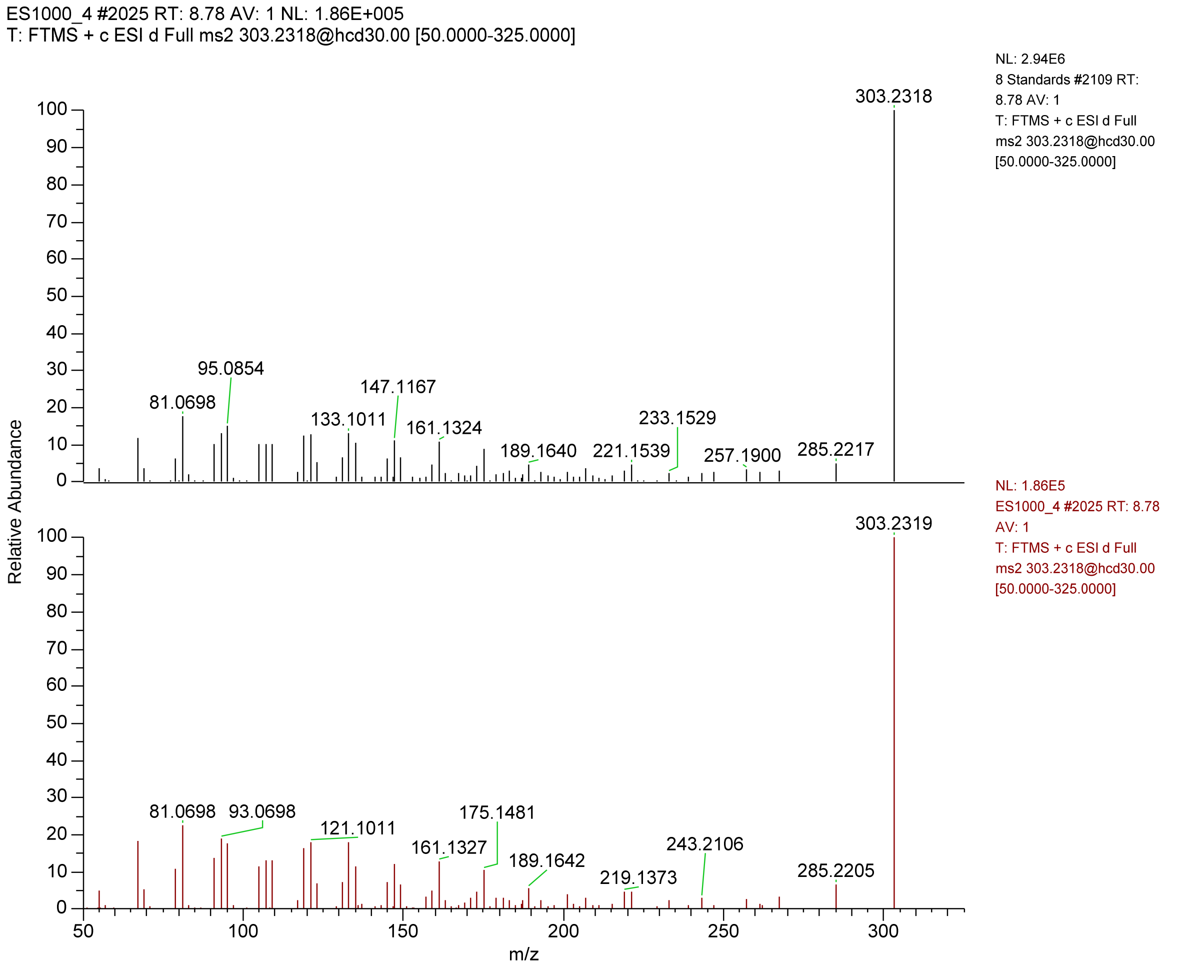

Figure S5. Compared collision-induced dissociation of eicosapentaenoic acid (**1**) pseudomolecular ion at *m/z* 303.2319 in standard (top) and coral extract (bottom)

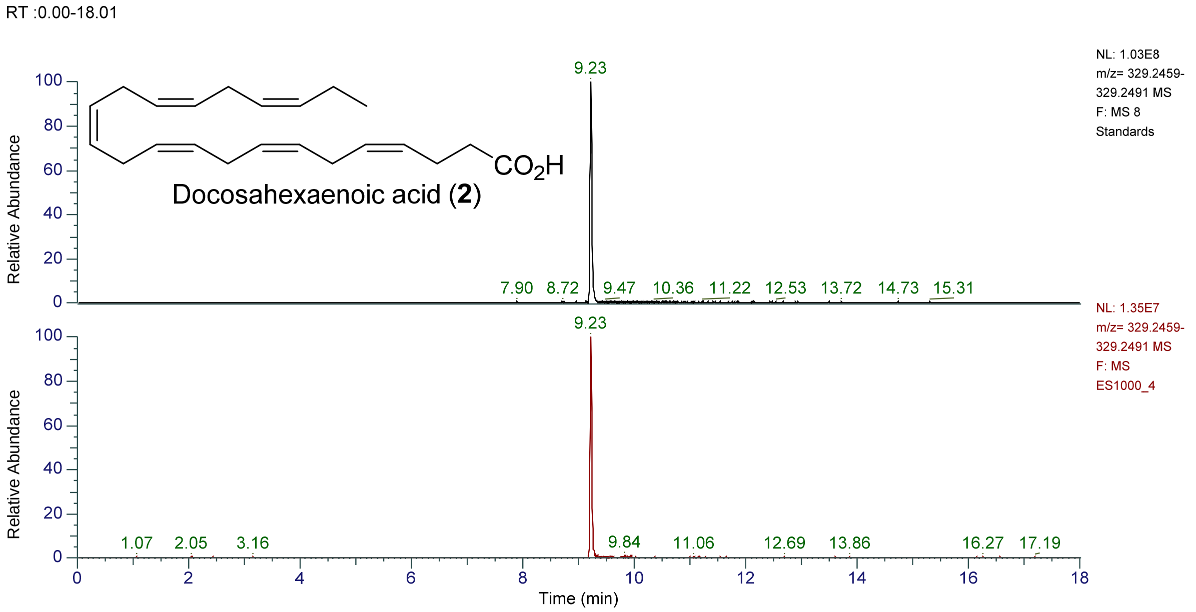

Figure S6. Extracted ion chromatograms for docosahexaenoic acid (**2**) pseudomolecular ion at *m/z* 329.2475 in standard (top) and coral extract (bottom)

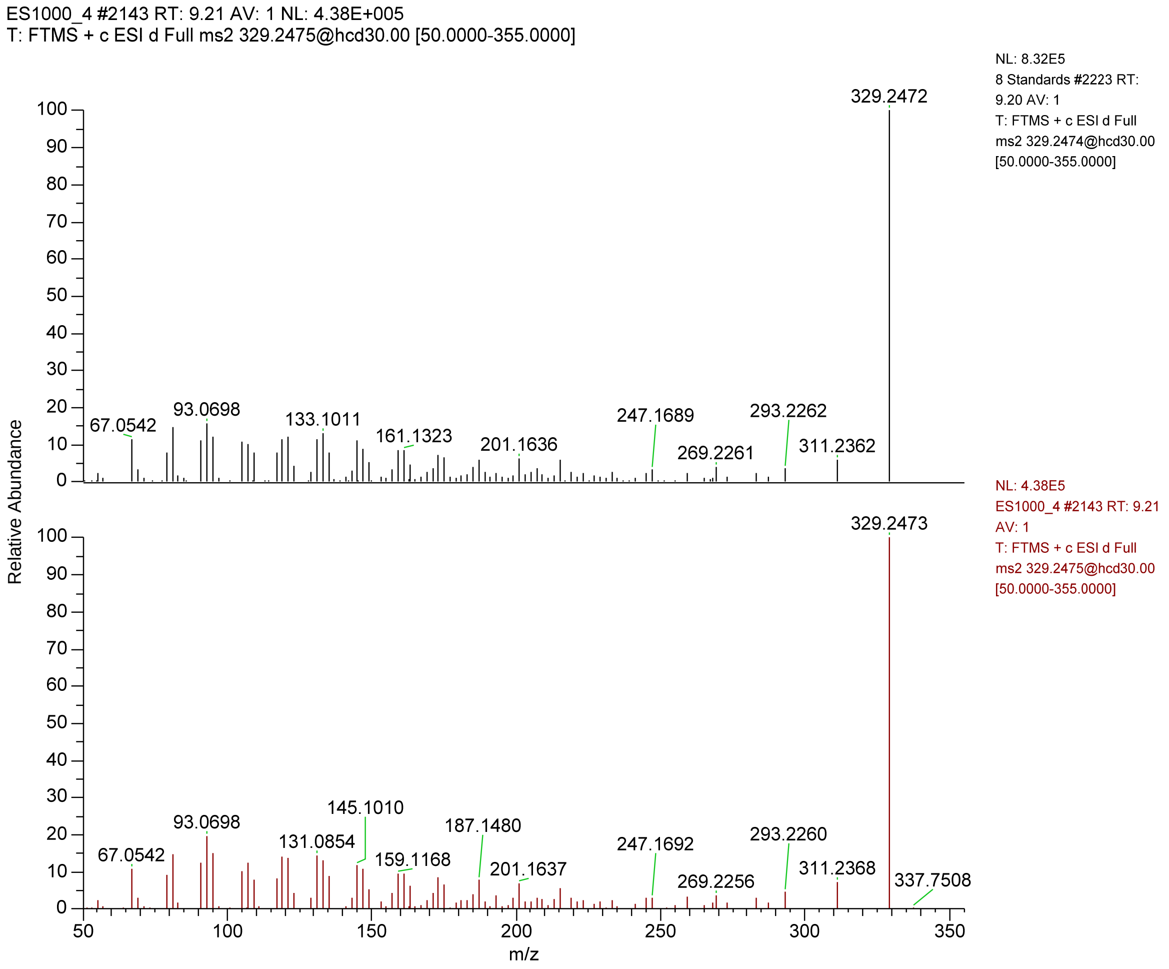

Figure S7. Compared collision-induced dissociation of docosahexaenoic acid (**2**) pseudomolecular ion at *m/z* 329.2475 in standard (top) and coral extract (bottom)

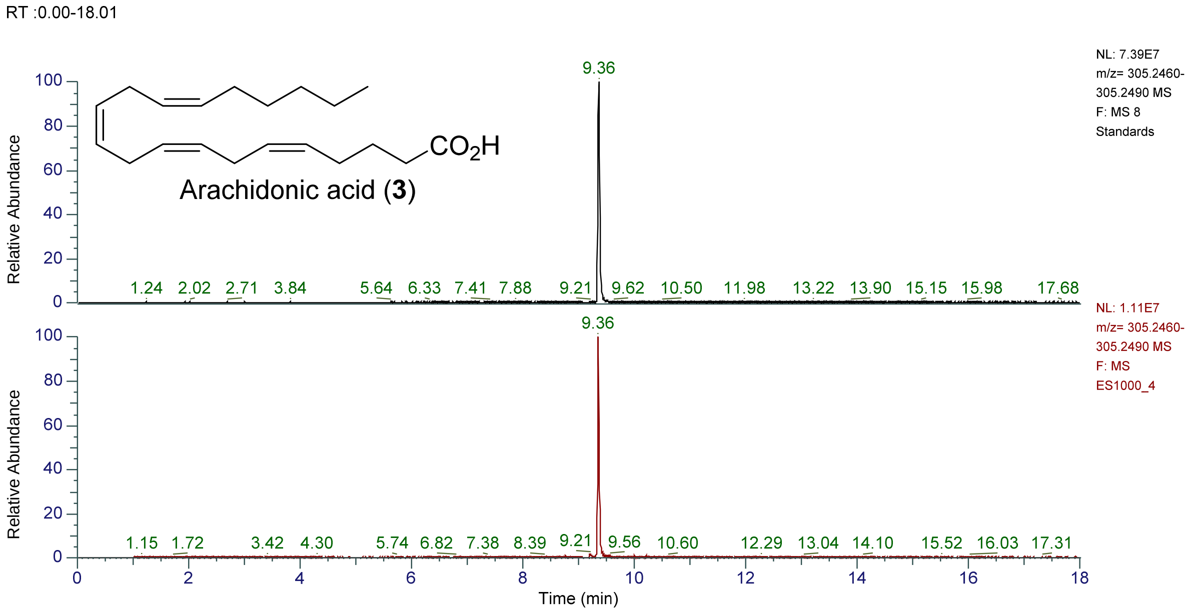

Figure S8. Extracted ion chromatograms for arachidonic acid (**3**) pseudomolecular ion at *m/z* 305.2475 in standard (top) and coral extract (bottom)

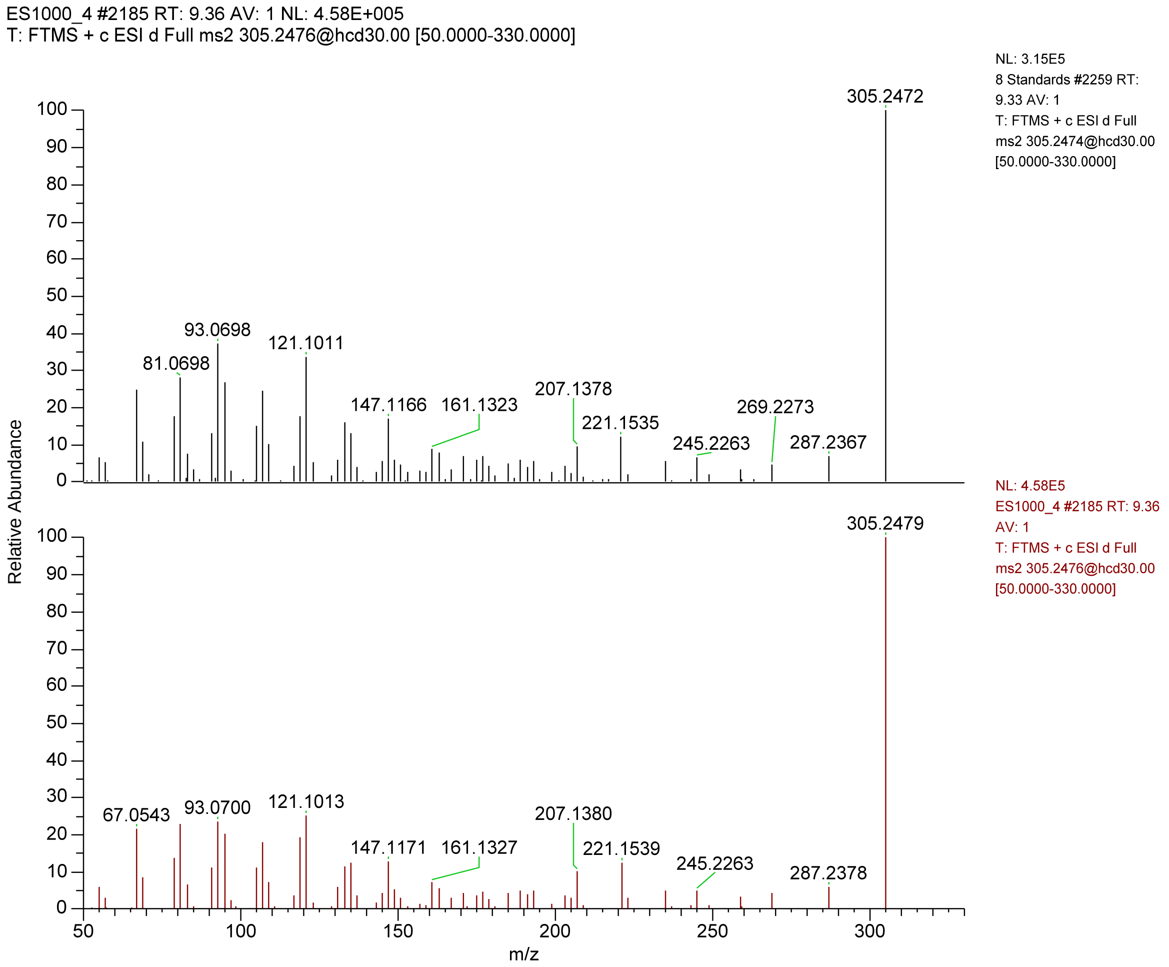

Figure S9. Compared collision-induced dissociation of arachidonic acid (**3**) pseudomolecular ion at *m/z* 305.2475 in standard (top) and coral extract (bottom)

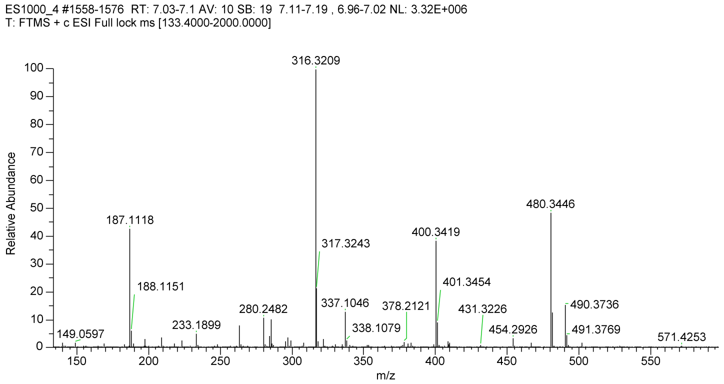

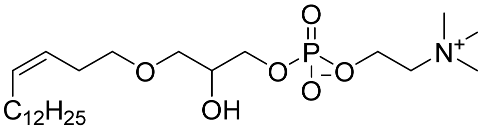

1-*O*-(3*Z*-hexadecenyl)-s*n*-glycero-3-phosphocholine (**4**)

Figure S10. ESI$^+$-HRMS spectrum of 1-*O*-(3*Z*-hexadecenyl)-*sn*-glycero-3-phosphocholine (**4**) in coral extract

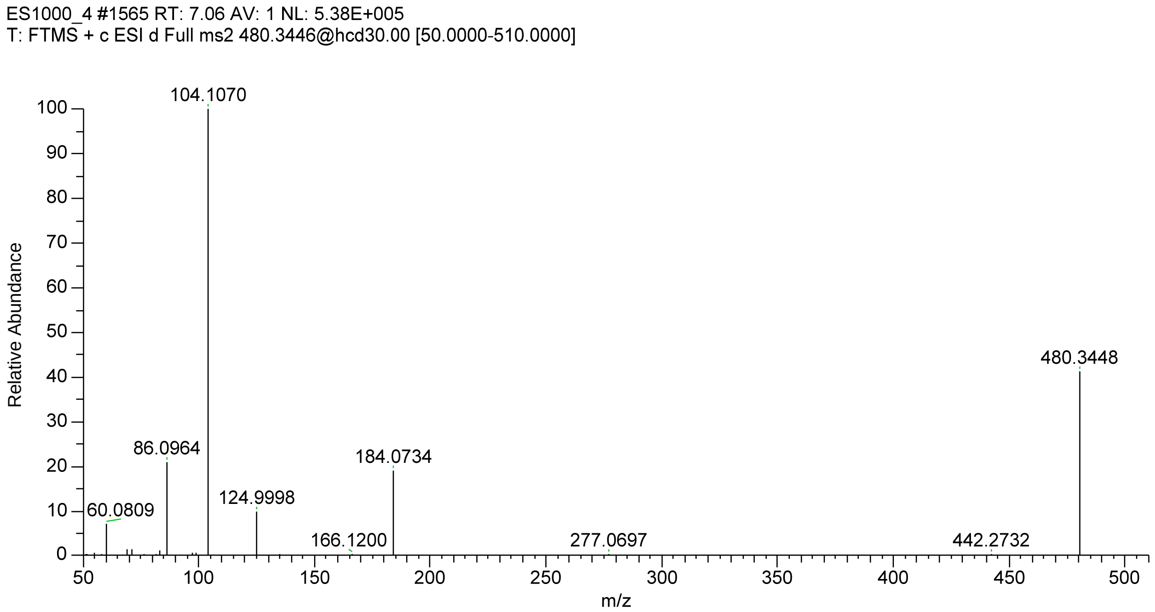

Figure S11. Collision-induced dissociation of 1-*O*-(3*Z*-hexadecenyl)-*sn*-glycero-3-phosphocholine (**4**) pseudomolecular ion at *m/z* 480.3446 in coral extract (bottom)

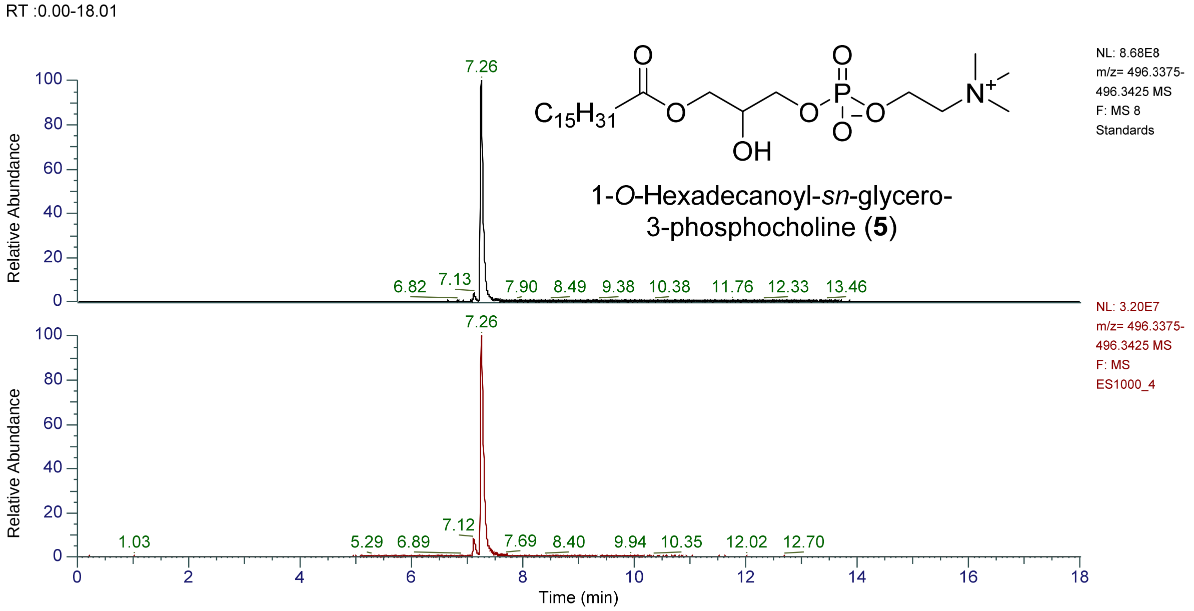

Figure S12. Extracted ion chromatograms for 1-*O*-hexadecanoyl-*sn*-glycero-3-phosphocholine (**5**) pseudomolecular ion at *m/z* 496.3400 in standard (top) and coral extract (bottom)

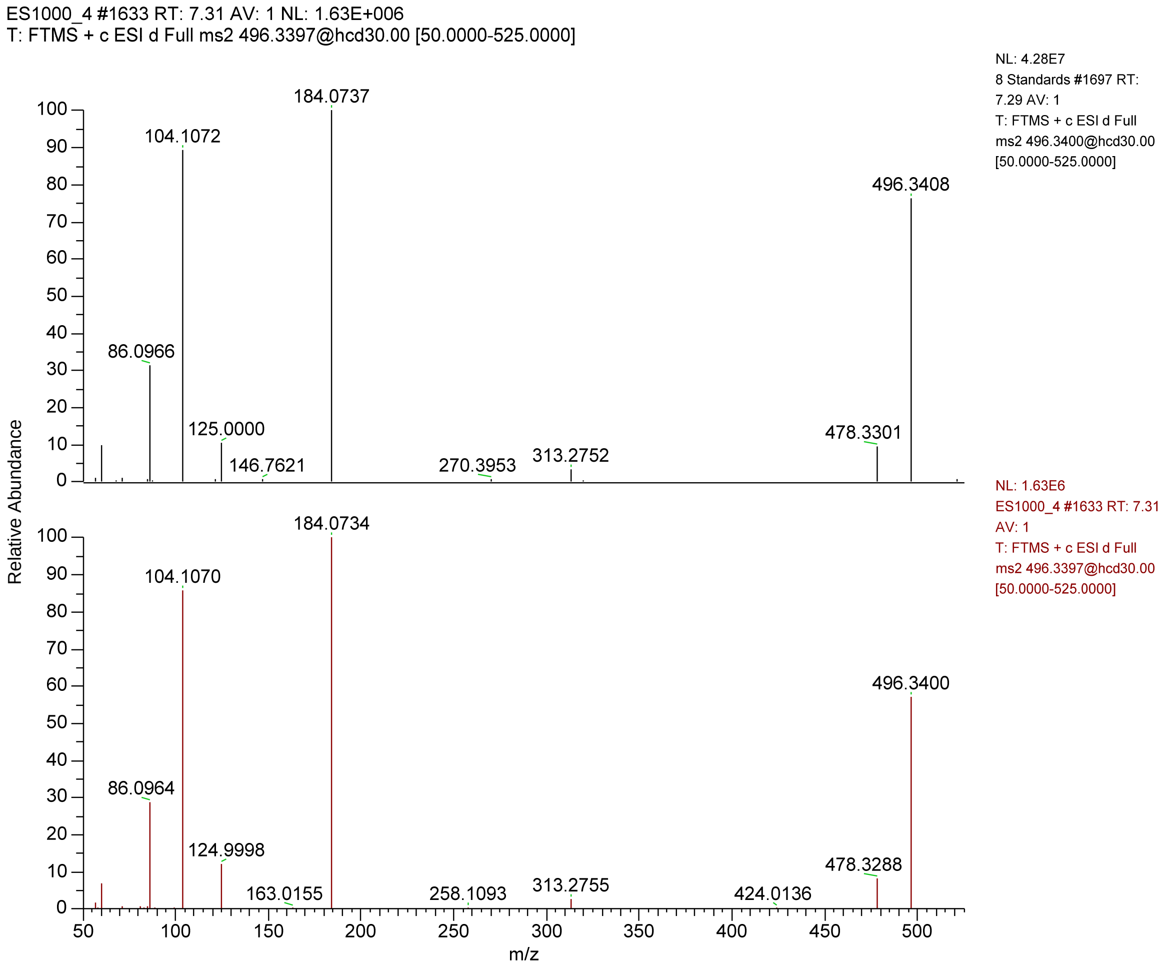

Figure S13. Compared collision-induced dissociation of 1-*O*-hexadecanoyl-*sn*-glycero-3-phosphocholine (**5**) pseudomolecular ion at *m/z* 496.3400 in standard (top) and coral extract (bottom)

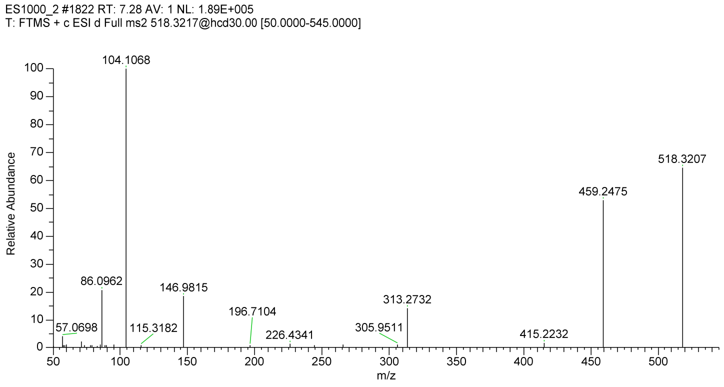

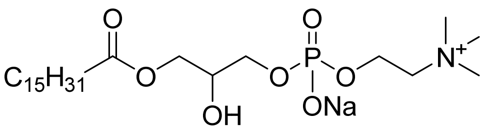

[**5**+Na]$^+$

Figure S14. Collision-induced dissociation of sodiated **5**

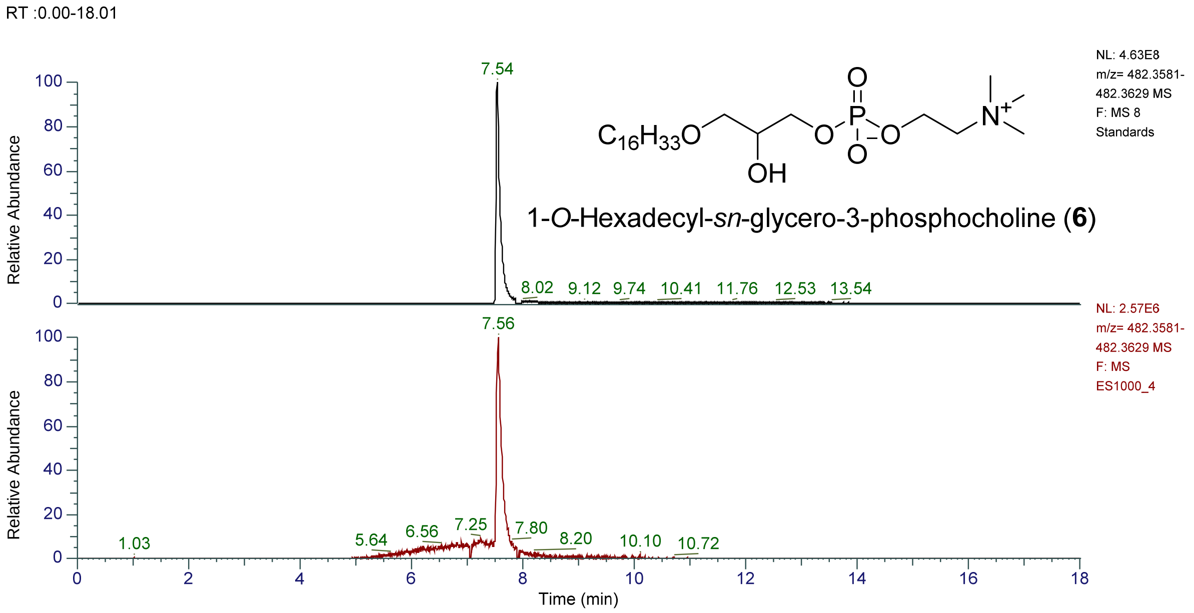

Figure S15. Extracted ion chromatograms for 1-*O*-hexadecyl-*sn*-glycero-3-phosphocholine (**6**) pseudomolecular ion at *m/z* 482.3605 in standard (top) and coral extract (bottom)

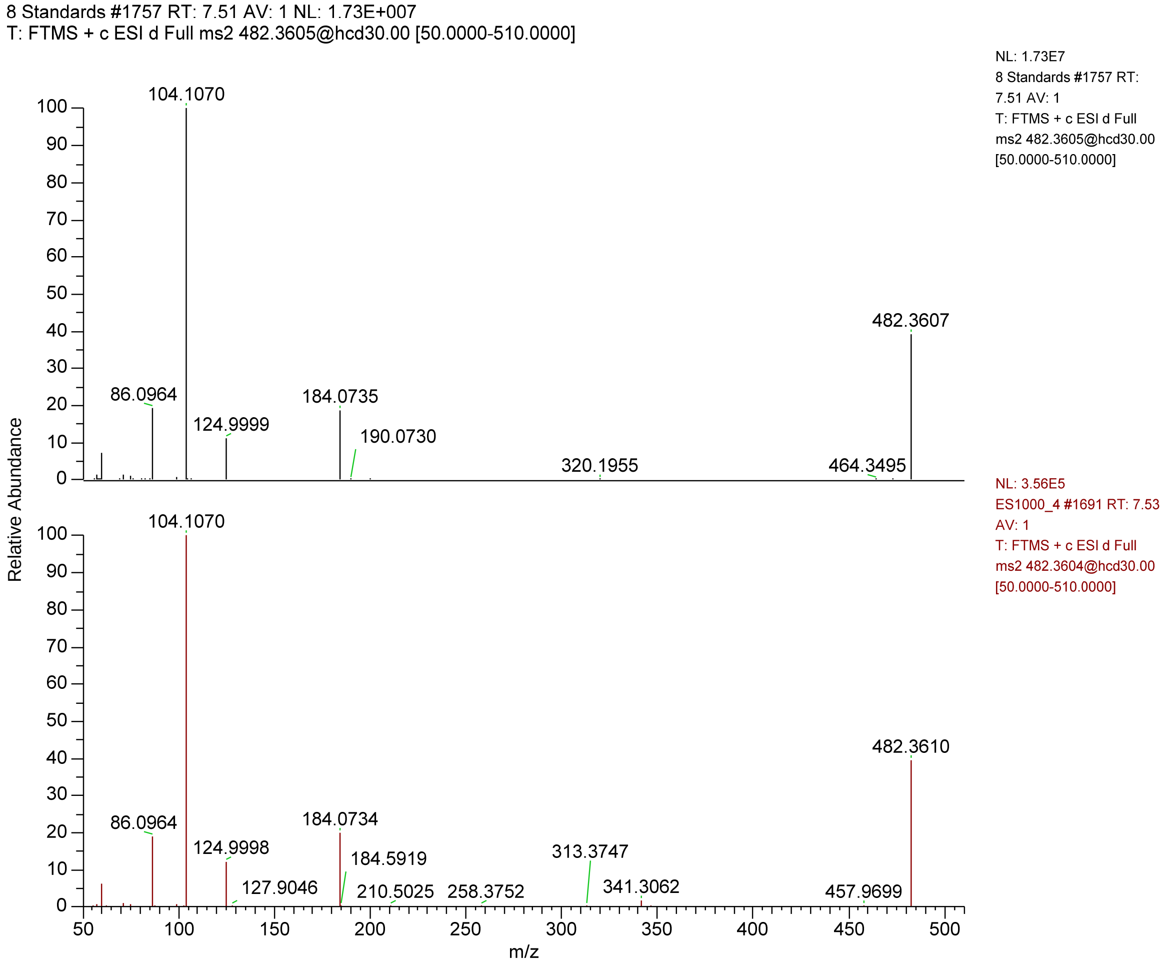

Figure S16. Compared collision-induced dissociation of 1-*O*-hexadecyl-*sn*-glycero-3-phosphocholine (**6**) pseudomolecular ion at *m/z* 482.3605 in standard (top) and coral extract (bottom)

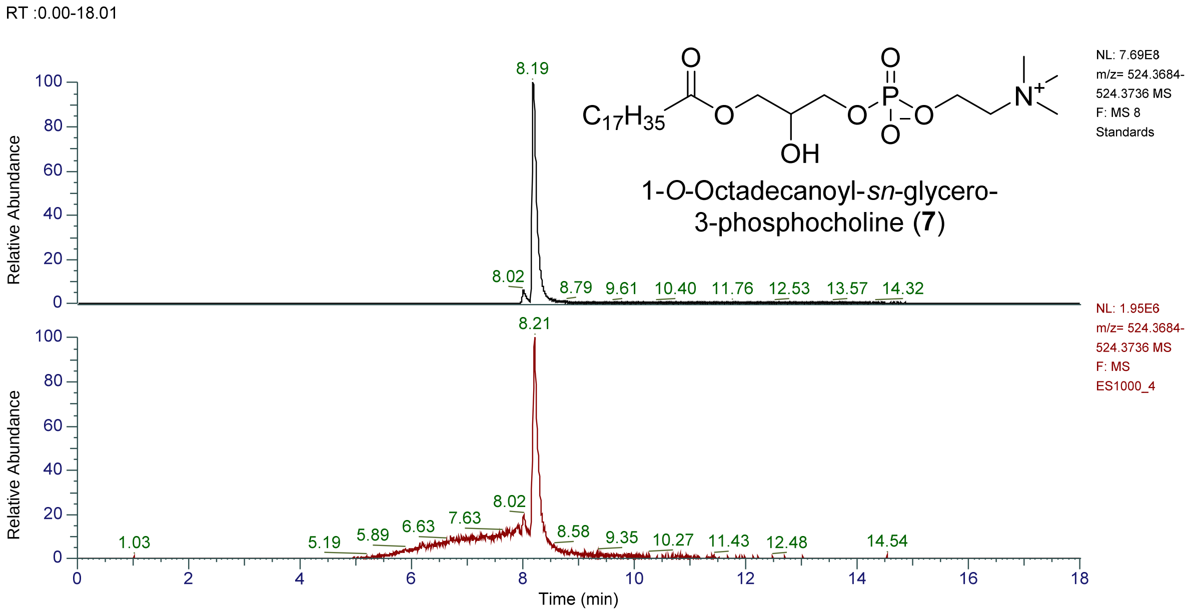

Figure S17. Extracted ion chromatograms for 1-*O*-octadecanoyl-*sn*-glycero-3-phosphocholine (**7**) pseudomolecular ion at *m/z* 524.3710 in standard (top) and coral extract (bottom)

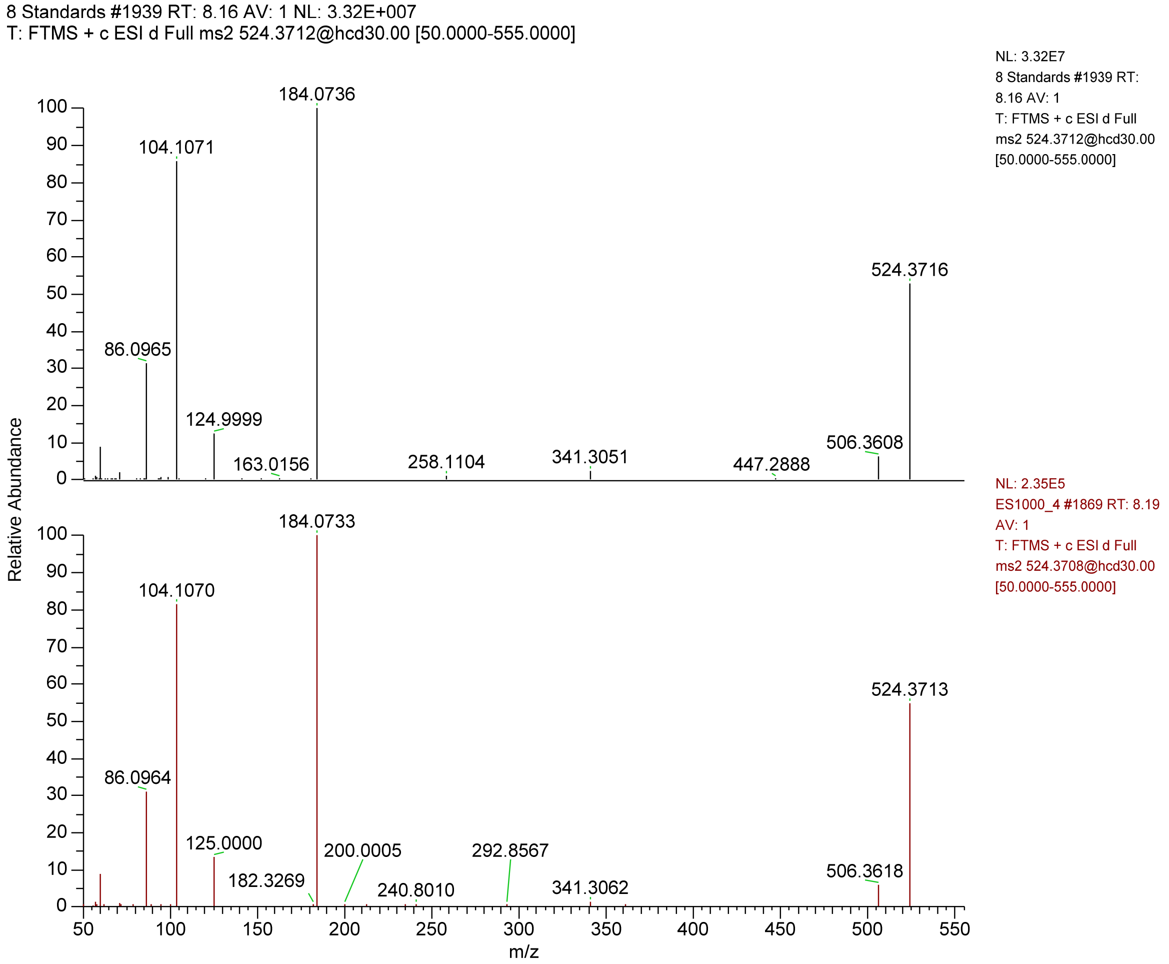

Figure S18. Compared collision-induced dissociation of 1-*O*-octadecanoyl-*sn*-glycero-3-phosphocholine (**7**) pseudomolecular ion at *m/z* 524.3710 in standard (top) and coral extract (bottom)

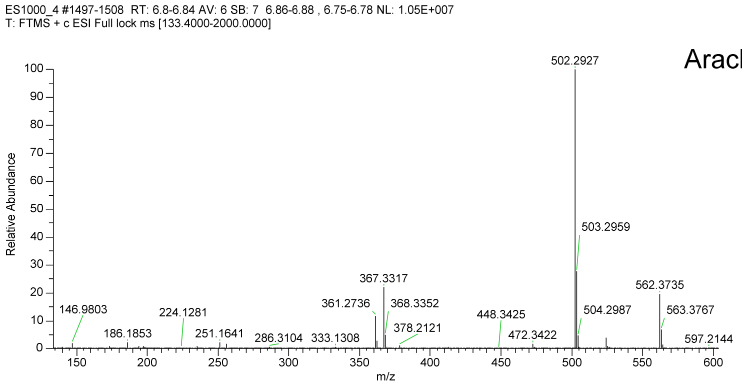

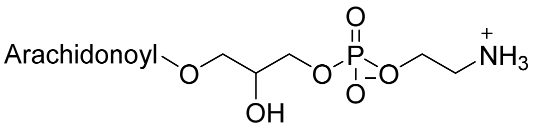

1-*O*-Arachidonyl-*sn*-glycero-
3-phosphoethanolamine (**8**)

Figure S19. ESI$^+$-HRMS spectrum of 1-*O*-arachidonoyl-*sn*-glycero-3-phosphoethanolamine (**8**) in coral extract

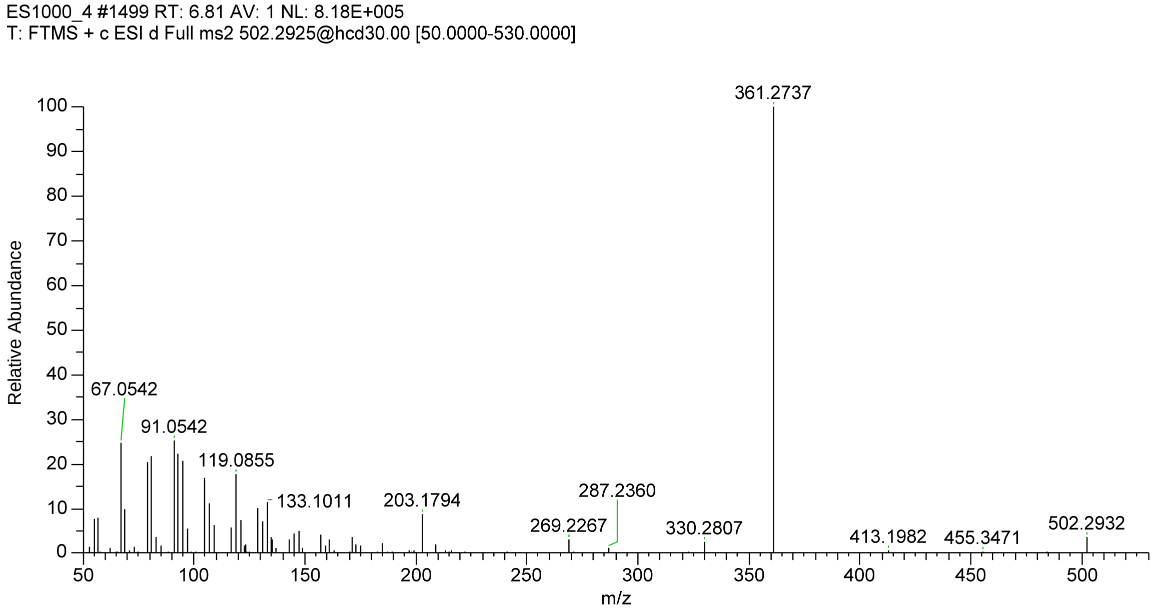

Figure S20. Collision-induced dissociation of 1-*O*-arachidonoyl-*sn*-glycero-3-phosphoethanolamine (**8**) pseudomolecular ion at *m/z* 502.2929 in coral extract

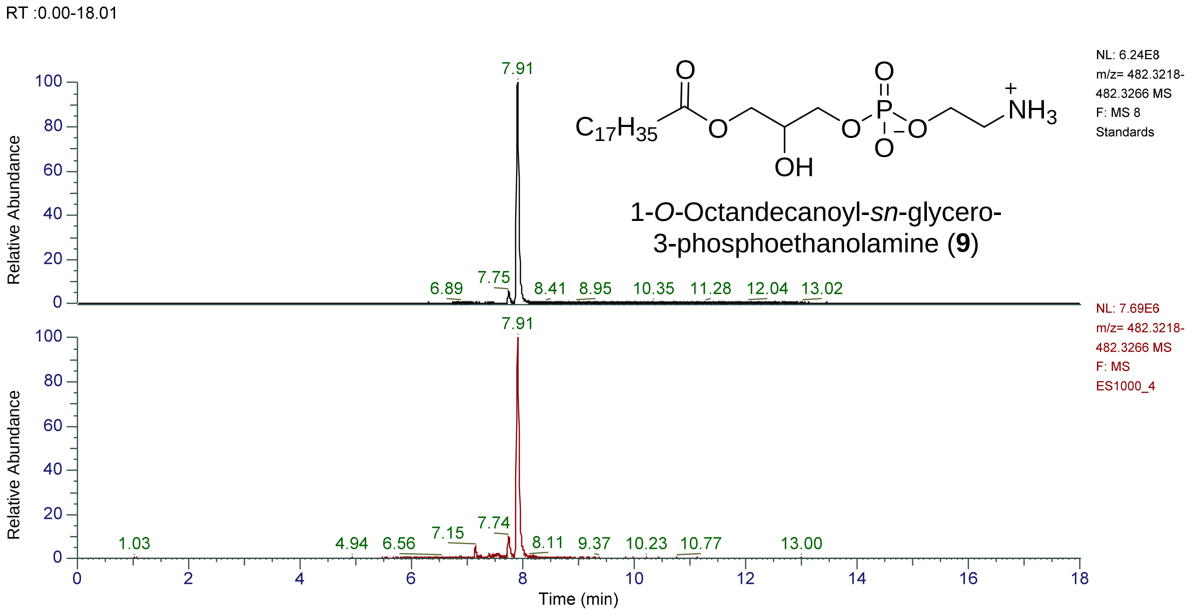

Figure S21. Extracted ion chromatograms for 1-*O*-octadecanoyl-*sn*-glycero-3-phosphoethanolamine (**9**) pseudomolecular ion at *m/z* 482.3242 in standard (top) and coral extract (bottom)

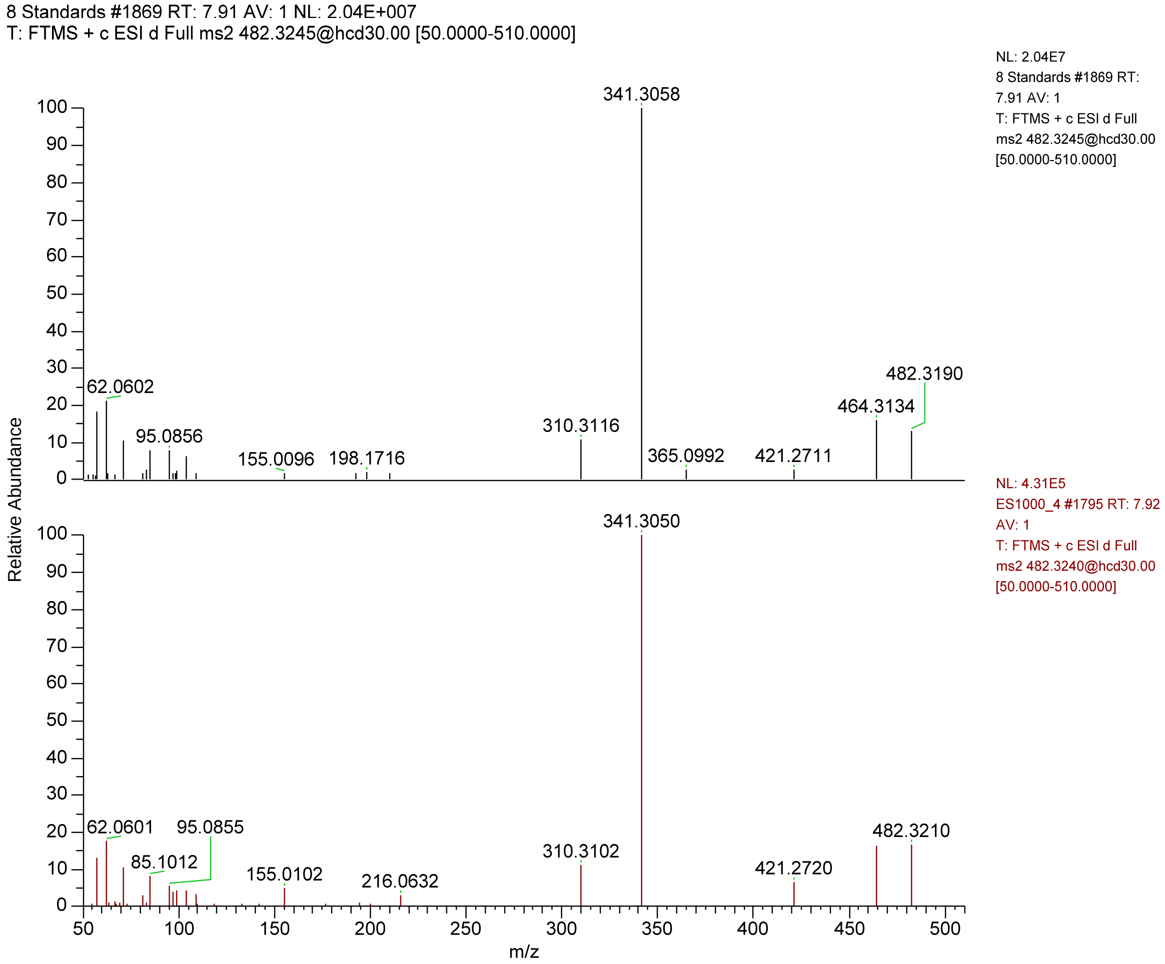

Figure S22. Compared collision-induced dissociation of 1-*O*-octadecanoyl-*sn*-glycero-3-phosphoethanolamine (**9**) pseudomolecular ion at *m/z* 482.3242 in standard (top) and coral extract (bottom)

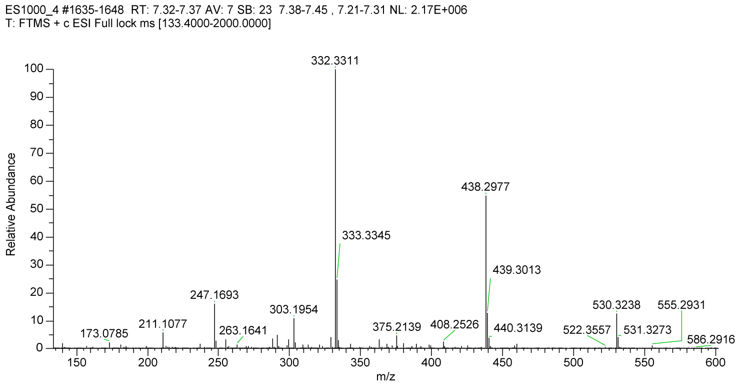

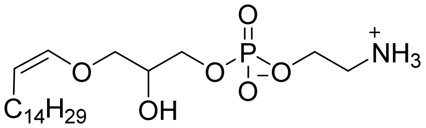

1-*O*-Hexadec-1-enyl-*sn*-glycero-3-phosphoethanolamine (**10**)

Figure S23. ESI^{+}-HRMS spectrum of 1-*O*-hexadec-1′-enyl-*sn*-glycero-3-phosphoethanolamine (**10**) in coral extract

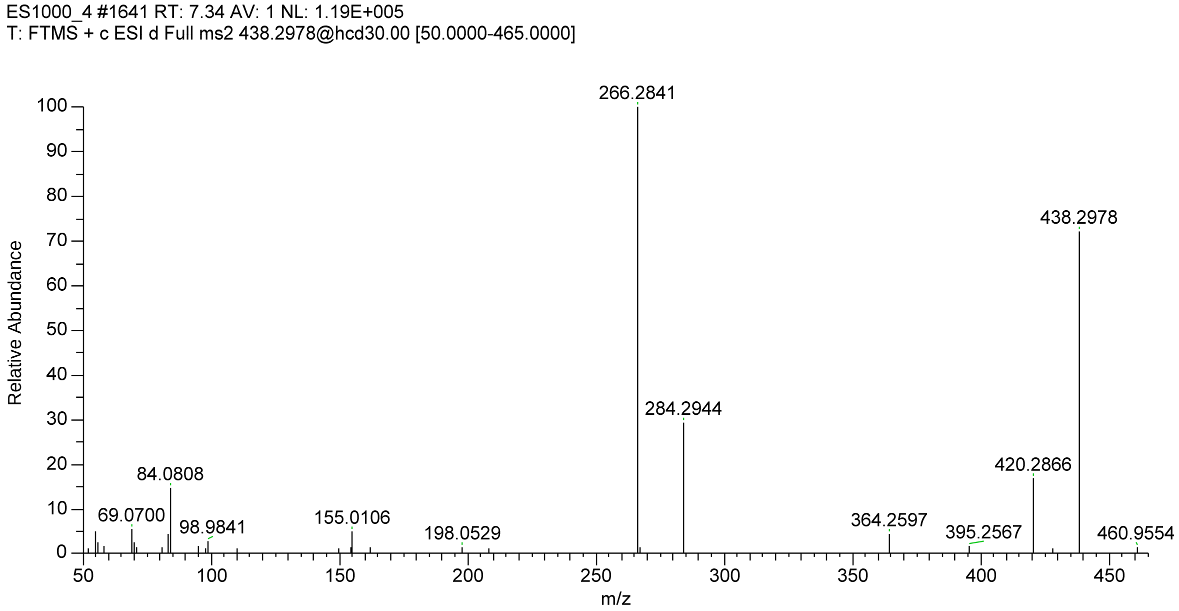

Figure S24. Collision-induced dissociation of 1-*O*-hexadec-1′-enyl-*sn*-glycero-3-phosphoethanolamine (**10**) pseudomolecular ion at *m/z* 438.2979 in coral extract

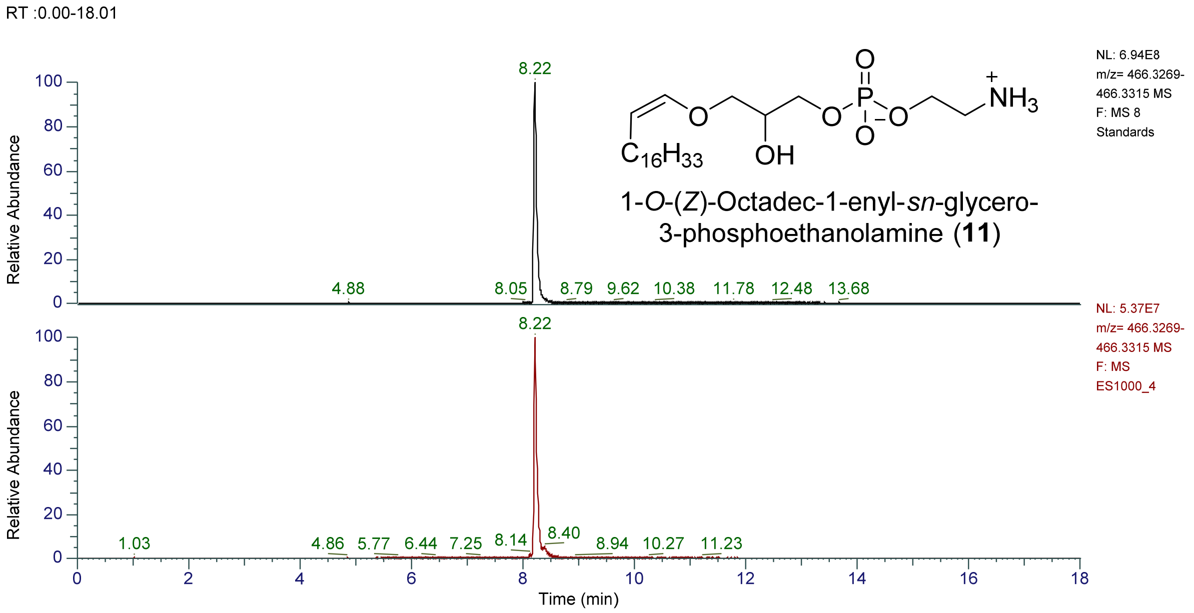

Figure S25. Extracted ion chromatograms for 1-*O*-(*Z*)-octadec-1′-enyl-*sn*-glycero-3-phosphoethanolamine (**11**) pseudomolecular ion at *m/z* 466.3292 in standard (top) and coral extract (bottom)

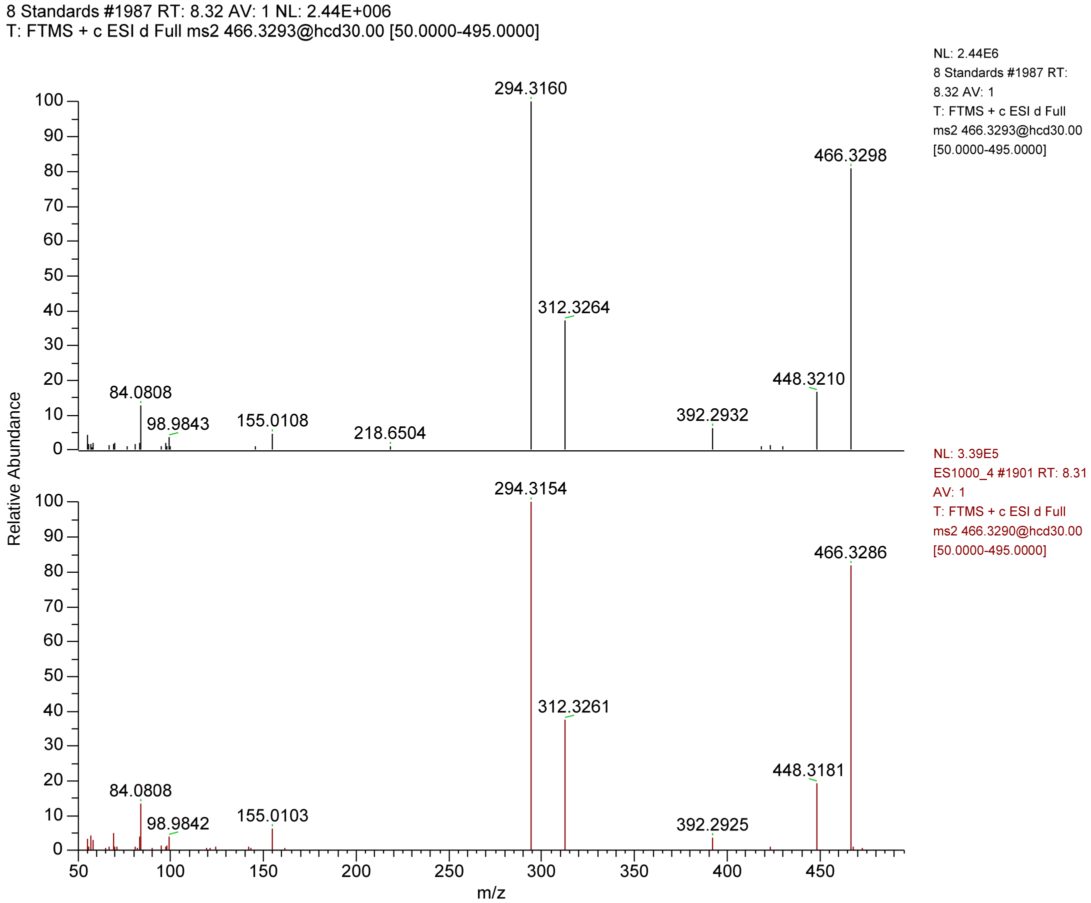

Figure S26. Compared collision-induced dissociation of 1-*O*-(*Z*)-octadec-1′-enyl-*sn*-glycero-3-phosphoethanolamine (**11**) pseudomolecular ion at *m/z* 466.3292 in standard (top) and coral extract (bottom)

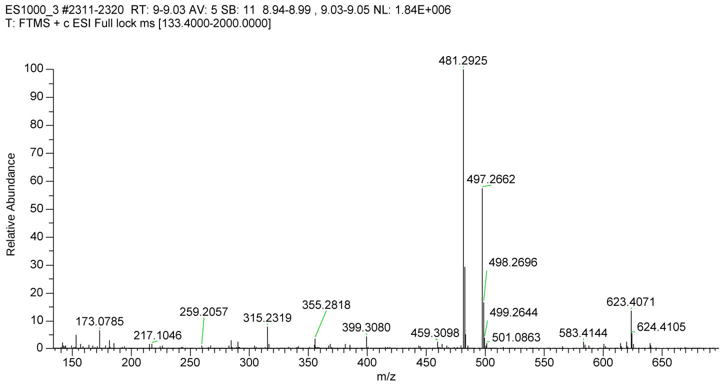

Figure S27. MS spectrum of compound **12**

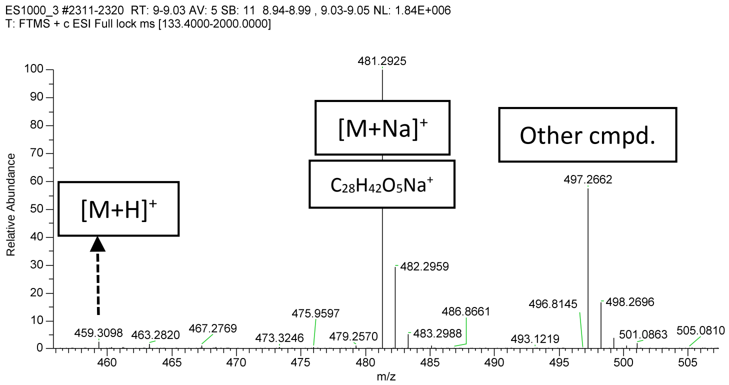

Figure S28. Expanded MS spectrum of compound **12**

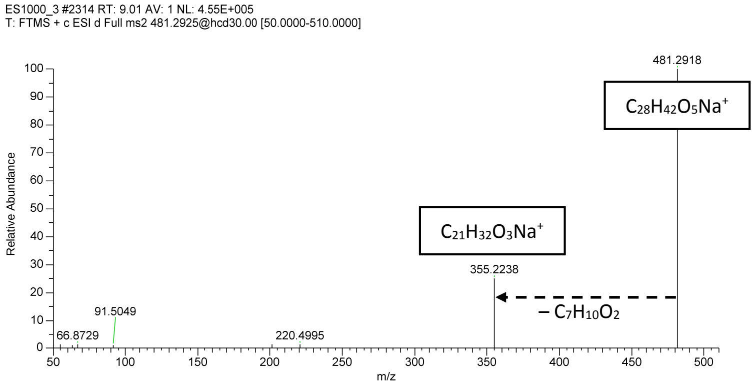

Figure S29. Collision-induced dissociation of parent ion at *m/z* 481.2925 [(**12**+Na)$^+$]

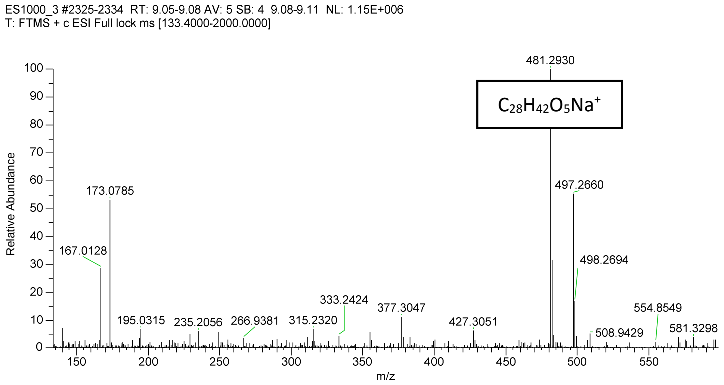

Figure S30. MS spectrum of compound **13**

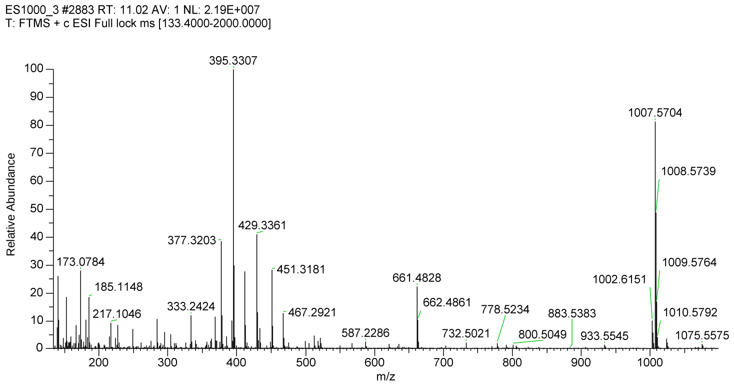

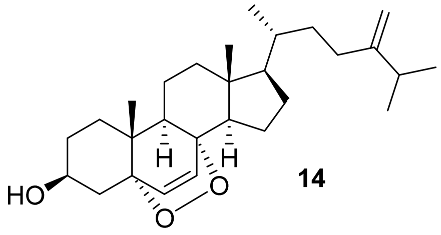

14

Figure S31. MS spectrum of compound **14**

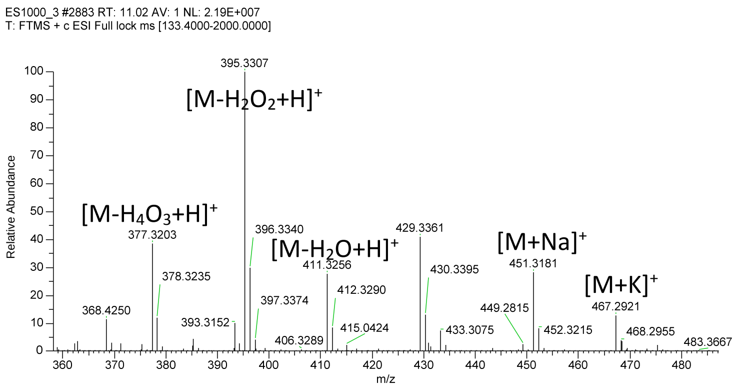

Figure S32. Expanded MS spectrum of compound **14**

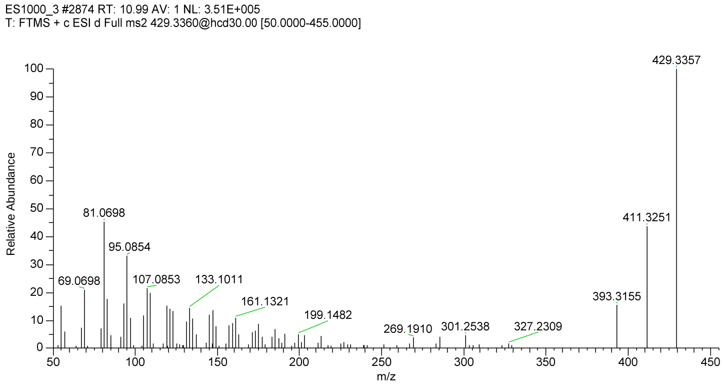

Figure S33. Collision-induced dissociation of compound **14** pseudomolecular ion at *m/z* 429.3360

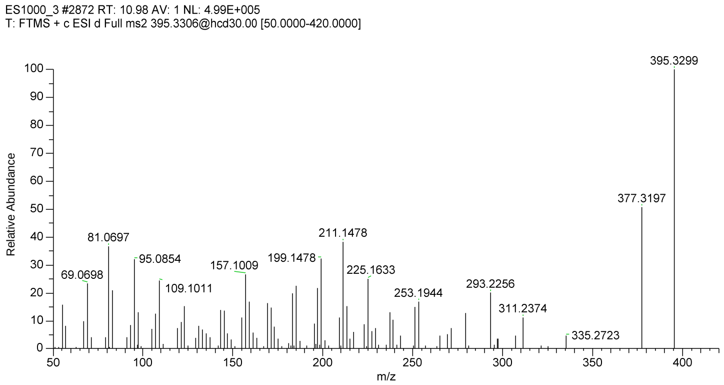

Figure S34. Collision-induced dissociation of compound **14** fragment ion at *m/z* 395.3306 ($[M-H_2O_2+H]^+$)

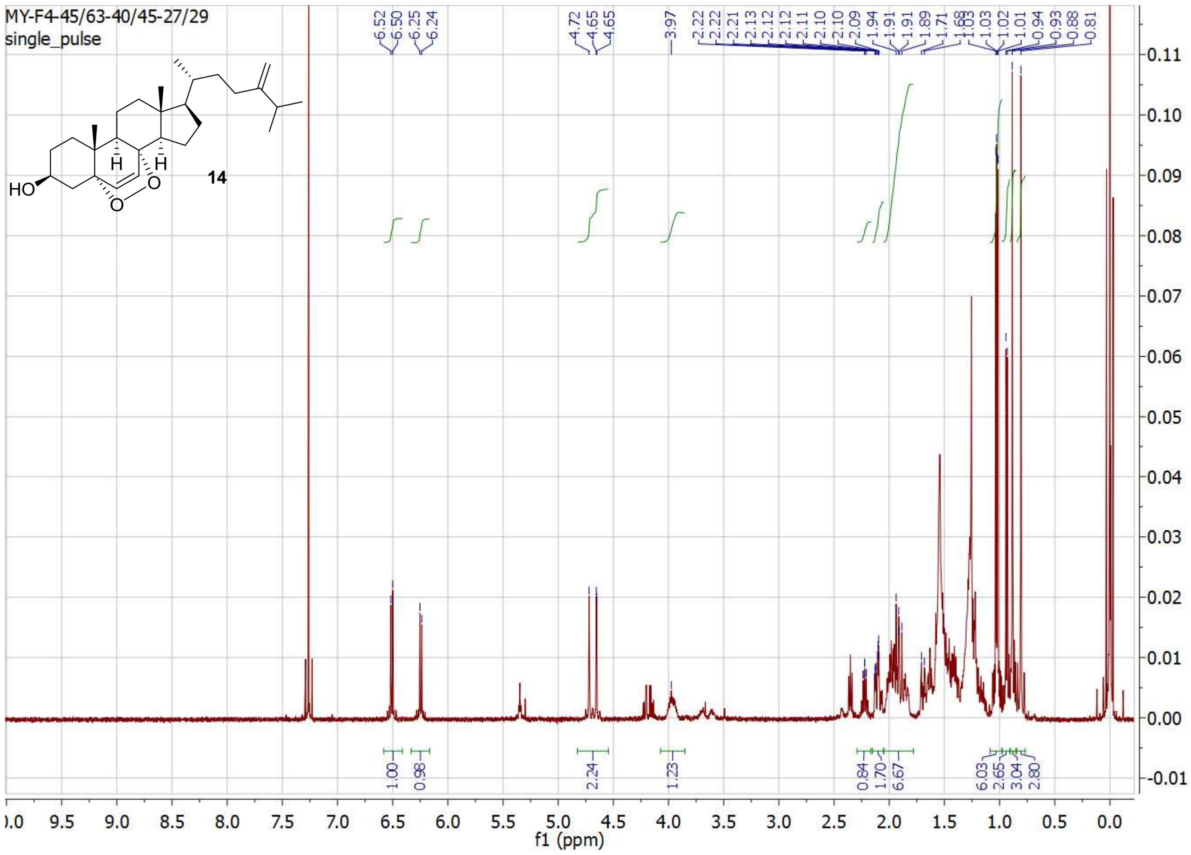

Figure S35. ^{1}H-NMR spectrum of **14** in $CDCl_3$ (500 MHz)

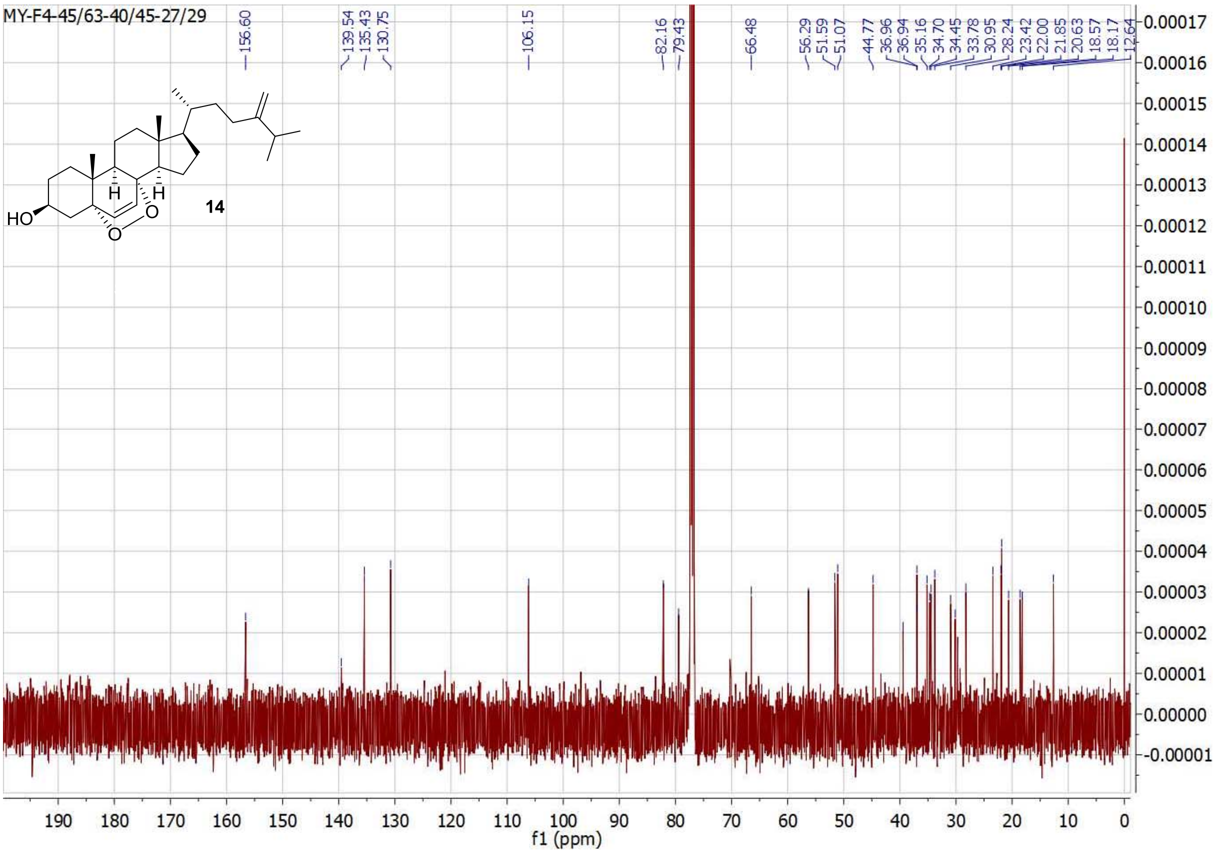

Figure S36. ^{13}C-NMR spectrum of **14** in $CDCl_3$ (125 MHz)

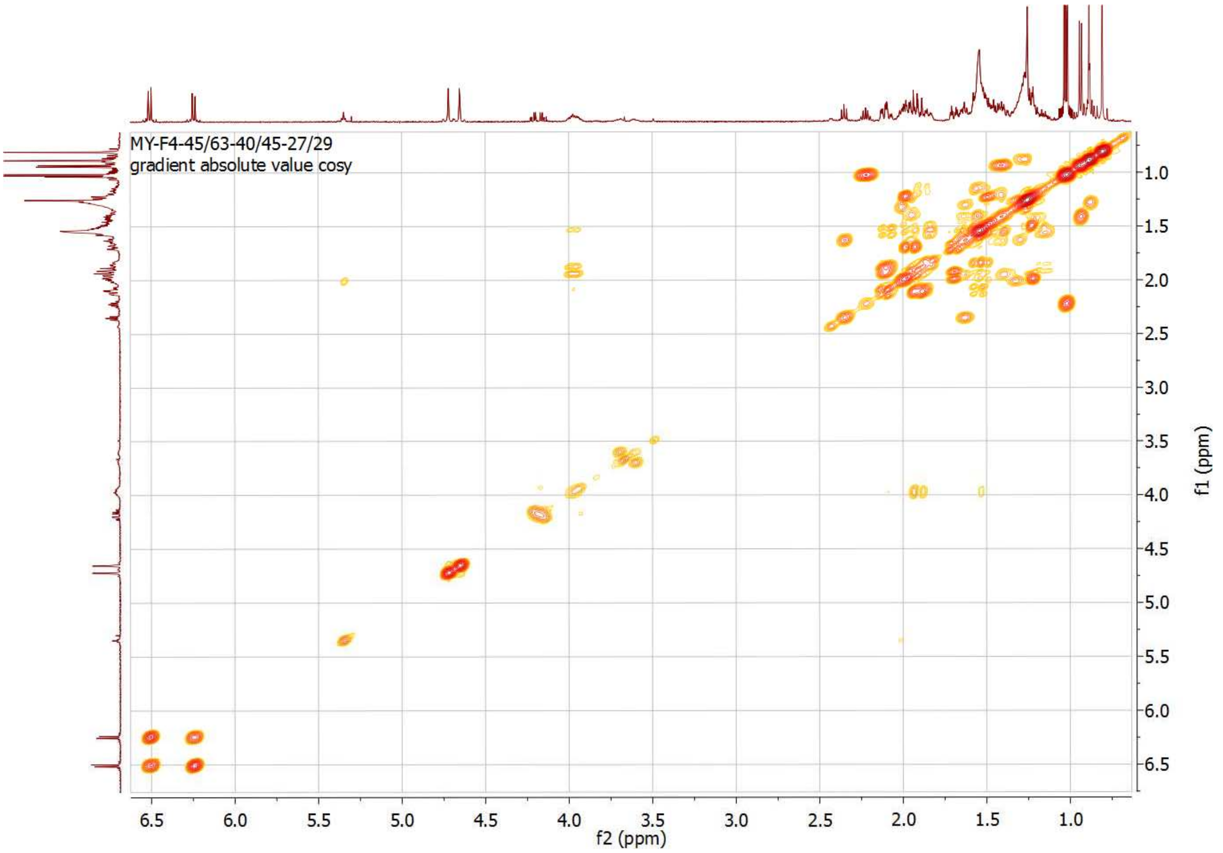

Figure S37. COSY spectrum of **14** in $CDCl_3$

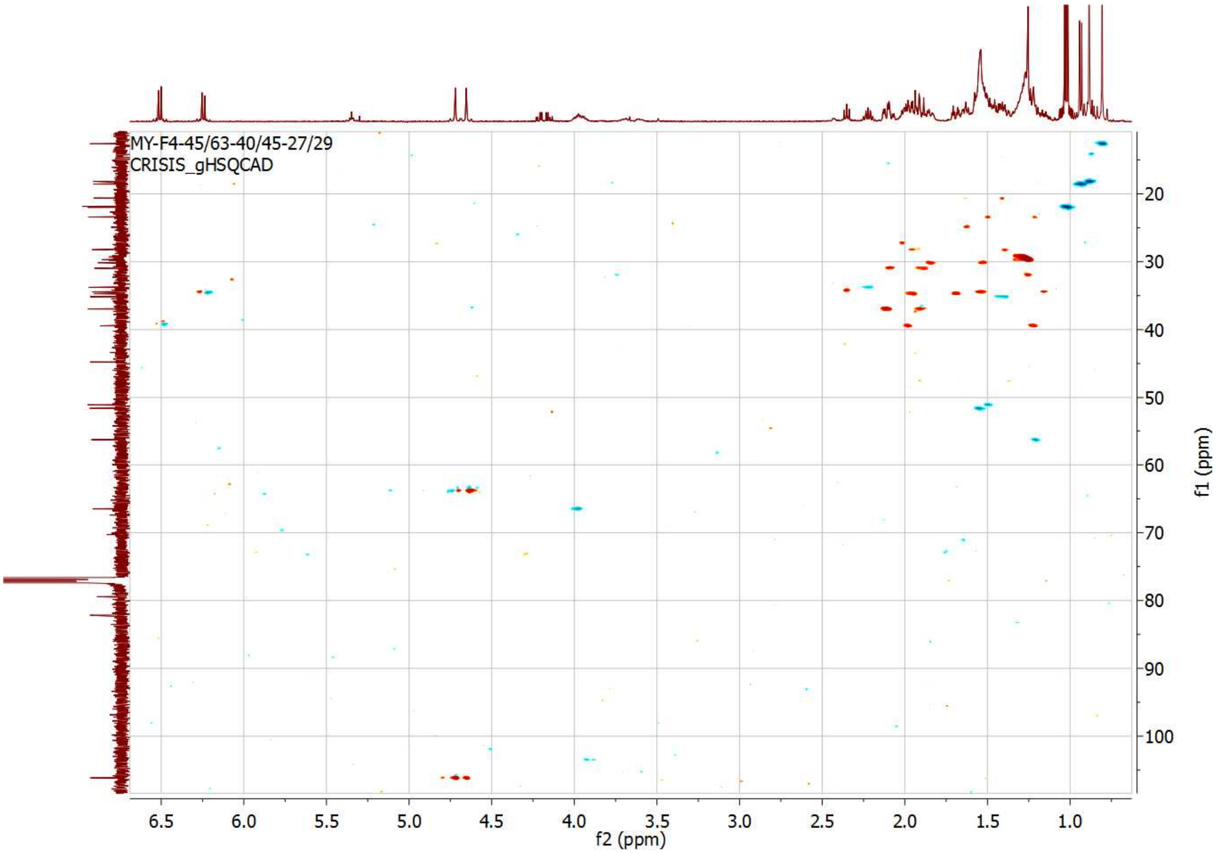

Figure S38. HSQC spectrum of **14** in $CDCl_3$

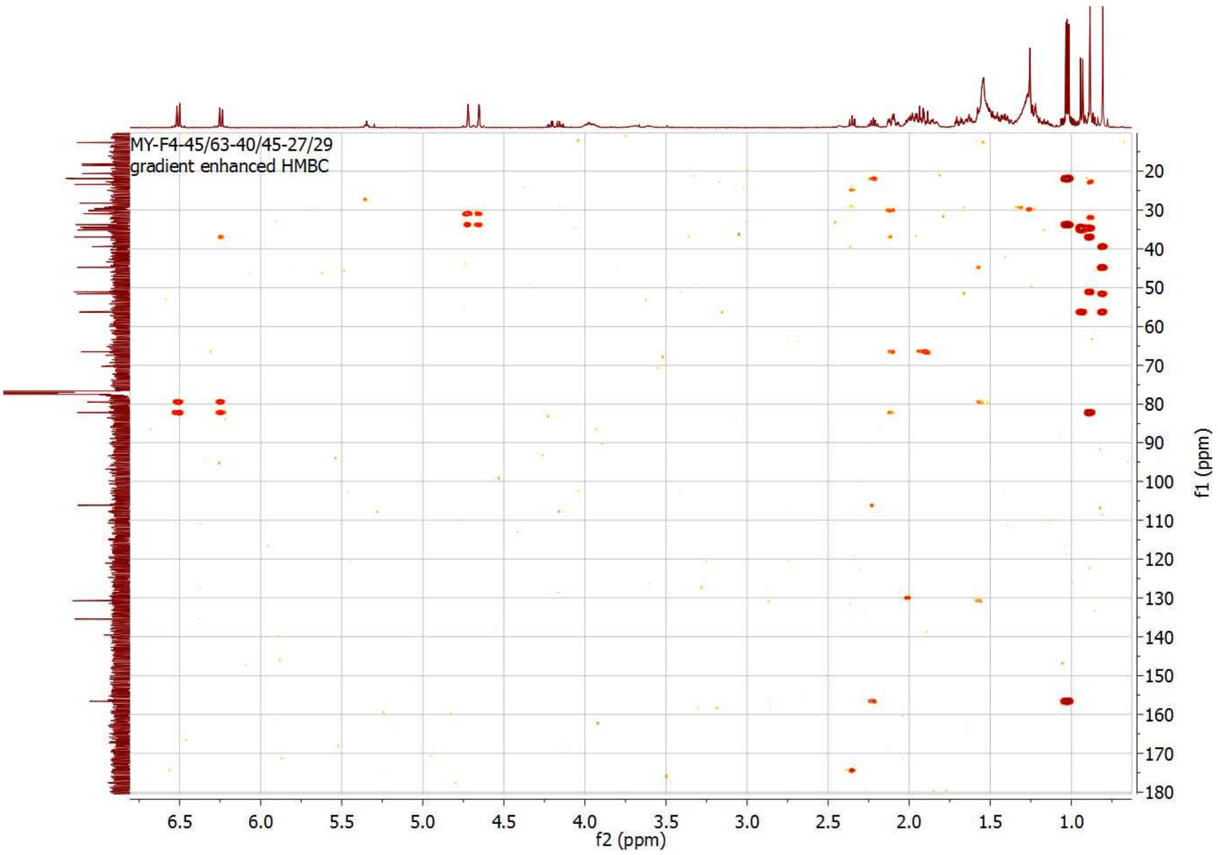

Figure S39. HMBC spectrum of **14** in $CDCl_3$

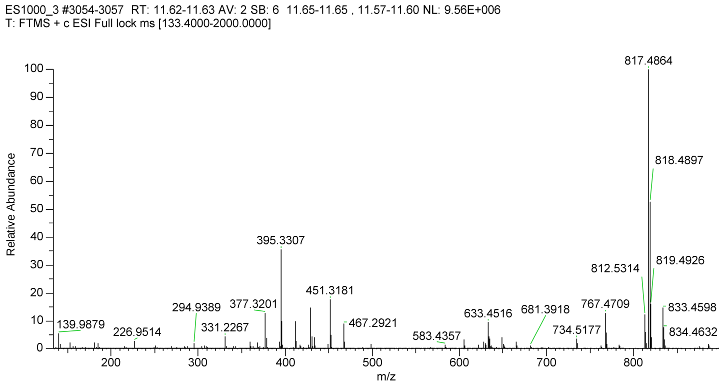

Figure S40. MS spectrum of compound **15**

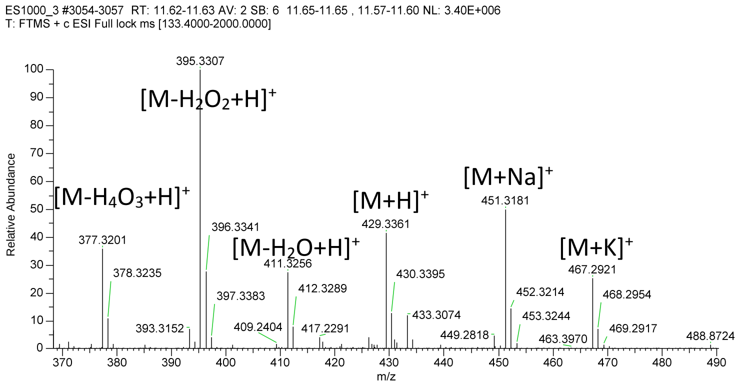

Figure S41. Expanded MS spectrum of compound **15**

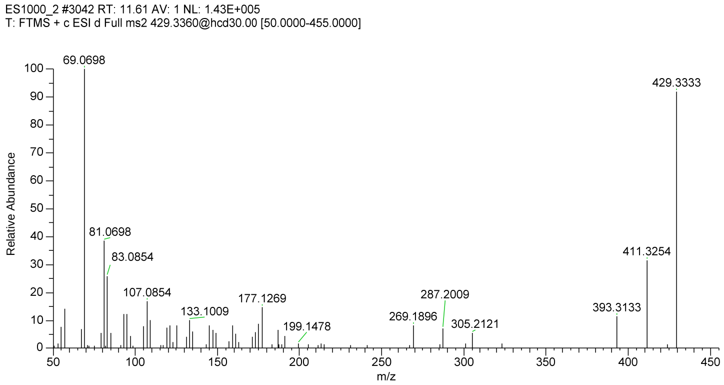

Figure S42. Collision-induced dissociation of compound **15** pseudomolecular ion at *m/z* 429.3360

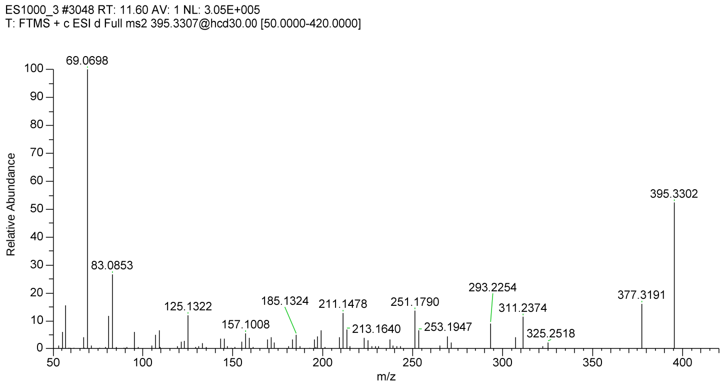

Figure S43. Collision-induced dissociation of compound **15** fragment ion at *m/z* 395.3307 ($[M-H_2O_2+H]^+$)

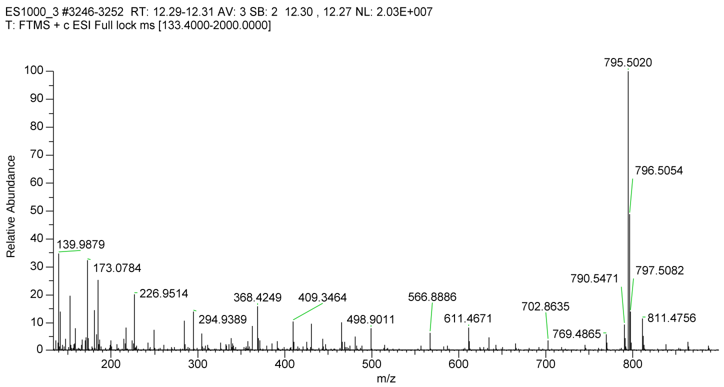

Figure S44. MS spectrum of compound **16**

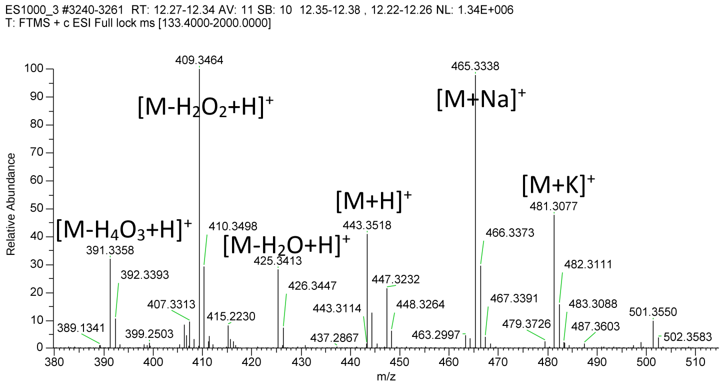

Figure S45. Expanded MS spectrum of compound **16**

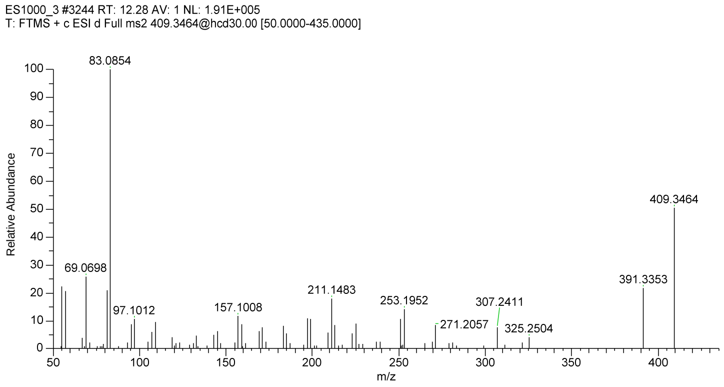

Figure S46. Collision-induced dissociation of compound **16** fragment ion at *m/z* 409.3464 ($[M-H_2O_2+H]^+$)

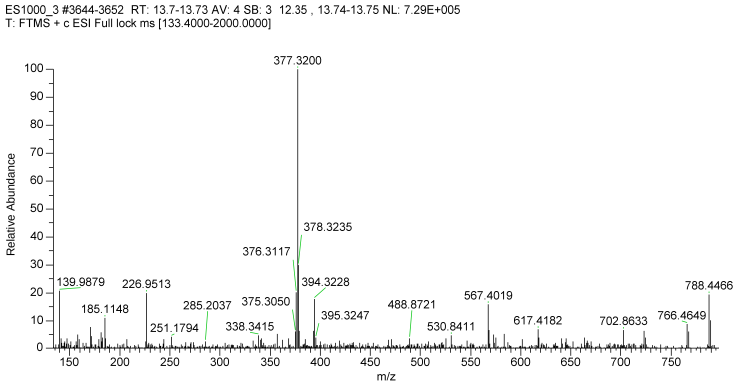

Figure S47. MS spectrum of compound **17**

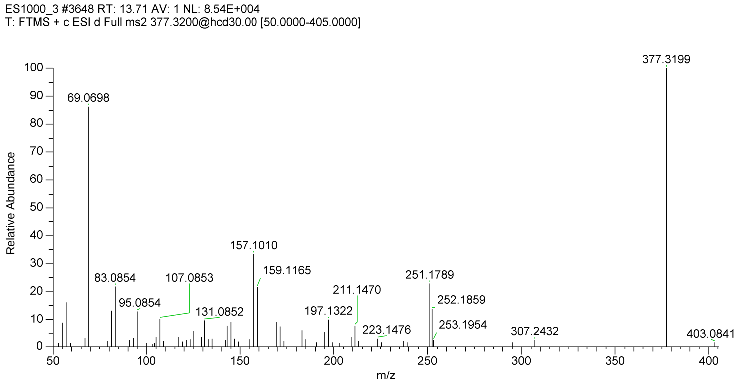

Figure S48. Collision-induced dissociation of compound **17** fragment ion at *m/z* 377.3200 (undefined fragment)

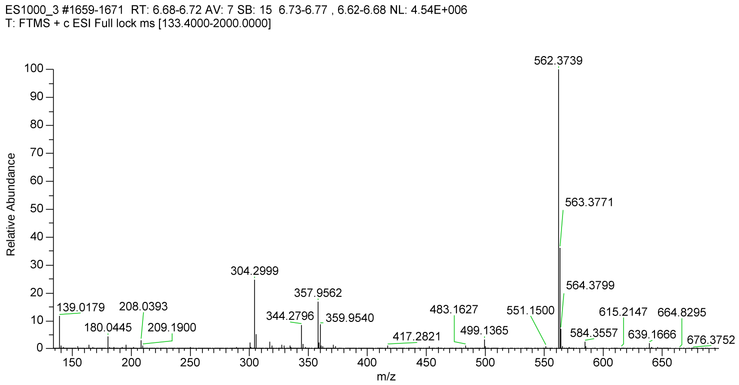

Figure S49. MS spectrum of compound **18**

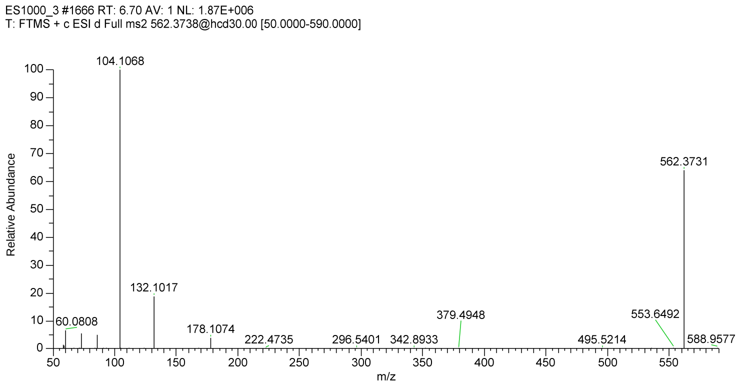

Figure S50. Collision-induced dissociation of compound **18** fragment ion at *m/z* 562.3738 ($[M+H]^+$)

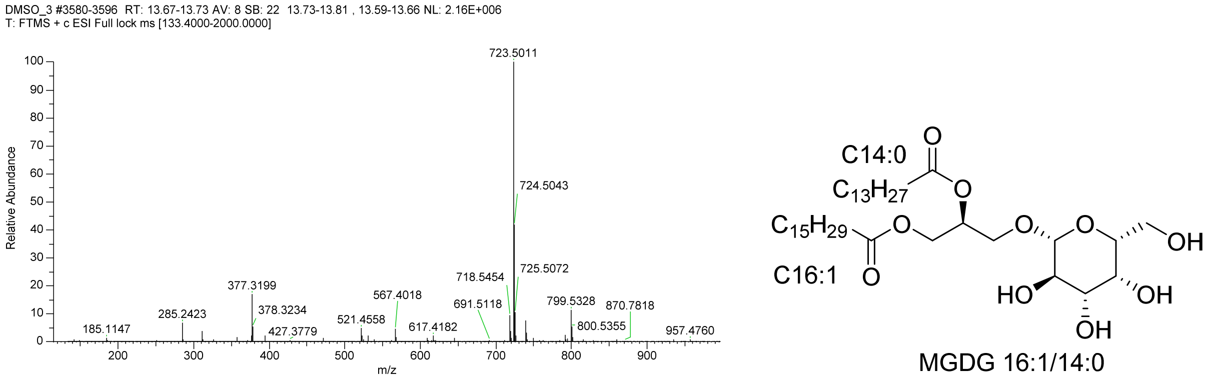

Figure S51. MS spectrum of compound **19** (t_R 13.69 min, exp. *m/z* 723.5011 corresponds to the molecular formula $C_{39}H_{72}O_{10}Na^+$, th. *m/z* 723.5018, 4 unsaturations). On the right, structure of compound **19**

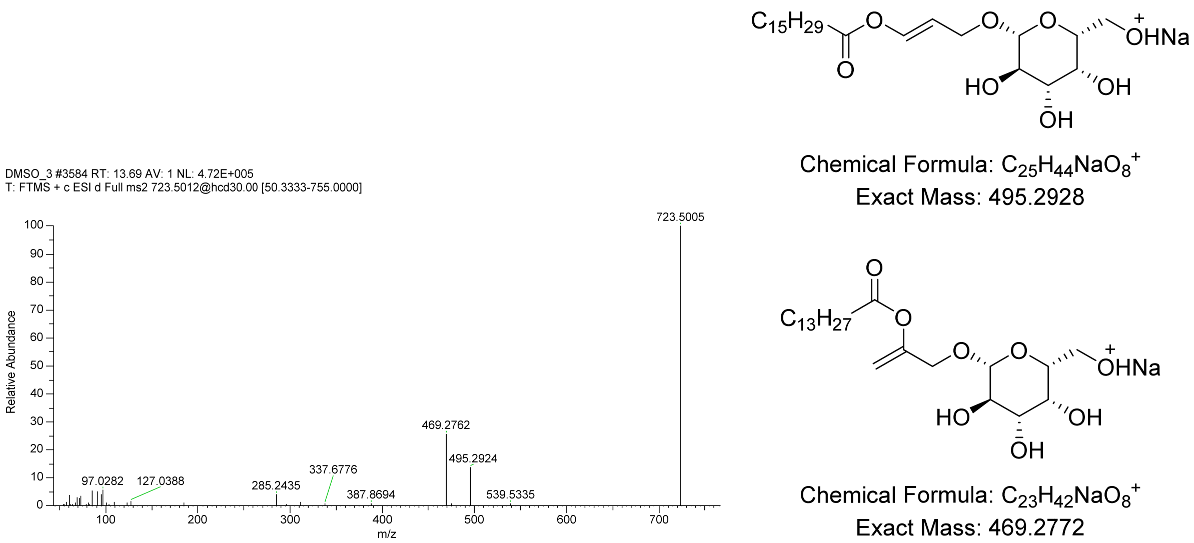

Figure S52. Collision-induced dissociation of compound **19** sodiated molecular ion at *m/z* 723.5011 ($[M+Na]^+$). On the right, key fragmentation products allowing identification of compound **19**

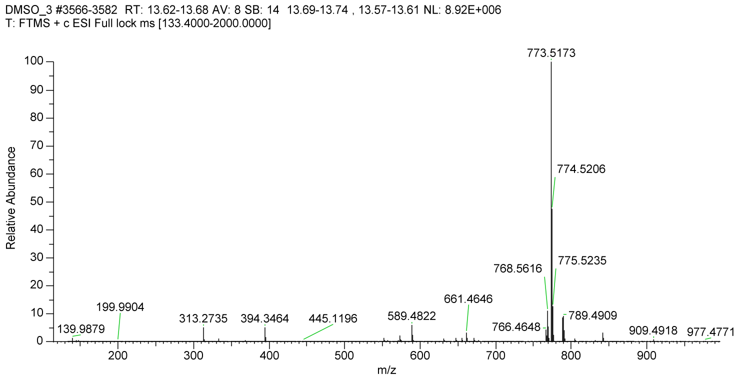

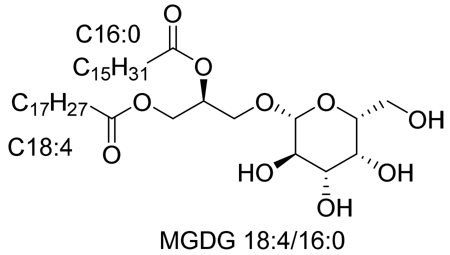

Figure S53. MS spectrum of compound **20** (t_R 13.64 min, exp. *m/z* 773.5173 corresponds to the molecular formula $C_{43}H_{74}O_{10}Na^+$, th. *m/z* 773.5174, 7 unsaturations). On the right, structure of compound **20**

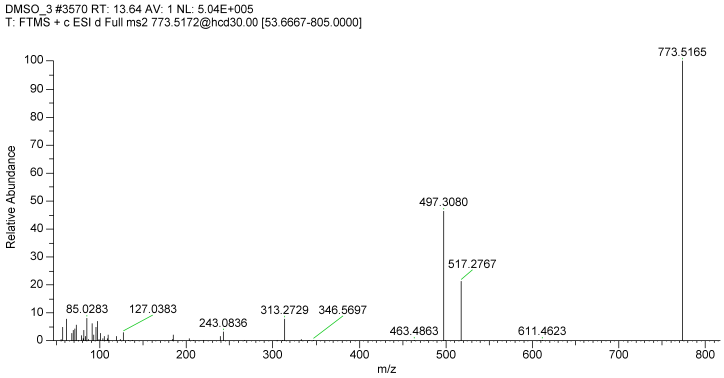

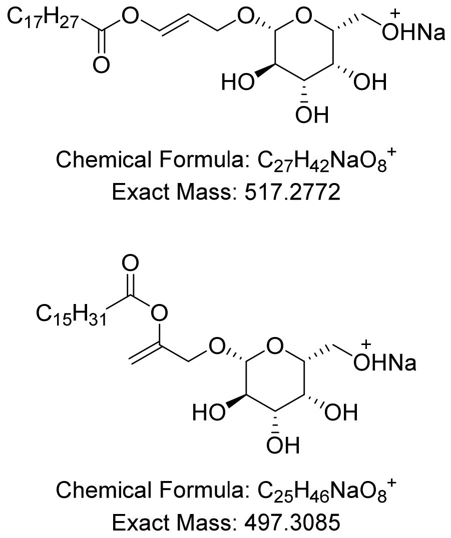

Figure S54. Collision-induced dissociation of compound **20** sodiated molecular ion at *m/z* 773.5173 ([M+Na]$^+$). On the right, key fragmentation products allowing identification of compound **20**

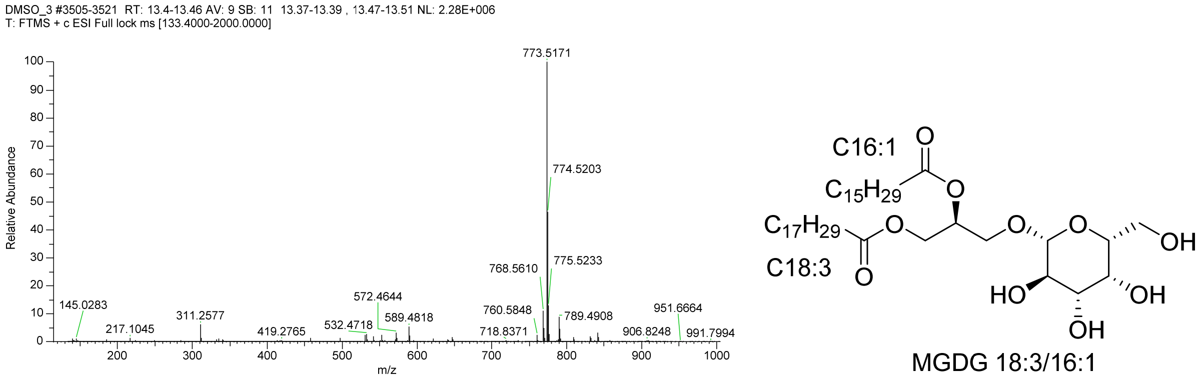

Figure S55. MS spectrum of compound **21** (t_R 13.42 min, exp. *m/z* 773.5171 corresponds to the molecular formula $C_{43}H_{74}O_{10}Na^+$, th. *m/z* 773.5174, 7 unsaturations). On the right, structure of compound **21**

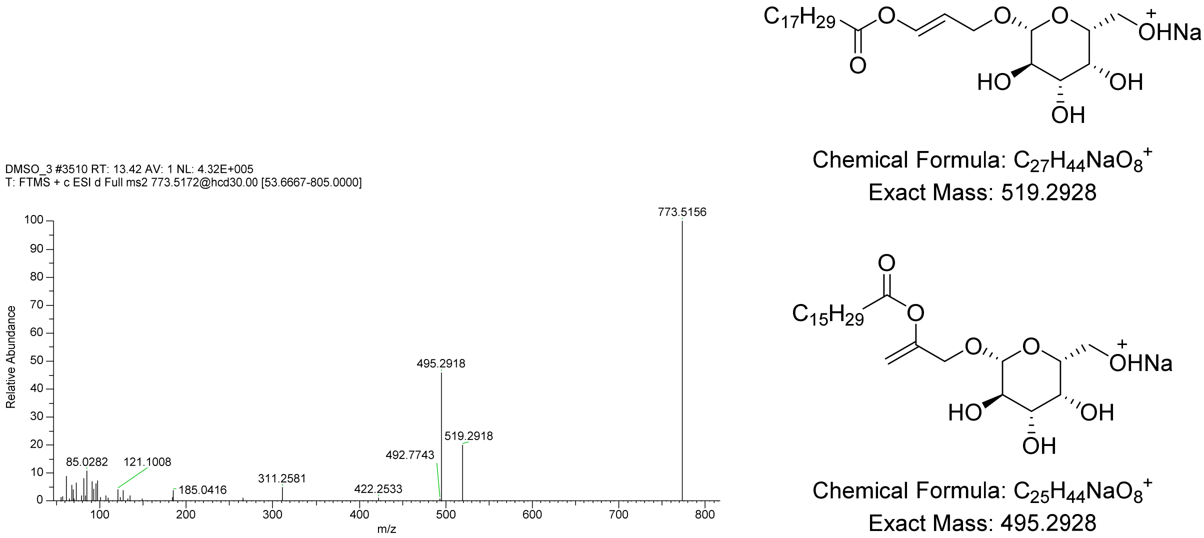

Figure S56. Collision-induced dissociation of compound **21** sodiated molecular ion at *m/z* 773.5173 ([M+Na]$^+$). On the right, key fragmentation products allowing identification of compound **21**

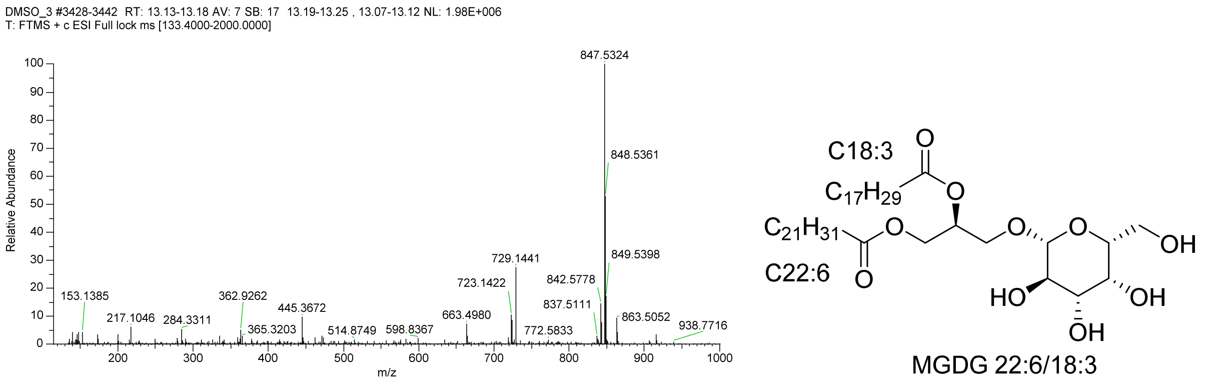

Figure S57. MS spectrum of compound **22** (t_R 13.16 min, exp. *m/z* 847.5324 corresponds to the molecular formula $C_{49}H_{76}O_{10}Na^+$, th. *m/z* 773.5331, 12 unsaturations). On the right, structure of compound **22**

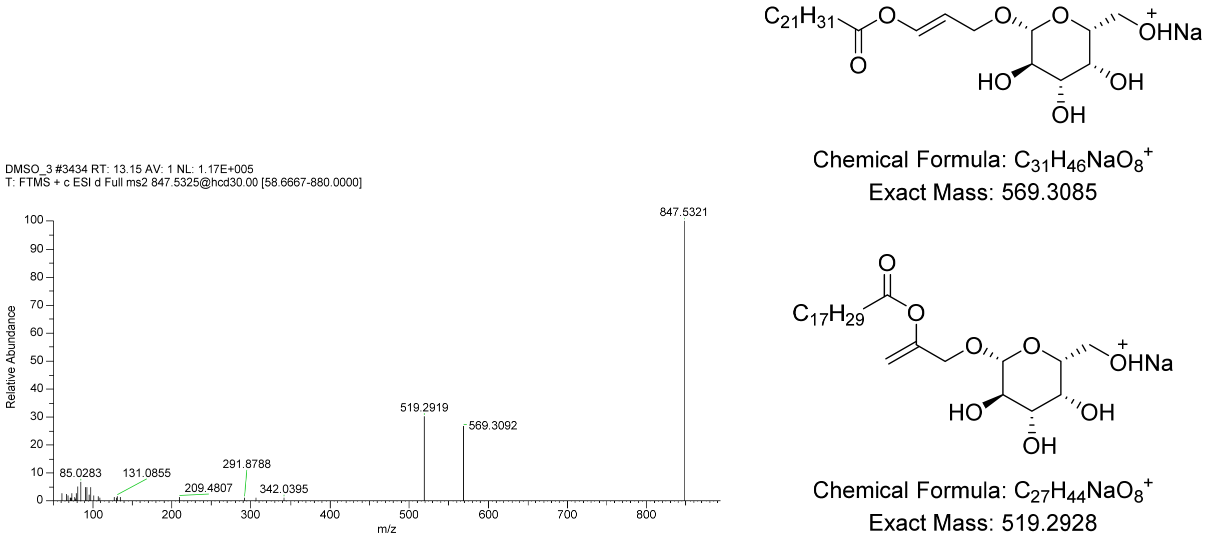

Figure S58. Collision-induced dissociation of compound **22** sodiated molecular ion at *m/z* 847.5325 ([M+Na]$^+$). On the right, key fragmentation products allowing identification of compound **22**

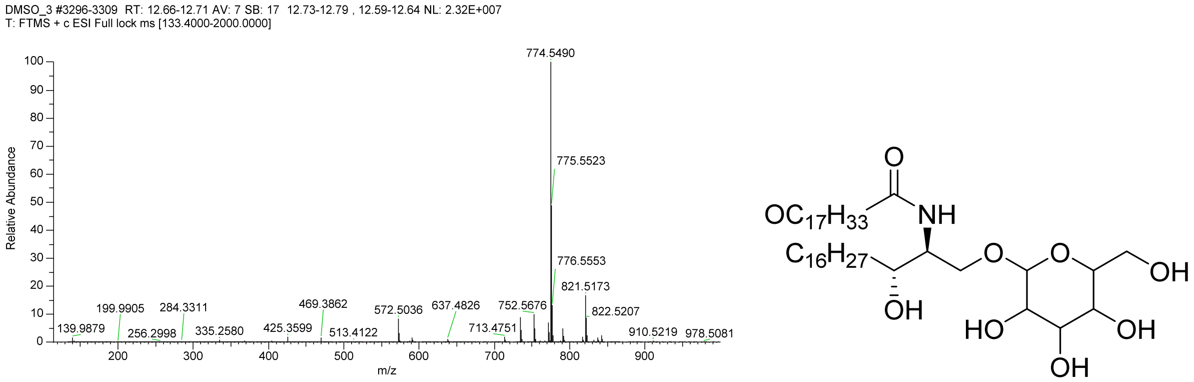

Figure S59. MS spectrum of compound **23** (t_R 12.69 min, exp. *m/z* 774.5491 corresponds to the molecular formula $C_{43}H_{77}NO_9Na^+$, th. *m/z* 774.5490, 6 unsaturations). On the right, structure of compound **23**

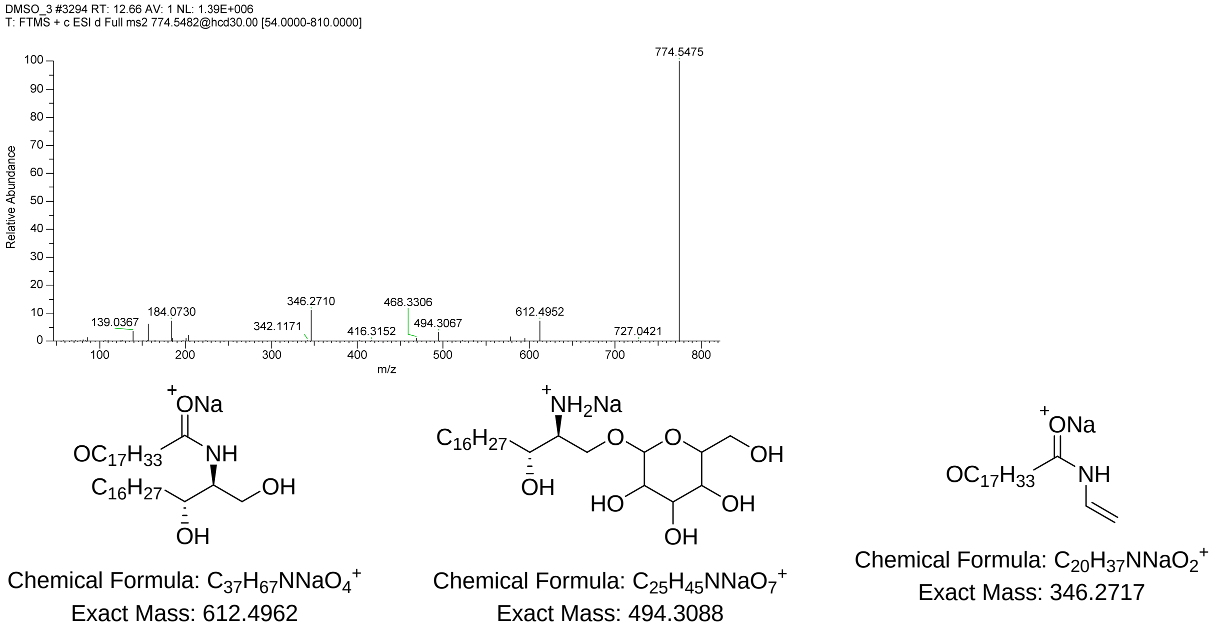

Figure S60. Collision-induced dissociation of compound **23** sodiated molecular ion at *m/z* 774.5490 ($[M+Na]^+$). Below, key fragmentation products allowing identification of compound **23**

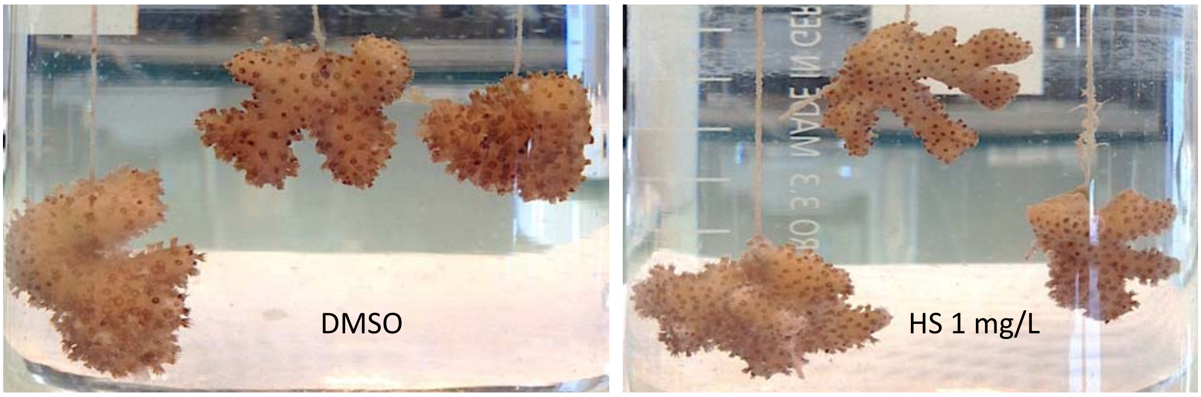

Figure S61. Picture of coral nubbins exposed at 1 mg/L homosalate (right) compared to control (left)

Table S1. Results of reciprocal *blastp* searches were also conducted using the web-based tool against the uniprot/Swiss-Prot, and model organism (landmark) databases, and is some specific cases, the conserved domains (CDD) search. In blue are proteins coded by the *P. damicornis* genome. In grey are results highlighted in the main body of the manuscript

query	database	hit	e-value	comments
**Lysophospholipid acyl transferase**				
BAF47696	Pocillopora damicornis Annotation Release 100 proteins	XP_027038685	3.00E-160	query: human LPCAT2,
BAF47696	Pocillopora damicornis Annotation Release 100 proteins	XP_027038692	8.00E-99	query: human LPCAT2,
XP_027038685	model organisms landmark	NP_766602	2.00E-156	Hit : mouse LPCAT2, HXXXXD motif essential for acyltransferase activity present
XP_027038685	uniProtKB/Swiss-Prot	Q8BYI6	2.00E-156	Hit : mouse LPCAT2
XP_027038685	conserved domains CDD v 36 50369 PSSMs	cd07991+C9	4.65E-96	Lysophospholipid Acyltransferases (LPLATs) of Glycerophospholipid Biosynthesis: LPCAT1-like; contains feature "putative acyl-acceptor binding pocket"
XP_027038685	conserved domains CDD v 36 50369 PSSMs	cd16185	3.29E-14	EFh_PEF_ALG-2_like, EF-hand, calcium binding motif, found in homologs of mammalian apoptosis-linked gene 2 protein (ALG-2) : contains calcium binding sites, good match to 5 of 7 features of the conserved domain, mouse hits a different cdd.
XP_027038692	model organisms landmark	NP_766602	5.00E-101	Hit : mouse LPCAT2 also close to mouse LPCAT1
XP_027038692	uniProtKB/Swiss-Prot		4.00E-101	Hit : mouse LPCAT2
XP_027038692	conserved domains CDD v 36 50369 PSSMs	cd07991	1.93E-71	Lysophospholipid Acyltransferases (LPLATs) of Glycerophospholipid Biosynthesis: LPCAT1-like; contains feature putative acyl-acceptor binding pocket

XP_027038692	conserved domains CDD v 36 50369 PSSMs	cd15898	1.25E-03	EF-hand motif found in eukaryotic phosphoinositide-specific phospholipase C (PI-PLC, EC 3.41) isozymes : contains calcium binding sites, good match to 2 of 4 features of the conserved domain, mouse hits a different cdd.
**PAF acyl hydrolase**				
NP_005075	Pocillopora damicornis Annotation Release 100 proteins	XP_027041233	2.00E-73	query : human PAF-AH lp-LPA2
XP_027041233	model organisms landmark	NP_998354	1.00E-81	Plasma form of PAF-AH, less specific for acetyl in sn-2 contains GXSXG motif
XP_027041233	uniProtKB/Swiss-Prot	Q90678	5.00E-80	Plasma form of PAF-AH
XP_027041233	conserved domains CDD v 36 50369 PSSMs	pfam03403	5.06E-114	Platelet-activating factor acetylhydrolase, isoform II;
**secreted Phospholypase A2**				
XP_015780732	Acropora digitifera Annotation Release 100 proteins	all KEGG annotated sPLA2	2.5-1.00E-81	query, A digitifera sPLA2 annotated by kegg. Misses Cytosolic PLA2
XP_015775072	Acropora digitifera Annotation Release 100 proteins	XP_015768685	6.00E-141	cytosolic PLA2 only typeIV specific for AA

XP_015780732	Pocillopora damicornis Annotation Release 100 proteins	XP_027045719<br>XP_027045720<br>XP_027045664<br>XP_027050230<br>XP_027050243<br>XP_027047046<br>XP_027045600<br>XP_027045607<br>XP_027047608<br>XP_027048972<br>XP_027040584<br>XP_027040594<br>XP_027041954<br>XP_027036734	2.00-7.00e-26	query, A digitifera sPLA2 annotated by kegg. Misses Cytosolic PLA2
XP_015767581	Pocillopora damicornis Annotation Release 100 proteins	XP_027038035	2.00E-95	query: Acropora digitifera sPLA2 GroupXII
XP_027038035	conserved domains CDD v 36 50369 PSSMs	cl23566 pfam06951	2.20E-30	sPLA2 GroupXII
XP_027038035	model organisms landmark	NP_648815	4.00E-23	sPLA2 GroupXII, Hypothesized to be involved in the production of ecosanoids, but experimentally added enzyme does not induce production of AA from rat cells.
XP_027038035	uniProtKB/Swiss-Prot	Q9BZM1	1.00E-19	sPLA2 GroupXII Human
XP_027045719	model organisms landmark	NP_000919	7.00E-23	sPLA2 Group IB stimulate CXCL8 but not through enzymatic activity (no AA production). Suggested to be involved in lipid digestion
XP_027045719	uniProtKB/Swiss-Prot	P08873	3.00E-32	Snake venom phospholipase A2 (PLA2) that has no lethal activity. PLA2 catalyzes the calcium-dependent hydrolysis of the 2-acyl groups in 3-sn-phosphoglycerides

XP_027045720	model organisms landmark	NP_035237	5.00E-27	sPLA2 Group IB Mus musculus
XP_027045720	uniProtKB/Swiss-Prot	Q9Z0Y2	4.00E-27	mouse sPLA2 IB see comment above
XP_027045664	model organisms landmark	NP_001107095	5.00E-22	sPLA2 Group IB Danio rerio
XP_027045664	uniProtKB/Swiss-Prot	P00614	1.00E-28	Snake venom phospholipase A2 (PLA2) heterotrimer that acts as a potent presynaptic neurotoxin by blocking synaptic transmission and synaptic vesicle recycling
XP_027050230	model organisms landmark	NP_001107095	9.00E-18	sPLA2 Group IB Danio rerio
XP_027050230	uniProtKB/Swiss-Prot	D2X8K2	1.00E-28	sea anemone Condylactis gigantea phospholipase A2 (PLA2) - Nematocyst
XP_027050243	model organisms landmark	NP_055404	1.00E-16	sPLA2 Group IIE. Human Unknown Function, can produce AA in mice
XP_027050243	uniProtKB/Swiss-Prot	D2X8K2	6.00E-27	sea anemone Condylactis gigantea phospholipase A2 (PLA2) - Nematocyst
XP_027047046	model organisms landmark	NP_036174	1.00E-16	sPLA2 Group IIE Mus musculus
XP_027047046	uniProtKB/Swiss-Prot	P59171	9.00E-21	Snake Echis ocellatus Expressed by the venom gland. Belongs to the phospholipase A2 family. Group II subfamily. D49 sub-subfamily
XP_027045600	model organisms landmark	NP_035237	1.00E-22	sPLA2 Group IB Mus musculus
XP_027045600	uniProtKB/Swiss-Prot	D2X8K2	7.00E-28	sea anemone Condylactis gigantea phospholipase A2 (PLA2) - Nematocyst
XP_027045607	model organisms landmark	NP_001107095	4.00E-28	sPLA2 Group IB Danio rerio
XP_027045607	uniProtKB/Swiss-Prot	Q8JFG2	2.00E-32	Snake Laticauda semifasciata pancreas. Belongs to the phospholipase A2 family. Group I
XP_027047608	model organisms landmark	NP_001107095	7.00E-28	sPLA2 Group IB Danio rerio
XP_027047608	uniProtKB/Swiss-Prot	P00606	3.00E-30	Snake Bungarus multicinctus Expressed by the venom gland. Belongs to the phospholipase A2 family. Group I subfamily. D49 sub-subfamily
XP_027048972	model organisms landmark	NP_001107095	2.00E-22	sPLA2 Group IB Danio rerio

XP_027048972	UniProtKB/Swiss-Prot	Q8WS88	3.00E-28	sea anemone Adamsia paliata phospholipase A2 (PLA2) - Nematocyst
XP_027040584	model organisms landmark	NP_000291	7.00E-21	sPLA2 Group IIA Human. thought to be one of the key enzymes in the pathogenesis of inflammatory diseases
XP_027040584	uniProtKB/Swiss-Prot	D2X8K2	1.00E-29	sea anemone Condylactis gigantea phospholipase A2 (PLA2) - Nematocyst
XP_027040594	model organisms landmark	NP_035237	2.00E-10	sPLA2 Group IB Mus musculus
XP_027040594	uniProtKB/Swiss-Prot	D2X8K2	6.00E-17	sea anemone Condylactis gigantea phospholipase A2 (PLA2) - Nematocyst
XP_027041954	model organisms landmark	NP_998523	6.00E-170	Not sPLA2
XP_027036734	model organisms landmark	XP_642307	2.00E-31	Not sPLA2
**cytosolic Phospholypase A2**				
XP_015775072	Pocillopora damicornis Annotation Release 100 proteins	XP_027046702<br>XP_027037214<br>XP_027057234<br>XP_027049187<br>XP_027037321	5e-177-0.00	query : cPLA2 A digitifera
XP_027046702	model organisms landmark	NP_077734	0	Human cytosolic phospholipase A2 group IV family. The enzyme catalyzes the hydrolysis of membrane phospholipids to release arachidonic acid which is subsequently metabolized into eicosanoids
XP_027046702	uniProtKB/Swiss-Prot	B1WAZ6	0	Xenopus tropicalis cPLA2 group IVA
XP_027037214	model organisms landmark	NP_077734	0	Human cPLA2 group IV family

XP_027037214	uniProtKB/Swiss-Prot	B1WAZ6	0	Xenopus tropicalis cPLA2 group IVA
XP_027057234	model organisms landmark	NP_001292561	0	Mus musculus cPLA2 group IV family
XP_027057234	uniProtKB/Swiss-Prot	B1WAZ6	0	Xenopus tropicalis cPLA2 group IVA
XP_027049187	model organisms landmark	NP_571370	6.00E-73	Danio rerio cPLA2 group IVA
XP_027049187	uniProtKB/Swiss-Prot	B1WAZ6	0	Xenopus tropicalis cPLA2 group IVA
XP_027037321	model organisms landmark	XP_017208191	5.00E-102	Danio rerio cPLA2 group IVA
XP_027037321	uniProtKB/Swiss-Prot	B1WAZ6	2.00E-104	Xenopus tropicalis cPLA2 group IVA
**Cyclooxygenases**				
NP_035328.2	Pocillopora damicornis Annotation Release 100 proteins	XP_027039774	5.00E-19	query: *Mus musculus* COX2
XP_027039774	model organisms landmark	NP_177509	3.00E-109	*A thaliana* Alpha Dioxygenase
XP_006497857	Pocillopora damicornis Annotation Release 100 proteins	XP_027043448	9.00E-23	*query: Mus musculus COX1*
XP_027043448	model organisms landmark	XP_642775	4.00E-96	animal heme peroxidase family protein
AAF93169	Pocillopora damicornis Annotation Release 100 proteins	XP_027039809	2.00E-20	*query: Plexaura homomalla* COX
XP_027039809	model organisms landmark	NP_177509	2.00E-104	*A thaliana* Alpha Dioxygenase
**Lypooxygenases**				
AHK60922:1-373	Pocillopora damicornis Annotation Release 100 proteins	XP_027058643<br>XP_027058642<br>XP_027058639<br>XP_027058638<br>XP_027058637<br>XP_027058636<br>XP_027047440<br>XP_027047439<br>XP_027047454	4e-69-2e-92	Query : C. imbricata AOS

AHK60922:374-1066	Pocillopora damicornis Annotation Release 100 proteins	XP_027058636<br>XP_027058639<br>XP_027058638<br>XP_027058637<br>XP_027047440<br>XP_027047439<br>XP_027058643<br>XP_027058642<br>XP_027058640<br>XP_027047466<br>XP_027048285<br>XP_027047963<br>XP_027038362<br>XP_027056548<br>XP_027056547<br>XP_027056546<br>XP_027036058<br>XP_027036056<br>XP_027036057<br>XP_027051170<br>XP_027036041<br>XP_027036755<br>XP_027055283<br>XP_027058330<br>XP_027058329<br>XP_027058331	1e-34-0.0 Query : C. imbricata LOX

XP_015762909	Pocillopora damicornis Annotation Release 100 proteins	XP_027058639<br>XP_027058638<br>XP_027058637<br>XP_027058643<br>XP_027058642<br>XP_027058636<br>XP_027058640<br>XP_027047466<br>XP_027047440<br>XP_027047439<br>XP_027047963<br>XP_027048285<br>XP_027036058<br>XP_027036057<br>XP_027036056<br>XP_027051170<br>XP_027036041<br>XP_027056548<br>XP_027056547<br>XP_027056546<br>XP_027038362<br>XP_027055283<br>XP_027036755	Annotated as a 15-LOX from A digitifera in KEGG

XP_027058636	uniProtKB/Swiss-Prot	O16025	0	Allene oxide synthase-lipoxygenase protein Bifunctional enzyme which is responsible for allene oxide biosynthesis via a two-step reaction which involves conversion of arachidonic acid to a 8R-hydroperoxide intermediate followed by conversion of the hydroperoxide to allene oxide.
XP_027058637 XP_027058638 XP_027058639	uniProtKB/Swiss-Prot	O16025	0	Allene oxide synthase-lipoxygenase protein
XP_027047440	uniProtKB/Swiss-Prot	O16025	0	Allene oxide synthase-lipoxygenase protein
XP_027047439	uniProtKB/Swiss-Prot	O16025	0	Allene oxide synthase-lipoxygenase protein
XP_027058642 XP_027058643	uniProtKB/Swiss-Prot	O16025	0	Allene oxide synthase-lipoxygenase protein
XP_027058640	uniProtKB/Swiss-Prot	O16025	0	Allene oxide synthase-lipoxygenase protein
XP_027047466	uniProtKB/Swiss-Prot	O16025	0	Allene oxide synthase-lipoxygenase protein only lypooxygenase domain
XP_027048285	uniProtKB/Swiss-Prot	P09917	3.00E-119	Arachidonate 5-lipoxygenase Catalyzes the first step in leukotriene biosynthesis EC: 1.13.11.34 Reaction=(5Z,8Z,11Z,14Z)-eicosatetraenoate + O2 = H2O + leukotriene A4, 5 of 7 positions linked to activity (Human) present
XP_027047963	uniProtKB/Swiss-Prot	P09917	1.00E-07	see XP_027048285
XP_027056548	uniProtKB/Swiss-Prot	P09917	5.00E-55	Arachidonate 5-lipoxygenase
XP_027038362	uniProtKB/Swiss-Prot	P09917	6.00E-47	Arachidonate 5-lipoxygenase
XP_027051170	uniProtKB/Swiss-Prot	O16025	4.00E-43	Allene oxide synthase-lipoxygenase protein only lypooxygenase domain
XP_027036058	uniProtKB/Swiss-Prot	P12527	4.00E-50	Arachidonate 5-lipoxygenase
XP_027036056	uniProtKB/Swiss-Prot	P12527	1.00E-49	Arachidonate 5-lipoxygenase
XP_027036057	uniProtKB/Swiss-Prot	P12527	1.00E-49	Arachidonate 5-lipoxygenase

XP_027036041	uniProtKB/Swiss-Prot	P09917	7.00E-44	Arachidonate 5-lipoxygenase
XP_027055283.	uniProtKB/Swiss-Prot	P51399	9.00E-54	Arachidonate 5-lipoxygenase, only 64% aligned, 93% aligned with D3ZQF9 (lipoxygenase, epidermal-type) but at 5e-25 e value, May preferentially convert arachidonic acid to (12S)-hydroperoxyeicosatetraenoic acid/(12S)-HPETE.
XP_027036755	uniProtKB/Swiss-Prot	P48999	3.00E-49	Arachidonate 5-lipoxygenase
XP_027058330 XP_027058329	uniProtKB/Swiss-Prot	O16025	1.00E-38	Allene oxide synthase-lipoxygenase protein
XP_027058331	uniProtKB/Swiss-Prot	O16025	1.00E-38	Allene oxide synthase-lipoxygenase protein

Leukotriene biosynthesis

Q16873	Pocillopora damicornis Annotation Release 100 proteins	no hits		Human Leukotriene C4 synthase; Catalyzes the conjugation of leukotriene A4 with reduced glutathione to form leukotriene C4

P09960	Pocillopora damicornis Annotation Release 100 proteins	XP_027049104	0	Human Leukotriene A-4 hydrolase Epoxide hydrolase that catalyzes the final step in the biosynthesis of the proinflammatory mediator leukotriene B4. Has also aminopeptidase activity
XP_027049104	uniProtKB/Swiss-Prot	P09960	0	Leukotriene A-4 hydrolase
XP_027049104	conserved domains CDD v 36 50369 PSSMs	cl20139:TIGR02411	0	leukotriene A-4 hydrolase/aminopeptidase
XP_027049104:505-662	pfam	PF09127	1.50E-35	Leukotriene A4 hydrolase, C-terminal
XP_027049104:247-448	pfam	PF01433	2.00E-46	Peptidase M1
XP_027049104:28-207	pfam	PF17900	9.00E-25	Peptidase M1 N-terminal domain

RECEPTORS

PAF receptor

AAB25755	Pocillopora damicornis Annotation Release 100 proteins	XP_027041530	3.00E-20	query: platelet-activating factor receptor [Homo sapiens]
XP_027041530	model organisms landmark	NP_573455	4.00E-43	neuropeptide FF receptor 2 [Mus musculus]
XP_027041530	uniProtKB/Swiss-Prot	Q924H0	3.00E-43	neuropeptide FF receptor 2 [Mus musculus]
XP_027041530	conserved domains CDD v 36 50369 PSSMs	cd14993	5.33E-63	(7tmA_CCKR-like) cholecystokinin receptors and related proteins

leukotriene B4 receptor

O88855	Pocillopora damicornis Annotation Release 100 proteins	XP_027042015 XP_027042016	4.00E-22	query : Mouse Leukotriene B4 receptor 1 Ltb4r

XP_027042016	uniProtKB/Swiss-Prot	P30975.2	3.00E-40	Receptor for tachykinin-like peptides
XP_027042016	conserved domains CDD v 36 50369 PSSMs	cl28897	8.75E-60	seven-transmembrane G protein-coupled receptor superfamily
Q9JJL9	Pocillopora damicornis Annotation Release 100 proteins	XP_027046931	8.00E-15	query : Mouse Leukotriene B4 receptor 2 Ltb4r2
XP_027046931	uniProtKB/Swiss-Prot	P43141	2.00E-29	Beta-4C adrenergic receptor Meleagris
XP_027046931	conserved domains CDD v 36 50369 PSSMs	cd00637	2.16E-53	rhodopsin receptor-like class A family of the seven-transmembrane G protein-coupled receptor
**leukotriene D4 receptor**				
Q99JA4	Pocillopora damicornis Annotation Release 100 proteins	XP_027054275	5.00E-23	query : Mouse Cysteinyl leukotriene receptor 1
Q6NS65	Pocillopora damicornis Annotation Release 100 proteins	XP_027055033	2.00E-22	query : Mouse Uracil nucleotide/cysteinyl leukotriene receptor
XP_027054275	uniProtKB/Swiss-Prot	Q6BD04	2.00E-30	G-protein coupled receptor 54 Oreochromis niloticus
XP_027054275	conserved domains CDD v 36 50369 PSSMs	cd14993	1.00E-65	cholecystokinin receptors and related proteins,
XP_027055033	uniProtKB/Swiss-Prot	P58307	2.00E-45	Orexin receptor type 1 Mus musculus
XP_027055033	conserved domains CDD v 36 50369 PSSMs	cd14993	1.31E-86	cholecystokinin receptors and related proteins,
**LPA receptors**				
P61793	Pocillopora damicornis Annotation Release 100 proteins	XP_027039632	7.00E-27	Query : LPA receptor 1 Mus musculus
XP_027039632	uniProtKB/Swiss-Prot	P35406	2.00E-43	D(1) dopamine receptor Carassius
XP_027039632	conserved domains CDD v 36 50369 PSSMs	cd14967	4.62E-86	amine receptors and similar proteins
XP_027039632	model organisms landmark	XP_005158584	2.00E-44	D(1)-like dopamine receptor [Danio rerio]
Q9JL06	Pocillopora damicornis Annotation Release 100 proteins	XP_027044791	1.00E-13	query : LPA receptor 2 Mus musculus

XP_027044791	uniProtKB/Swiss-Prot	P97292.2	1.00E-38	Histamine H2 receptor Mus musculus
XP_027044791	conserved domains CDD v 36 50369 PSSMs	cd14967	1.46E-64	amine receptors and similar proteins
XP_027044791	model organisms landmark	NP_001010973	2.00E-38	Histamine H2 receptor Mus musculus
Q9EQ31	Pocillopora damicornis Annotation Release 100 proteins	XP_027047729	2.00E-28	Query : LPA receptor 3 Mus musculus
XP_027047729	uniProtKB/Swiss-Prot	Q9TST5	1.00E-39	Beta-2 adrenergic receptor Felis catus
XP_027047729	conserved domains CDD v 36 50369 PSSMs	cd00637	5.57E-64	rhodopsin receptor-like class A family of the seven-transmembrane G protein-coupled receptor
XP_027047729	model organisms landmark	NP_000015	2.00E-38	beta-2 adrenergic receptor [Homo sapiens]
Q8BLG2	Pocillopora damicornis Annotation Release 100 proteins	XP_027055033	5.00E-21	Query : LPA receptor 4 Mus musculus
XP_027055033	uniProtKB/Swiss-Prot	P58307.3	2.00E-45	Orexin receptor type 1 Mus musculus
XP_027055033	conserved domains CDD v 36 50369 PSSMs	cd14993	1.00E-86	(7tmA_CCKR-like) cholecystokinin receptors and related proteins
XP_027055033	model organisms landmark	NP_001098579	1.00E-46	neuropeptide FF receptor 2a [Danio rerio]
Q149R9	Pocillopora damicornis Annotation Release 100 proteins	XP_027050324	3.00E-16	Query : LPA receptor 5 Mus musculus
XP_027050324	uniProtKB/Swiss-Prot	Q9EQD2	2.00E-49	Neuropeptide FF receptor 2 Rattus norvegicus
XP_027050324	conserved domains CDD v 36 50369 PSSMs	cd14993	1.27E-74	(7tmA_CCKR-like) cholecystokinin receptors and related proteins
XP_027050324	model organisms landmark	NP_001098579	3.00E-49	neuropeptide FF receptor 2a [Danio rerio]
P32250	Pocillopora damicornis Annotation Release 100 proteins	XP_027050324	2.00E-22	Query : LPA receptor 6 Gallus gallus
XP_027050324	uniProtKB/Swiss-Prot	Q9EQD2	2.00E-49	Neuropeptide FF receptor 2 Rattus norvegicus
XP_027050324	conserved domains CDD v 36 50369 PSSMs	cd14993	1.27E-74	(7tmA_CCKR-like) cholecystokinin receptors and related proteins

XP_027050324	model organisms landmark	NP_001098579	3.00E-49	neuropeptide FF receptor 2a [Danio rerio]
XP_015779402	Pocillopora damicornis Annotation Release 100 proteins	XP_027044989	3.00E-62	Query: LPA receptor A. digitifera (Kegg)
XP_027044989	uniProtKB/Swiss-Prot	P30938.2	3.00E-08	Somatostatin receptor type 5 R norvegicus
XP_027044989	conserved domains CDD v 36 50369 PSSMs	cd00637	7.04E-28	rhodopsin receptor-like class A family of the seven-transmembrane G protein-coupled receptor
	model organisms landmark	XP_695365	3.00E-08	Somatostatin receptor type 5 R norvegicus
XP_015779402	Pocillopora damicornis Annotation Release 100 proteins	XP_027057660	8.00E-57	Query: LPA receptor A. digitifera (Kegg)
XP_027057660	uniProtKB/Swiss-Prot	Q5QD23	9.00E-09	Query : annotated as P. damicornis lysophosphatidic acid receptor 1-A-like. Hit Trace amine-associated receptor 5 R Norvegicus
XP_027057660	conserved domains CDD v 36 50369 PSSMs	cd00637	5.00E-22	rhodopsin receptor-like class A family of the seven-transmembrane G protein-coupled receptor
XP_027057660	model organisms landmark	NP_001076372	4.00E-11	trace amine-associated receptor 10b [Danio rerio]
XP_027059229	uniProtKB/Swiss-Prot	Q9EQ31	9.00E-08	Query : annotated as P. damicornis lysophosphatidic acid receptor 3-like. Hit Mouse LPA receptor 3
XP_027059229	conserved domains CDD v 36 50369 PSSMs	cd00637	1.28E-24	rhodopsin receptor-like class A family of the seven-transmembrane G protein-coupled receptor
XP_027059229	model organisms landmark	NP_075359	1.00E-07	Mouse LPA receptor 3
XP_027057060	uniProtKB/Swiss-Prot	Q9TUP7	4.00E-13	Query : annotated as P. damicornis lysophosphatidic acid receptor 6-like. Hit Dog Orexin receptor type 2

XP_027057060	conserved domains CDD v 36 50369 PSSMs	cd00637	1.64E-25	rhodopsin receptor-like class A family of the seven-transmembrane G protein-coupled receptor
XP_027057060	model organisms landmark	NP_001517.2	7.00E-13	orexin receptor type 2 [Homo sapiens]
XP_027055414	uniProtKB/Swiss-Prot	Q9Y5Y3.2	4.00E-12	Query : annotated as P. damicornis High affinity lysophosphatidic acid receptor-like. Hit Probable G-protein coupled receptor 45 Human
XP_027055414	conserved domains CDD v 36 50369 PSSMs	cd00637	1.23E-32	rhodopsin receptor-like class A family of the seven-transmembrane G protein-coupled receptor
XP_027055414	model organisms landmark	XP_005167494	3.00E-14	probable G-protein coupled receptor 45 [Danio rerio]
XP_027051925	uniProtKB/Swiss-Prot	Q9EQ31	8.00E-11	Query : lysophosphatidic acid receptor 3-like. Hit Lysophosphatidic acid receptor 3 Mus musculus
XP_027051925	conserved domains CDD v 36 50369 PSSMs	cd14967	3.38E-28	amine receptors and similar proteins
XP_027051925	model organisms landmark	NP_075359	1.00E-10	Mouse LPA receptor 3
XP_027051880	uniProtKB/Swiss-Prot	P46616	5.00E-08	Query : lysophosphatidic acid receptor 3-like. Adenosine receptor A2a Cavia
XP_027051880	conserved domains CDD v 36 50369 PSSMs	cd00637	4.52E-26	rhodopsin receptor-like class A family of the seven-transmembrane G protein-coupled receptor
XP_027051880	model organisms landmark	NP_001076578	2.00E-07	trace amine associated receptor 12a [Danio rerio]
XP_027051553	uniProtKB/Swiss-Prot	Q9Y5Y3	1.00E-08	Query : annotated as P. damicornis High affinity lysophosphatidic acid receptor-like. Hit Probable G-protein coupled receptor 45

XP_027051553	uniProtKB/Swiss-Prot	P79945	9.00E-07	Query : annotated as P. damicornis High affinity lysophosphatidic acid receptor-like. Hit High-affinity lysophosphatidic acid receptor Xenopus
XP_027051553	conserved domains CDD v 36 50369 PSSMs	cd00637	9.95E-26	rhodopsin receptor-like class A family of the seven-transmembrane G protein-coupled receptor
XP_027051553	model organisms landmark	NP_009158.3	1.00E-08	Probable G-protein coupled receptor 45 Human
XP_027050252	uniProtKB/Swiss-Prot	P18841	1.00E-11	Query : annotated as P. damicornis High affinity lysophosphatidic acid receptor-like. Hit Alpha-1B adrenergic receptor
XP_027050252	conserved domains CDD v 36 50369 PSSMs	cd00637	3.06E-32	rhodopsin receptor-like class A family of the seven-transmembrane G protein-coupled receptor
XP_027050252	model organisms landmark	NP_001076373	3.00E-12	trace amine-associated receptor 1 [Danio rerio]
XP_027047689	uniProtKB/Swiss-Prot	P43141	2.00E-10	Query : annotated as P. damicornis lysophosphatidic acid receptor 3-like. HitBeta-4C adrenergic receptor Meleagris
XP_027047689	conserved domains CDD v 36 50369 PSSMs	cd00637	1.09E-31	rhodopsin receptor-like class A family of the seven-transmembrane G protein-coupled receptor
XP_027047689	model organisms landmark	XP_017213593.2	5.00E-10	trace amine-associated receptor 13c-like [Danio rerio]
XP_027046799	uniProtKB/Swiss-Prot	P43141	2.00E-04	Query : annotated as P. damicornis lysophosphatidic acid receptor 1B-like. Hit Adenosine receptor A3. Canis lupus familiaris
XP_027046799	conserved domains CDD v 36 50369 PSSMs	cd00637	3.71E-23	rhodopsin receptor-like class A family of the seven-transmembrane G protein-coupled receptor
XP_027046799	model organisms landmark	XP_017213593.2	5.00E-10	trace amine-associated receptor 13c-like [Danio rerio]

XP_027059981	uniProtKB/Swiss-Prot	Q9Y5Y3.2	5.00E-83	Query : annotated as P. damicornis High affinity lysophosphatidic acid receptor-like. Hit Probable G-protein coupled receptor 45 Human
XP_027059981	uniProtKB/Swiss-Prot	Q9BZJ6.2	2.00E-82	Probable G-protein coupled receptor 63
XP_027059981	uniProtKB/Swiss-Prot	Q9EQQ3.2	5.00E-82	Probable G-protein coupled receptor 63
XP_027059981	uniProtKB/Swiss-Prot	P79945	5.00E-79	High-affinity lysophosphatidic acid receptor Xenopus
XP_027059981	uniProtKB/Swiss-Prot	Q9EQQ4	1.00E-78	Probable G-protein coupled receptor 45
XP_027059981	conserved domains CDD v 36 50369 PSSMs	cd15213	2.82E-100	G protein-coupled receptor PSP24 and similar proteins,
XP_027059981	model organisms landmark	XP_005167494	1.00E-87	probable G-protein coupled receptor 45 [Danio rerio]
**Pocillopora damicornis putative LPA receptors**				
XP_027059229	Pocillopora damicornis Annotation Release 100 proteins	XP_027059202<br>XP_027059198<br>XP_027059200<br>XP_027059201	7.00E-55	rhodopsin, GQ-coupled-like [Pocillopora damicornis]
XP_027059229	Pocillopora damicornis Annotation Release 100 proteins	XP_027059209<br>XP_027059210	1.00E-27	muscarinic acetylcholine receptor M3-like [Pocillopora damicornis]
XP_027051925	Pocillopora damicornis Annotation Release 100 proteins	XP_027051880	0.00E+00	lysophosphatidic acid receptor 3-like [Pocillopora damicornis]
XP_027051925	Pocillopora damicornis Annotation Release 100 proteins	XP_027051881	5.00E-141	5-hydroxytryptamine receptor 1D-like [Pocillopora damicornis]
XP_027051925	Pocillopora damicornis Annotation Release 100 proteins	XP_027051902	1.00E-136	sphingosine 1-phosphate receptor 2-like [Pocillopora damicornis]
XP_027051925	Pocillopora damicornis Annotation Release 100 proteins	XP_027051882	1.00E-126	adenosine receptor A2a-like [Pocillopora damicornis]

XP_027051925	Pocillopora damicornis Annotation Release 100 proteins	XP_027057545	2.00E-115	adenosine receptor A2a-like [Pocillopora damicornis]
XP_027051925	Pocillopora damicornis Annotation Release 100 proteins	XP_027054460	1.00E-111	melanocortin receptor 4-like [Pocillopora damicornis]
XP_027051925	Pocillopora damicornis Annotation Release 100 proteins	XP_027052671	1.00E-105	alpha-1A adrenergic receptor-like [Pocillopora damicornis]+G232
XP_027051925	Pocillopora damicornis Annotation Release 100 proteins	XP_027048970	4.00E-104	beta-2 adrenergic receptor-like [Pocillopora damicornis]
XP_027051925	Pocillopora damicornis Annotation Release 100 proteins	XP_027052672	8.00E-103	alpha-1A adrenergic receptor-like [Pocillopora damicornis]
XP_027051925	Pocillopora damicornis Annotation Release 100 proteins	XP_027057387	5.00E-108	adenosine receptor A2a-like [Pocillopora damicornis]
XP_027051553	Pocillopora damicornis Annotation Release 100 proteins	XP_027051548	0.00E+00	adenosine receptor A3-like [Pocillopora damicornis]
XP_027059981	Pocillopora damicornis Annotation Release 100 proteins	XP_027053973	2.00E-39	octopamine receptor beta-2R-like [Pocillopora damicornis]
XP_027059202	model organisms landmark	XP_021331324	1.00E-11	lysophosphatidic acid receptor 2b isoform X2 [Danio rerio]
XP_027059209	model organisms landmark	XP_693932.2	8.00E-22	adenosine receptor A1-like [Danio rerio]
XP_027051880	model organisms landmark	see above	see above	see above
XP_027051881	model organisms landmark	XP_001920844.2	1.00E-12	trace amine-associated receptor 4 [Danio rerio]
XP_027051902	model organisms landmark	NP_034463.2	8.00E-12	sphingosine 1-phosphate receptor 2 [Mus musculus]
XP_027051882	model organisms landmark	NP_031446.2	2.00E-13	beta-2 adrenergic receptor [Mus musculus]
XP_027057545	model organisms landmark	NP_001076563	7.00E-07	trace amine associated receptor 12e [Danio rerio]
XP_027054460	model organisms landmark	XP_005159638	3.00E-11	melanocortin receptor 5 isoform X1 [Danio rerio]
XP_027052671	model organisms landmark	NP_001076578	4.00E-15	trace amine associated receptor 12a [Danio rerio]
XP_027048970	model organisms landmark	NP_001076578	5.00E-10	trace amine associated receptor 12a [Danio rerio]
XP_027052672	model organisms landmark	NP_001076578	9.00E-14	trace amine associated receptor 12a [Danio rerio]

XP_027057387	model organisms landmark	XP_009303915.2	2.00E-08	trace amine-associated receptor 13c-like [Danio rerio]
XP_027051548	model organisms landmark	NP_009158.3	2.00E-06	probable G-protein coupled receptor 45 [Homo sapiens]
XP_027053973	model organisms landmark	XP_697043.2	9.00E-54	alpha-1D adrenergic receptor [Danio rerio]
